# Pangenomic analysis of Chinese gastric cancer

**DOI:** 10.1038/s41467-022-33073-7

**Published:** 2022-09-15

**Authors:** Yingyan Yu, Zhen Zhang, Xiaorui Dong, Ruixin Yang, Zhongqu Duan, Zhen Xiang, Jun Li, Guichao Li, Fazhe Yan, Hongzhang Xue, Du Jiao, Jinyuan Lu, Huimin Lu, Wenmin Zhang, Yangzhen Wei, Shiyu Fan, Jing Li, Jingya Jia, Jun Zhang, Jun Ji, Pixu Liu, Hui Lu, Hongyu Zhao, Saijuan Chen, Chaochun Wei, Hongzhuan Chen, Zhenggang Zhu

**Affiliations:** 1 Department of General Surgery of Ruijin Hospital, Shanghai Institute of Digestive Surgery, and Shanghai Key Laboratory for Gastric Neoplasms, Shanghai Jiao Tong University School of Medicine, 200025 Shanghai, China; 2 Department of Radiation Oncology and Department of Oncology, Shanghai Medical College, Fudan University Shanghai Cancer Center, 270 Dong An Road, Shanghai, 200032 China; 3 Department of Bioinformatics and Biostatistics, School of Life Sciences and Biotechnology, Shanghai Jiao Tong University, 800 Dongchuan Road, Shanghai, 200240 China; 4 SJTU-Yale Joint Center for Biostatistics and Data Science, Shanghai Jiao Tong University, 800 Dongchuan Road, Shanghai, 200240 China; 5 Department of Oncology, Ruijin Hospital, Shanghai Jiao Tong University School of Medicine, 200025 Shanghai, China; 6 Institute of Cancer Stem Cell, Dalian Medical University, Dalian, 116044 China; 7 National Facility for Translational Medicine (Shanghai), The Institute of Translational Medicine, Shanghai Jiao Tong University, 200025 Shanghai, China; 8 Shanghai Collaborative Innovation Center of Translational Medicine, Shanghai Jiao Tong University School of Medicine, 227 South Chongqing Road, Shanghai, 200025 China; 9 Institute of Interdisciplinary Integrative Medicine Research, Shuguang Hospital, Shanghai University of Traditional Chinese Medicine, Shanghai, 201203 China

**Keywords:** Surgical oncology, Cancer genomics, Gastric cancer, Genome assembly algorithms, Cancer genetics

## Abstract

Pangenomic study might improve the completeness of human reference genome (GRCh38) and promote precision medicine. Here, we use an automated pipeline of human pangenomic analysis to build gastric cancer pan-genome for 185 paired deep sequencing data (370 samples), and characterize the gene presence-absence variations (PAVs) at whole genome level. Genes *ACOT1*, *GSTM1, SIGLEC14* and *UGT2B17* are identified as highly absent genes in gastric cancer population. A set of genes from unaligned sequences with GRCh38 are predicted. We successfully locate one of predicted genes *GC0643* on chromosome 9q34.2. Overexpression of *GC0643* significantly inhibits cell growth, cell migration and invasion, cell cycle progression, and induces cell apoptosis in cancer cells. The tumor suppressor functions can be reversed by sh*GC0643* knockdown. The *GC0643* is approved by NCBI database (GenBank: MW194843.1). Collectively, the robust pan-genome strategy provides a deeper understanding of the gene PAVs in the human cancer genome.

## Abstract (web summary)

Human pan-genomics are increasing our knowledge of genomic diversity and genetic factors in disease. Here, the authors built a gastric cancer pan-genome that included the sequences of Chinese Han patients, and predicted putative and previously unaligned genes associated with gastric cancer.

## Introduction

Since its initial release 20 years ago, the human reference genome (current version GRCh38) has significantly promoted a wide range of biomedical research^1^. At present, almost all published high-throughput genomic studies are based on the “map-to-single-reference genome strategy”. However, the reference genome produced by the Human Genome Project was sequenced from a small number of individual samples and did not reflect the complete genomic status of diverse populations. In fact, the human reference genome is still incomplete^2^. A recent study found that there are 819 incoherent gaps in the human reference genome, and some long fragmental sequences from the large population could not match the current human reference genome^3^. In recent years, scientists have explored a new methodology, the pangenomics approach, to study the missed sequences of the reference genome^4,5^. Pangenomics was first introduced in 2005 as the collection of the genes of a population of microbial organisms and it studied the patterns of gene presence and absence across individual samples^6^. The concept was then extended to plant and animal studies^7,8^. Although any structure variation study could also be considered a pan-genome study in a broad sense, one of the prevalent methods to present a pan-genome was using the reference genome plus those unaligned sequences, including partially unaligned and fully unaligned^9^. The first human pan-genome study in this approach was published in 2010. That study analyzed the whole-genome sequences (WGS) of one Asian and one African individual, and then compared the differences between the sequences of the two individuals to the human reference genome. That study indicated that at least 19–40 Mbp new sequences were missed in the human reference genome^10^. Sherman et al. reported a pan-genome assembled from the deep sequencing of 910 humans with African ancestry, and found that 296 Mbp sequences were unaligned with the human reference genome^8^. A pan-genome contains two types of genes, core genes shared by all individuals and distributed genes shared by some but not all individuals. The latter type of genes that do not exist in all individuals are also called gene presence-absence variations (PAVs)^9^. The gene PAVs are the special type of variations in pangenomics. In our previous study, we developed a HUman Pan-genome ANalysis (HUPAN) tool for constructing human pan-genomes from WGS data and characterizing the gene PAVs harbored in the human genomes^11^. However, the potential genes and biological functions of unaligned sequences remained unclear, which, on the other side, could be important to tumor study.

In this work, we analyze the deep sequencing data of WGS from 185 paired (370 samples) gastric cancer and normal tissues by HUPAN, and characterize the PAVs landscape of human gastric cancer. A predicted gene *GC0643* on chromosome 9q34.2 is identified as a tumor suppressor.

## Results

### GCPAN construction

**Fig. 1:**
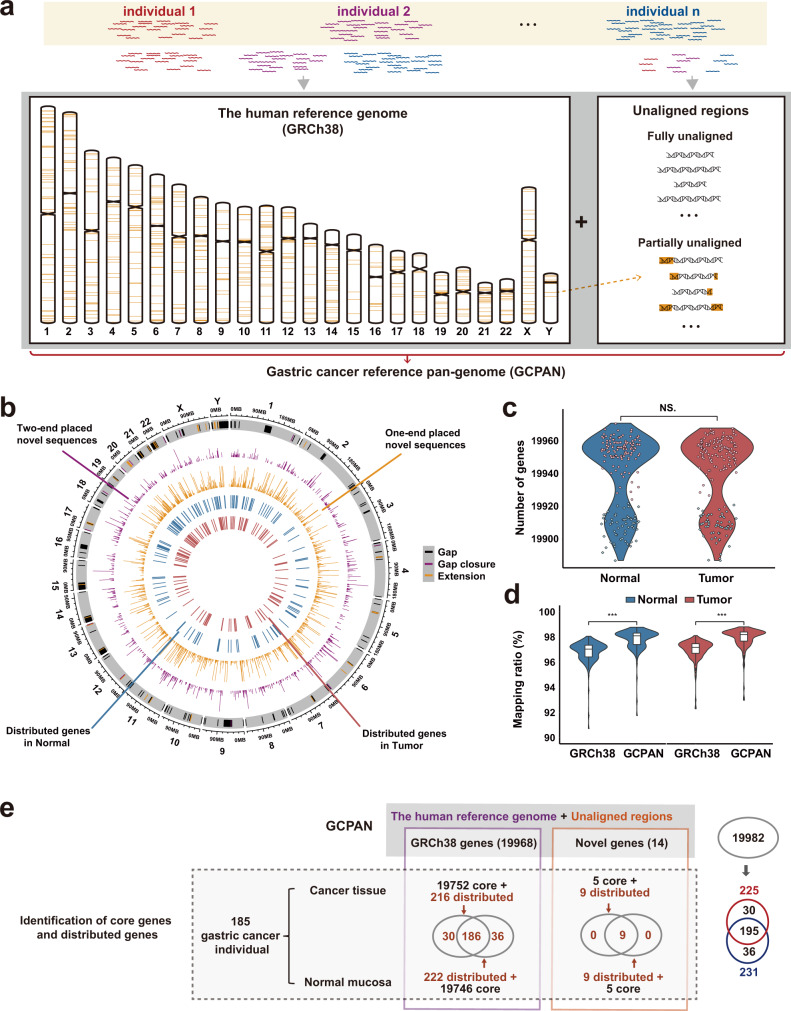
The composition of GCPAN. **a** GCPAN contains two parts, the human reference genome GRCh38, and unaligned sequences. The 24 chromosomes represent GRCh38, and the sequences on the right side represent unaligned sequences including fully unaligned sequences and partially unaligned sequences. The later parts were shown as orange bars on the chromosomes of the left side (only sequences longer than 2000 bp are marked on chromosomes). **b** Distribution of distributed genes and sequences on 22 autosomes and two sex chromosomes. The heights of the bars represent the sequence lengths. The gap, gap closure, and gap extension were shown in the out-most circle. **c** There was no significant difference in gene numbers between normal mucosae and cancer tissues (paired *t*-test). Normal: normal mucosae; Tumor: cancer tissues. **d** Comparison of mapping ratio of sequencing data from 185 samples using pan-genome versus GRCh38. The reads mapping rates were significantly increased by pan-genome mapping, compared to GRCh38 mapping (paired *t*-test). The center lines of the box plots show median values, hinges the first and third quartiles, and the whiskers the maxima and minima within 1.5 times of the interquartile range. ***P ≤ 0.001. **e** The diagram of GCPAN from 185 individuals. The numbers of core genes and distributed genes are presented in GRCh38 box, while the numbers of core genes and distributed genes for predicted new sequences are shown in the novel gene box. The total numbers of genes shared by cancer and normal mucosa are shown on the right side.

We applied HUPAN to analyze the WGS data from 185 paired (370 samples) gastric cancers and normal tissues. Short sequencing reads were assembled into contigs with SGA, and aligned back to GRCh38 with MUMMER. Unaligned sequences were masked with RepeatMasker and then annotated for genes with MAKER. A gastric cancer pan-genome (GCPAN) including GRCh38 and 80.88 Mbp sequences unaligned to GRCh38 was constructed. The unaligned sequences include 53.78 Mbp fully unaligned regions and 27.10 Mbp partially unaligned regions (Fig. 1a). The partially unaligned sequences include 827 two-end placed sequences and 1778 one-end placed sequences distributed across GRCh38 (Fig. 1b). The partially unaligned sequences intersected with 18 protein-coding genes, including six genes (*BOD1, MUC6, OR8U1, HLA-DRB5, HLA-DRB1*, and *GOLGA6L2*) at CDS regions and 15 genes (*ABO, AP2A2, BOD1, C8orf34, GOLGA6L2, HLA-DRB1, HLA-DRB5, KRBOX4, MAN1B1, MEIOB, MOGAT2, MTO1, PRKRA, ROBO1*, and *TMEM68*) at untranslated regions (UTR regions) (Supplementary Table 5). Although some of the genes might be missing with short-read sequencing data, we originally predicted 82 genes on non-reference sequences, and 14 of these 82 genes contained less than 50% of repetitive regions. Of these 14 predicted genes, 12 were on the partially unaligned contigs. The average number of genes present in an individual genome is 19,939 (19,928 on GRCh38 and 11 on non-reference sequences) in gastric cancer (Fig. 1c). The reads mapping rates using GRCh38 and GCPAN as the reference were 97.16 and 98.19%, respectively (*P* < 0.001, Fig. 1d).

### PAVs of GRCh38 gene and pathway analysis

**Fig. 2:**
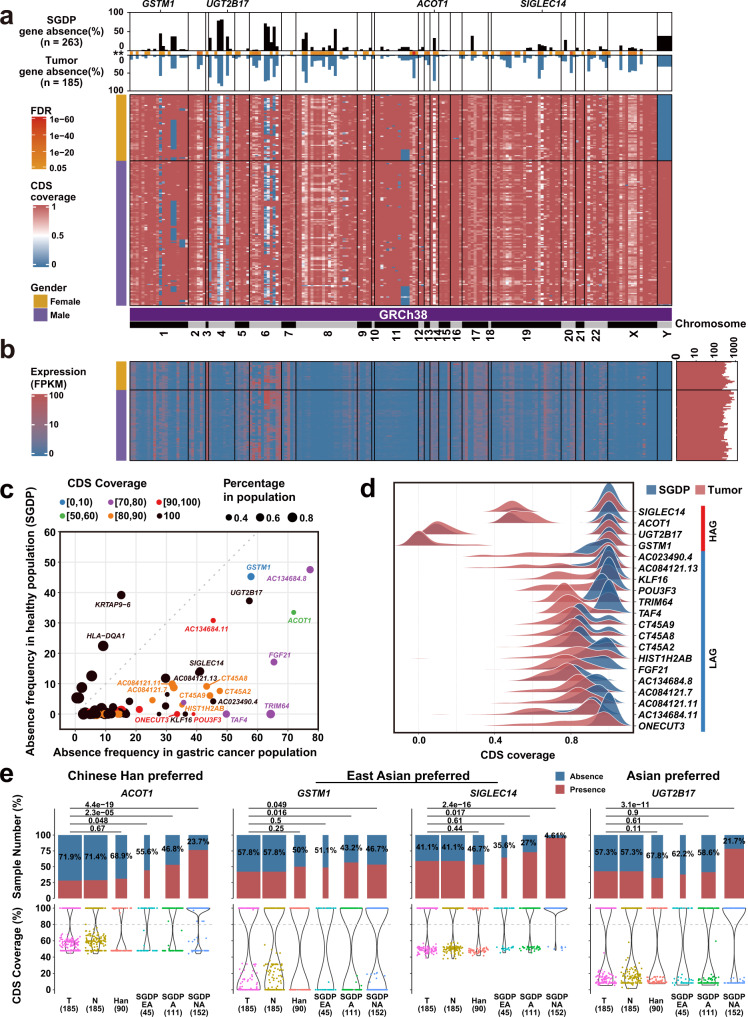
The PAVs landscape of 186 distributed genes on GRCh38 genes in gastric cancer. **a** PAV profile of distributed genes. The top bar plot shows different frequencies of absence variation of distributed genes in SGDP group (black bar) and gastric cancer group (blue bar). The heatmap shows the CDS coverage of distributed genes. The gender phenotype is listed on the left side of the heatmaps. The genes located on GRCh38 are sorted by the physical positions of chromosomes. **b** The mRNA expression of distributed genes was validated by RNA-seq in 87 cancers. **c** The distribution features of 78 differential distributed genes between gastric cancer and SGDP groups. X-axis represents the absence frequency of genes in the gastric cancer group (CDS coverage <80%). Y-axis indicates the absence frequency of genes in the SGDP group. The dots in the figure represent genes. The dot color is determined by the largest proportion of a gene’s CDS coverage. Black color means 100% CDS coverage. **d** The top 20 distributed genes could be divided into highly absent genes (HAG: *SIGLEC14*, *ACOT1*, *UGT2B17*, and *GSTM1*) and low absent genes (LAG: others). **e** Comparison of gene absence frequencies in different populations for the four HAGs (Fisher’s exact test). T: cancer; N: Normal; Han: the 90 Han Chinese dataset; SGDP EA: East Asian in SGDP dataset, A: Asian in SGDP dataset, NA: Non-Asian in SGDP dataset.

We characterized 261 distributed genes that exist in some but not all individuals. Among them, 195 distributed genes (186 on GRCh38 and 9 predicted new genes) were shared in both tumors and normal tissues (Fig. 1e and Supplementary Tables 9 and 10). Other 36 and 30 distributed genes were absent in normal and tumor tissues, respectively (Supplementary Tables 11 and 12). The ratio of distributed genes on GRCh38 is 1.08–1.11% (Supplementary Fig. 10). To find out cancer susceptible genes of the cancer population, we compared PAVs with those in two independent datasets. Of them, 263 individuals were from the Simons Genome Diversity Project (SGDP)^12^ and 90 individuals were from Han Chinese^13^. The PAV pattern and the corresponding mRNA expression of the 186 distributed genes of GRCh38 in gastric cancer are shown in Fig. 2a, b, respectively. The absence frequencies of 78 distributed genes in the cancer population are significantly different from the frequencies in SGDP datasets (false discovery rate (FDR) < 0.05) (Fig. 2c, d and Supplementary Table 13). We did not find the frequency difference of PAVs between GCPAN and 90 Hans. The top 20 genes with CDS coverage <50% are defined as highly absent genes (HAG: *GSTM1, UGT2B17, ACOT1*, and *SIGLEC14*), and others are low absent genes (CDS coverage >50%, LAG) (Fig. 2d). We calculated the odds ratio (OR) of these distributed genes, compared to the Asian population of SGDP dataset. The genes with OR > 1.5 suggesting their carcinogenic association are presented in Supplementary Fig. 19 and Supplementary Table 15. The absent frequencies of four HAG in SGDP, 90 Hans, and our group are presented in Fig. 2e. These genes showed significant absence in all Asian (*UGT2B17*), East Asian (*GSTM1* and *SIGLEC14*), or Han Chinese (*ACOT1*).

**Fig. 3:**
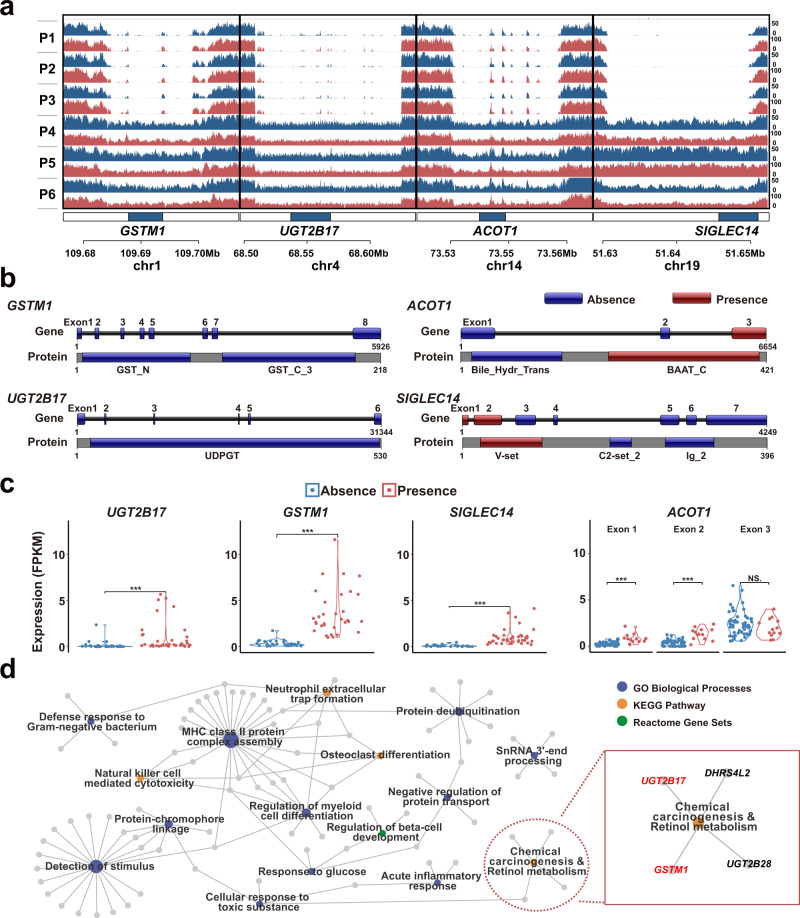
The paradigms of absence variation and functional enrichment of disotributed genes. **a** The absent coverage on corresponding chromosomal locations of *GSTM1*, *UGT2B17*, *ACOT1*, and *SIGLEC14* in three representative absence (P1–P3) and three presence (P4–P6) cancer samples. The tracks of blue and red stand for normal mucosa and cancer tissue, respectively. **b** The sketches of gene structures and conserved domains of protein-encoding sequences in InterPro database. The absent regions of genes are shown as blue bars. **c** Gene expression levels (FPKM) of four HAG genes in gastric cancers by RNA-Seq validation (Wilcox test). The expression of *ACOT1* is shown separately for its three exons. ***P ≤ 0.001; NS not significant. **d** Functional enrichment of 186 distributed genes revealed 16 pathways. Two highly absent genes *GSTM1* and *UGT1B17* were enriched in the chemical carcinogenesis pathway. The colored dots represent pathways and gray dots represent genes. The sizes of colored dots represent the number of genes involved.

The four highly absent genes (*GSTM1, UGT2B17, ACOT1*, and *SIGLEC14*) with CDS coverage less than 50% in representative cases are presented in Fig. 3a. In sequence structures analysis, *GSTM1* and *UGT2B17* are completely absent, while *ACOT1* and *SIGLEC14* are partially absent (Fig. 3b and Supplementary Fig. 22). The decreased expression was verified in three out of four genes (*GSTM1, UGT2B17*, and *SIGLEC14*) by RNA-seq (Fig. 3c). The 186 distributed genes on GRCh38 are enriched in 16 pathways (Fig. 3d and Supplementary Table 16). Notably, two highly absent genes *GSTM1* and *UGT2B17* are enriched in the chemical carcinogenesis pathway. The gene PAVs are attributed to the increased carcinogenic risk and clinical phenotypes of gastric cancer (Supplementary Figs. 23–30).

### PAVs of predicted genes

**Fig. 4:**
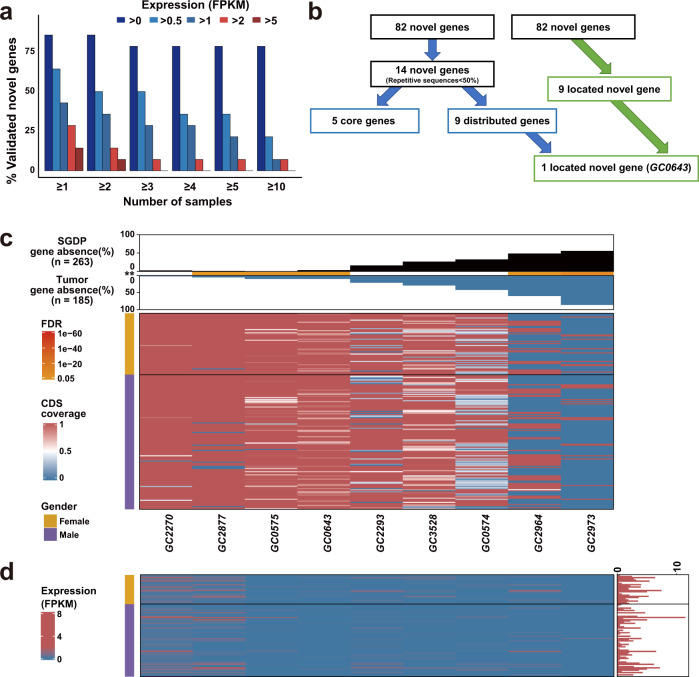
The PAVs feature of nine predicted new genes in gastric cancer using GCPAN as the reference. **a** The mRNA transcription (>1 FKPM) in 6 out of 14 predicted new genes was confirmed in at least one cancer sample. **b** Illustration of the alignment tree of 82 predicted new genes. **c** PAVs profile of predicted new genes. The top bar plot shows the different frequencies of absence variation in SGDP dataset (black bar) and gastric cancer group (blue bar). The orange line between the two cohorts indicates the significant difference of absence variation between the two groups. The heatmap shows the CDS coverage of predicted new genes. **d** The mRNA expression validation of predicted new genes by RNA-seq. The predicted new genes are sorted by gene absence frequencies. The red bar chart on the right side represents the total number of validated genes in each sample. The gender phenotype is listed on the left side of the heatmaps. The predicted new genes were expressed at low levels in most cases.

Using the current GCPAN, 82 protein-coding genes were predicted from non-GRCh38 sequences. Among these 82 genes, 14 predicted genes contain less than 50% of repetitive elements and are present in at least 26 out of 185 (14.05%) tumor tissues (Supplementary Table 4). We examined the mRNA expression in 87 cancer tissues by RNA-seq. The mRNA transcription (>1 FKPM) was confirmed in at least one gastric tumor tissue in 6 out of 14 (42.86%) predicted new genes (Fig. 4a). Nine out of 82 predicted new genes could be located by long-read sequencing of the third-generation sequencing. By PAVs analysis, 9 out of 14 (64.29%) predicted genes belong to distributed genes (Fig. 4b), and are shared in both tumors and normal tissues (Supplementary Fig. 10 and Supplementary Table 10). The PAVs pattern and the corresponding mRNA expression of the tumor are shown in Fig. 4c, d.

### Chromosome location of predicted gene *GC0643*

**Fig. 5:**
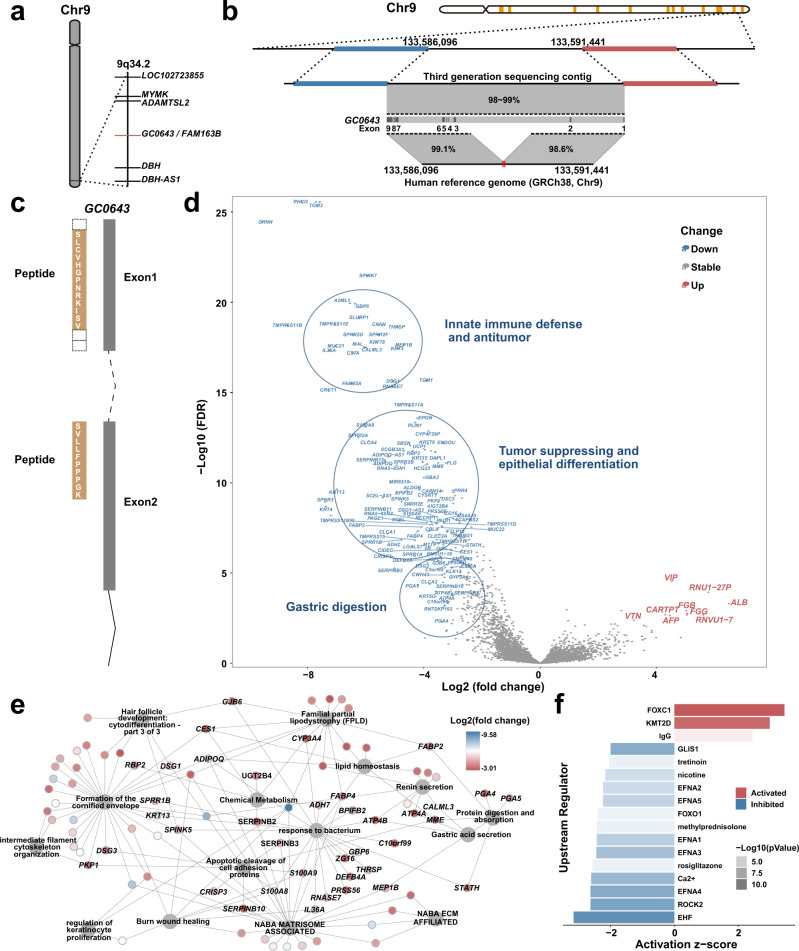
Chromosome location and functional enrichment of gene *GC0643*. **a** *GC0643* gene is localized on 9q34.2 by long-read sequencing analysis against GRCh38. The neighboring genes of *GC0643* are also presented. **b** The correlation of *GC0643* position with the SV breakpoints. Four long-read sequencing contigs support the chromosomal location of *GC0643*, and one of them is shown in the figure. The third-generation sequencing data reveal that a fragment of ~1700 bps is missing in GRCh38. SV analysis shows two SV breakpoints in the region (the two red dots between chr9:133,588,624 and chr9:133,588,681 shown on the bottom black line). **c** The mapping of GC0643 and a peptide sequence derived from CPTAC proteomic data. The peptides sequence is supported by at least two cases. The peptides are matched to the protein-coding regions of exons 1 and 2. **d** The volcano plot of differentially expressed genes between *GC0643* absence and presence groups. The upregulated genes are shown in red (right), and the downregulated genes are shown in blue (left). The important gene clusters are circled and marked in the plot. **e** The pathway enrichment of downregulated genes is presented. A total of 15 pathways are enriched. **f** The 17 upstream regulators of *GC0643* gene based on ingenuity pathway analysis.

By eliminating the genes containing over 50% repeat sequences, only 14 of the 82 genes remained. Nine of these 14 genes were considered distributed and only one of them was among the 9 genes with chromosomal loci determined using long-read sequencing data (see Fig. 4b for more details). The gene was *GC0643* at 9q34.2, overlapped with some intron sequences of *FAM163B* (Fig. 5a). We evaluated the structure variations (SVs) on *GC0643* chromosomal locus by traditional genomic analysis and recognized two SV breakpoints at 9q34.2 (Fig. 5b). Although traditional SV analysis gives the breakpoint position, SV type, and SV length, it gives no further information of the gene (Supplementary Fig. 31 and Supplementary Table 17). To validate the protein expression of *GC0643*, we searched *GC0643* protein sequence against the mass spectrum data from 80 diffuse gastric cancer in CPTAC database by X!Tandem (version 2017.2.14) and the human protein sequence database (GENECODE v30). The protein expression of *GC0643* gene was supported by peptide hitting (SLCVHGPNRKISVLLFPPPGK) in two gastric cancer cases (Fig. 5c).

We used CDS coverage of 80% as a cut-off and divided RNA-Seq data of 65 cases (among the 185 samples with WGS data) into gene absence (18 samples) and gene presence (47 samples) groups. At *GC0643* absence group, the 10 upregulated differential genes (3 fold-change, *P* < 0.001) are enriched in embryonic foregut development, cell migration, mitogen activity, and irritative response of liver, including *AFP, ALB, RNVU1-7, VTN, FGG, FGB*, and *VIP* (Fig. 5d and Supplementary Table 18). Whereas, the 138 downregulated genes of *GC0643* absence group are enriched in 15 pathways such as gastric digestion, integrity of epithelial cells, epithelial differentiation, protective mucous barriers, antitumor activity, chemical metabolism, innate immune defense, and others (Fig. 5d, e and Supplementary Table 18). We identified 17 upstream regulators of *GC0643* gene based on ingenuity pathway analysis (Fig. 5f and Supplementary Table 19).

### The biological function of gene *GC0643*

**Fig. 6:**
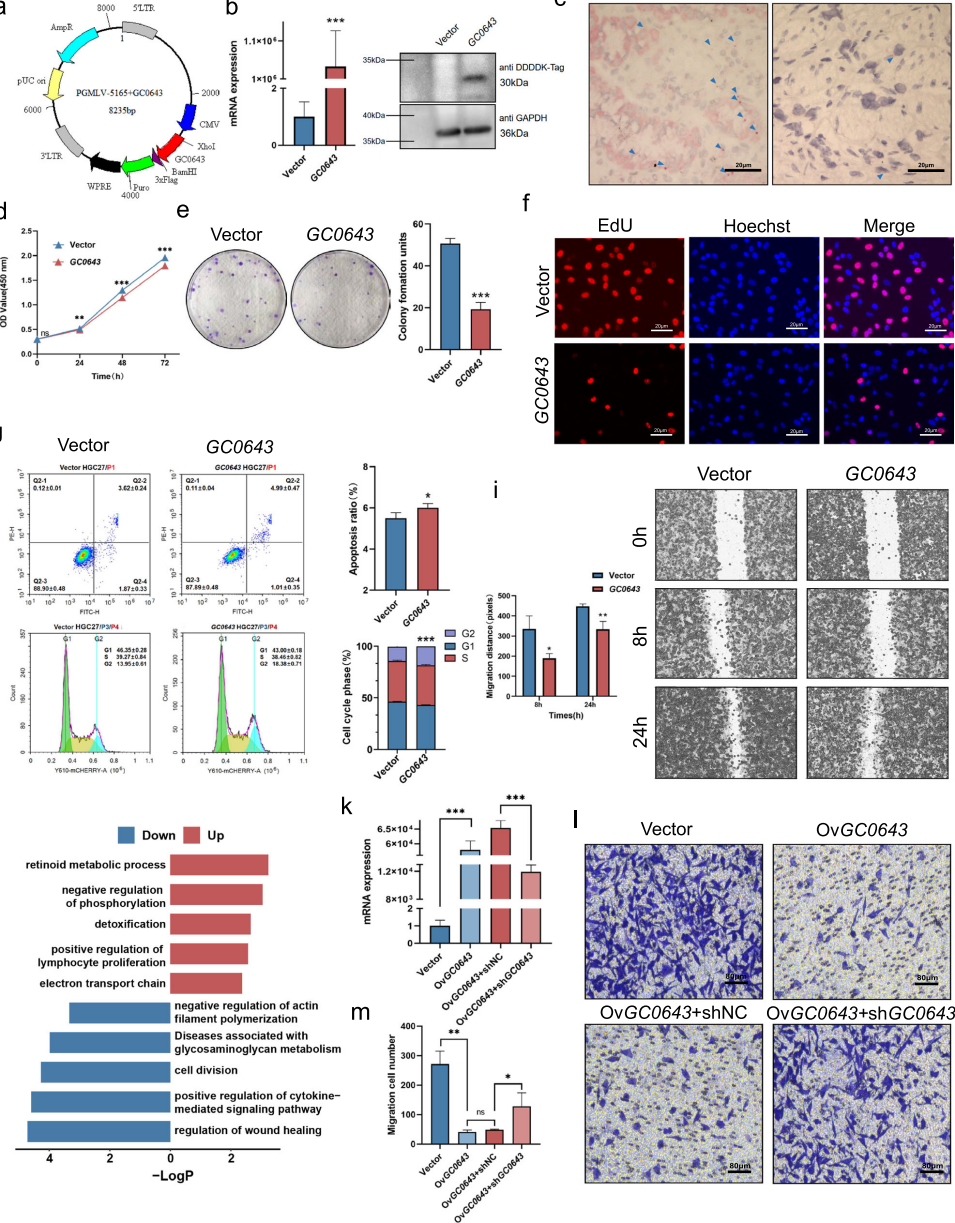
The biological functions of *GC0643* gene. **a** The schematic of PGMLV-*GC0643* construction. **b** Effect of *GC0643* gene transfection on HGC27 cancer cells, as monitored by qRT-PCR of *GC0643* mRNA expression fold change plot and western blot of anti-DDDDK-Tag. Data are presented as mean values ± SD from *n* = 3 biological replicates. Data were analyzed statistically by two-tailed Student’s *t*-test. ***P = 4.50E–05. **c** The mRNA expression of *GC0643* was examined by RNAScope. The positive signals (red dots) were observed in normal mucosa, but reduced in cancer tissue (right) (scale bar represents 20 μm). Representative images from *n* = 3 biological replicates. **d**, **e** Cell growth activity (CCK8) and colony formation (soft agar assays) are presented. **d** Data are presented as mean values ± SD of 5 biological replicates, and were analyzed by two-tailed Student’s *t*-test with ^ns^*P* = 0.547, **P = 0.002, ***P = 9.99E–07, ***P = 0.0008. **e** Data are presented as mean values ± SD of 3 biological replicates, and were analyzed by two-tailed Student’s *t*-test with **P = 0.0002. **f** The proliferating cell ratio examination by EdU (scale bar represents 20 μm). Representative images of EdU positive cells (red dots) from *n* = 5 biological replicates. **g**, **h** Enforced *GC0643* induced cell apoptosis and resulted in G2/M arrest. Data are presented as mean values ± SD of 5 biological replicates. *P* values were derived from two-tailed Student’s *t*-test with *P = 0.01; ***P = 1.27E–05. **i** Cell migratory ability was suppressed by scratch wound healing assays. *P* values were derived from two-tailed Student’s *t*-test with *P = 0.02; **P = 0.08. Data are presented as mean values ± SD of 3 biological replicates. **j** The changed downstream pathways based on *GC0643* overexpression. **k** The knockdown efficiency of shRNA for *GC0643* overexpressed cancer cells. *P* values were derived from two-tailed Student’s *t*-test with ***P = 5.81E-06; ***P = 4.26E–06. Data are presented as mean values ± SD of 3 biological replicates. **l**, **m** Cell migration ability are suppressed, but reversed after knockdown *GC0643* by shRNA. *P* values were derived from two-tailed Student’s *t*-test with ***P = 4.70E-05, ^ns^*P* = 0.067, *P = 0.013. Data are presented as mean values ± SD of 4 biological replicates.

Gene expression of *GC0643* was detected on both mRNA and protein levels after *GC0643* eukaryotic expression vector (Fig. 6a) was enforced in gastric cancer cell line HGC27 from Asian patient (Fig. 6b). The cytoplasmic location of *GC0643* in normal gastric epithelium is clearly detected but reduced in cancer cells by RNAscope examination (Fig. 6c and Supplementary Fig. 33). Overexpression of *GC0643* gene significantly inhibited the cell growth and colony formation. The OD value between vector and *GC0643* groups at 24 h (0.514 ± 0.005 vs 0.491 ± 0.009, *P* = 0.002), 48 h (1.295 ± 0.019 vs 1.146 ± 0.013, *P* = 9.99E–07), and 72 h (1.957 ± 0.032 vs 1.793 ± 0.053, *P* = 0.0008) was significantly different by CCK8 assay (Fig. 6d). The colony numbers between vector and *GC0643* groups were significantly different (50 ± 2 vs 19 ± 3, *P* = 0.0002, Fig. 6e). By EdU cell proliferation assay, the proliferating cell ratio (37.40 ± 5%) in *GC0643* group was significantly reduced, compared to vector group (47.90 ± 1.97%) (*P* = 0.0045, Fig. 6f). Compared to vector group, overexpression of *GC0643* induced cell apoptosis (5.49 ± 0.24% vs 6.00 ± 0.18%, *P* = 0.01, Fig. 6g), and resulted in G2/M arrest with increased G2/M fraction (13.95 ± 0.61% vs 18.38 ± 0.71%, *P* = 1.27E–05, Fig. 6h). In addition, upregulation of *GC0643* significantly inhibited cell migration ability. The cell migration distances at 8 h (335.33 ± 53.13 vs 190.00 ± 18.38, *P* = 0.02) and 24 h (448.00 ± 9.90 vs 333.00 ± 32.12, *P* = 0.008) were obviously shorten by the wound healing assay (Fig. 6i). In *GC0643* overexpressed cancer cells, pathways of lymphocyte activation and detoxification were upregulated and wound healing, cytokine-mediated signaling, and cell division were downregulated in transcriptome analysis (Fig. 6j and Supplementary Table 20). We knocked down the *GC0643* by shRNA in *GC0643* overexpressed cells and significantly inhibited the mRNA expression (645.00 ± 20.46 vs 118.67 ± 8.18, *P* = 4.26E–06, Fig. 6k). Compared to the controls, overexpression of *GC0643* significantly inhibited cell migration (271 ± 38 vs 41 ± 5, *P* = 0.0016), whereas after knockdown of *GC0643* gene by shRNA, the inhibition of cell migration was obviously reversed (shNC vs *GC0643* + sh*GC0643*, 48 ± 2 vs 128 ± 39, *P* = 0.0127, Fig. 6l, m and Supplementary Figs. 34 and 35). Similar effects of *GC0643* overexpression were also observed in NCI-N87 cancer cells from the western patient (Supplementary Fig. 36).

## Discussion

The advantages of pan-genome are to find out the non-reference genome sequences and construct the reference pan-genome (reference genome plus non-reference sequences) for a specific population. Using the reference pan-genome as the baseline, the deep sequencing data of each individual are mapped to the reference pan-genome for uncovering the gene PAVs. Pan-genome consists of core genes (shared by all individuals), distributed genes (shared by some but not all individuals), and unique genes (that are individual-specific). The latter two types of genes that do not exist in all individuals are called PAVs^9^. The PAVs are the special type of variations in pangenomics. Although previously published human pan-genomes showed a large number of non-reference genome sequences, but no systematic PAVs analysis has been done, especially for disease genomics. The reason may attribute to the requirement of huge computing resources for PAVs analysis^14^. Our team successfully developed HUPAN, an automatic human pangenomic analytical pipeline^11^. With HUPAN, we de novo assembled 185 pairs (370 samples) WGS data from gastric cancer and normal gastric epithelium. We used WGS data from SGDP and 90 Hans as control and identified the PAVs landscape of the gastric cancer population. Four distributed genes *ACOT1*, *GSTM1*, *SIGLEC14*, and *UGT2B17* showed extremely high frequencies of absence in the gastric cancer population. Compared to datasets from SGDP and 90 Hans, *ACOT1* showed a high frequency of absence in the Chinese Han population. *GSTM1* and *SIGLEC14* genes revealed a high frequency of absence in East Asian, while *UGT2B17* gene was highly absent in all Asian individuals. These genes were previously reported as null or deletion polymorphism in some disease conditions by traditional genomics studies based on the human reference genome^15–18^. Genes *UGT2B17* and *GSTM1* are both enriched in the chemical carcinogenic signaling pathway. This finding partially explained the high incidence of gastric cancer in East Asian, especially in Chinese Hans. The functional deletion of *SIGLEC14* could result in insufficient secretion of tumor necrosis factor α, which is a fundamental cytokine in response to microbial infection^17^. Hitherto, the correlation of *SIGLEC14* null variation with cancer has not been reported yet. *ACOT1* was identified as a shared deletion variation between human and archaic hominin genomes and both have higher frequencies of deletion in the Asian population than that in other populations^16^. Although one report indicated that the expression of *ACOT1* is related to a poor prognosis of gastric cancer^19^, the exact biological function of *ACOT1* in gastric carcinogenesis needs to be explored.

Although the HUPAN analysis requires computing resources, it does help researchers discover genes on non-reference genome sequences. In the current study, we successfully characterized a gene *GC0643*. This gene was confirmed by long-read sequencing from several Asian and non-Asian individuals. Since the non-reference sequences usually reflect structure variations in the human genome with potential functional significance^20^, we added SV analysis by the traditional genomic method. We recognized two SV breakpoints at 9q34.2, the *GC0643* locus. It suggested that the SV breakpoints should be further explored in the future genomic study. We also aligned the protein-encoding sequence of GC0643 with proteomic data from gastric cancer and hit the peptide of GC0643 in two gastric cancer cases. The mRNA expression of *GC0643* was clearly localized in cytoplasm of gastric epithelium by mRNA probe in situ hybridization. Importantly, overexpression of *GC0643* gene in cancer cells revealed stronger tumor suppressor function. By functional verification, the upregulated genes in *GC0643* overexpression group are enriched in lymphocyte activation and detoxification pathways, while the downregulated genes of *GC0643* overexpression are enriched in wound healing, regulating cytokine-mediated signaling, and cell division pathways. A serial of validation evidence supports that gene *GC0643* is a tumor suppressor gene. The absence or inactivation of *GC0643* gene is closely related to gastric carcinogenesis. The gene has been approved by NCBI database (https://www.ncbi.nlm.nih.gov/nuccore/MW194843.1; GenBank: MW194843.1). Although we call *GC0643* a new gene, it must be pointed out that it has no homology to proteins in the NR database. It locates inside a large intron of *FAM163B*, and has a 22 bp small intron without 5’-UTR sequence in transcriptome data. More studies should be done to further understand *GC0643*. Moreover, the absence of *GC0643* is not only in somatic level, but also in germline level based on data analysis from SGDP and 90 Hans. It suggests that PAVs of distributed genes in a specific population may have a potential pathogenic association. Our current functional studies have been done in cellular level. Further studies are undergoing on different animal models.

In conclusion, we have developed a new strategy for cancer genomics study by combining the human reference genome and non-reference sequences. From 185 paired gastric cancer and normal mucosa, we constructed GCPAN. Based on GCPAN, we characterized gene PAVs of human gastric cancer. Genes *ACOT1, GSTM1, SIGLEC14*, and *UGT2B17* are highly absent in Chinese Hans, even in the Asian population, compared to the non-Asian healthy population, suggesting a potential association for the high incidence of gastric cancer in the Asian population. In addition, we have predicted a group of genes. Among them, *GC0643* is a tumor suppressor gene for gastric cancer.

## Methods

### Biospecimen collection

Patient cohorts, samples, and ethics: all subjects were diagnosed with gastric cancer and underwent gastrostomy in Ruijin hospital, Shanghai Jiao Tong University School of Medicine (*n* = 140) and Shanghai Cancer Center, Shanghai Medical College, Fudan University (*n* = 50). No neoadjuvant or adjuvant chemotherapy and radiotherapy were administered before surgery. Cancer tissues and non-cancerous mucosae more than 5 cm away from the main cancer were collected within 30 min after surgery and immediately frozen in liquid nitrogen and stored at −80 °C until DNA and RNA extraction. All enrolled cancer tissues disclosed 70% pure tumor cells. Written informed consent was obtained from each patient. The study was approved by the institutional review board. The study was approved by the institutional review board of Ruijin Hospital, Shanghai Jiao Tong University School of Medicine.

### Whole-genome sequencing

Genomic DNA was extracted from the tissues using QIAamp DNA kit (Qiagen, Germany). The sequencing libraries were constructed using TruSeq DNA LT Sample Preparation Kit V2 (Illumina) in accordance with the manufacturer’s protocol. After purification, quantification, and validation, the DNA libraries were sequenced on Illumina Sequencing System (HiSeq X10) according to the manufacturer’s paired-end (2 × 150 bp) protocol. Five paired samples were removed due to genotype mismatch between primary tumor tissues and matched gastric mucosae, resulting in 185 paired samples in the final analysis. Raw Illumina reads WGS were processed for quality control using FastQC (http://www.bioinformatics.babraham.ac.uk/projects/fastqc/).

### De novo assembly of genome sequences

SGA (version 0.10.15) was used to assemble raw reads into contigs for each sample^21^. De novo assembly of 185 primary tumor tissues was conducted by SGA. All the assembled results were accessed by QUAST (version 4.5) to get the total length of unaligned contigs and misassembled contigs^22^. The contigs longer than 500 bps were kept to subsequent analysis.

### Identification and annotation of non-reference sequences

The non-reference sequences (including fully unaligned contigs and partially unaligned contigs) were extracted from individual assembled genomes according to the HUPAN pipeline^11^. After removing redundancies and potential contaminations, a total length of 80.88 Mbp representing the non-reference genomic sequences was obtained. Protein-coding genes on non-reference sequences were predicted using MAKER (version 2.31.9)^23^ combining ab initio predictions, transcript expression, and protein evidence.

### Construction and annotation of GCPAN

All contigs longer than 500 bps were aligned to the GRCh38 reference genome by MUMmer package (v3.23)^24^ with default parameter. Contigs with 95% or more identity as well as covering 95% sequences’ lengths were considered as the reference genome sequences. We added the non-redundant non-reference sequences into GRCh38 primary sequences to construct the sequences of GCPAN. The annotation of GCPAN was performed by combining the annotation information of the reference genome from GENCODE (version 30)^25^ and the newly predicted novel genes on the non-reference sequences. For validating reads mapping ratio by GCPAN comparison to GRCh38, sequencing data from the SGDP^12^ were used as external control.

### Gene presence-absence variations (PAVs) analysis

The raw reads from each sample were aligned to GCPAN sequences using Bowtie2 (version 2.3.3.1). For each protein-coding gene, only the transcript with the longest open reading frame was selected as the representative transcript. The percentage of CDS region covered by mapped reads was calculated by SAMtools (version 1.3)^26^. A gene with more than 80% of CDS region coverage was considered a gene presence; otherwise, it was considered a gene absence. The genes present in all individuals were defined as core genes, and the rest genes, which were absent in at least one individual, were defined as distributed genes. Due to the deficiency of chromosome Y in all female individuals, the genes located in chromosome Y were treated as core genes if they were presented in all male individuals. For distributed genes located on the human reference genome, we conducted functional analysis by Metascape (version 3.5)^27^.

### Positioning predicted genes on chromosomes

We used the third-generation sequencing datasets from NCBI short-read archive under the studies PRJNA301527, PRJA339722, PRJNA530217, and PRJNA551670 to locate the non-reference genes to their corresponding chromosome positions. The dataset of PRJNA301527 was from a Chinese genome study. The other datasets were from genomes with African ancestry. We aligned sequences of 14 predicted genes to the third-generation sequencing contigs. If the global identity of a predicted gene is greater than 80% of its protein-coding regions, this gene is considered a gene supported by the third-generation sequencing data. We included upstream and downstream regions (3000 bp each) on the third-generation sequencing contigs, which were used as anchor sequences, and then aligned the two anchor sequences to the human reference genome. The anchor sequence was considered aligned if the alignment had a length greater than 1500 bps with a percentage of sequence identity greater than 80%. If the region between two anchor sequence positions on the reference genome was less than twice the predicted gene length, we consider this region as the chromosomal position of the gene.

### Validation of predicted genes with proteomics data

To validate the expression of predicted genes at the proteome level, the MS/MS dataset of 80 diffuse gastric cancer samples was obtained from CPTAC^28^, and searched with X!Tandem (version 2017.2.14)^29^ against the human protein sequence database (GENECODE v30). The following parameters were set for static modifications in database searching: iTRAQ to lysine, N-terminus, and carbamidomethylation, while oxidation to methionine and deamidation to asparagine and glutamine were used as variable modifications. The precursor mass tolerance was set to 10 ppm and the fragment mass tolerance was set to 0.02 Da. Semi-tryptic cleavage was allowed with up to two missed cleavages permitted. For predicted gene identification, 0.1% separated FDR at PSM level was used to call candidate peptides. There is no obvious bias between the predicted genes and the wild-type peptides in the searching score distributions^30^. Then, the intensities of all four iTRAQ reporter ions were extracted using MASIC software (version 3.0.7235)^31^. The hitting peptides meant that could be occurred in at least two samples. All passed peptides were then aligned to the human protein sequence database to check no alignment to the referenced peptides by BLASTP^32^.

### Comparison of PAVs and SVs by GRCh38-based methods

The GATK Best-Practices pipeline (https://software.broadinstitute.org/gatk/best-practices/) was performed to mark PCR duplications and apply base quality score recalibration. Three tools were used for structural variation detection with default parameters: Delly^33^ (version 0.8.7), Manta^34^ (version 1.6.0), SvABA^35^ (version 1.1.3). The SVs of each sample derived from the above SV callers were merged using SURVIVOR^36^ with the following parameters: “SURVIVOR merge name 1000 2 1 1 0 30”. SURVIVOR filtered variants were detected by at least two tools according to breakpoint positions, SV types, and SV lengths. Merged deletion SVs (DEL-SVs) and homozygous DEL-SVs were respectively selected to calculate CDS region coverage and determine genes’ PAVs following the same strategy of PAV analysis. We extracted insertion SVs (INS-SVs) to compare with unaligned sequences in GCPAN analysis. The insertion sequences were firstly masked by TRF (version 4.09.1)^37^ with the command: “trf 2 7 7 80 10 50 500 -f -h -m”. The INS-SVs with more than half repeat sequences of the SV length were filtered out. The INS-SVs sequences were mapped to GCPAN by BLASTN. The best hit of each sequence was determined according to the length and identity percentage of the aligned region.

### Identification of distributed genes related to gastric cancer

The absence frequencies of distributed genes in gastric cancer datasets and SGDP data were compared. The significance of the difference of frequencies for each gene absence in two groups was calculated using Fisher’s exact test. The *P* values were further corrected by FDR. Fisher’s exact test is used to calculate the correlations between gene PAVs with clinical phenotypes of gender, age, Borrmann classification, Lauren classification, tumor location, histological grade, tumor diameter, Hp, and EBV infection.

### Transcriptome sequencing

Total RNAs were extracted from cancer tissues in 87 patients diagnosed with gastric cancer using the TRIzol solution (Invitrogen, Carlsbad, CA, USA), according to the manufacturer’s protocols. RNA sequencing libraries were constructed using TruSeq RNA Sample Preparation Kit V2 (Illumina) following the manufacturer’s protocol. RNA concentration was measured by Nanodrop and the quality was measured by Agarose and Agilent 2100. Following purification, the mRNA was fragmented into small pieces using divalent cations under elevated temperature. The cleaved RNA fragments were copied into first-strand cDNA using reverse transcriptase and random primers. The products were purified and enriched with PCR (15-cycle) to create the final cDNA library, and sequenced on Illumina Sequencing System (HiSeq2000) following the manufacturer’s standard workflow. RNA-seq reads were trimmed by trimmomatic^38^ (version 0.32) with the parameters “ILLUMINACLIP:TruSeq3-PE.fa:2:30:10 LEADING:3 TRAILING:3 HEADCROP:13 SLIDINGWINDOW:4:15 MINLEN:36” and mapped to reference GRCh38 by HISAT2^39^ (version 2.1.0) with default parameters. The reads uniquely mapped to exons of genes in GENCODE (version 30) and novel genes predicted were measured to quantify the transcript levels of genes by featureCounts^40^ (version 2.0.0) with parameters “-p -O -t exon -g gene id”. In the normalization of gene expression, FPKM values were used for quantifying the transcript levels of genes.

### RNAScope examination

The target probes to *GC0643* mRNA (BA-Hs-GC000643-3zz-st-C1, #1051091-C1) are customized by Advanced Cell Diagnostics Inc (Hayward, CA, USA). The 5-μm thickness tissue sections of human gastric mucosa were deparaffinized in xylene, followed by dehydration in a series of ethanol. Tissue sections were then incubated in citrate buffer (10 nM, pH 6) maintained at a boiling temperature for 15 min, rinsed in deionized water, and immediately treated with 10 μg/mL protease. Hybridization with *GC0643* target probes, preamplifier, amplifier, label probe, and chromogenic fast red detection was performed as the manufacturer’s protocol. To ensure interpretable results, we used *PPIB* (Hs-PPIB-3ZZ, #701030, NM_000942.4) an endogenous housekeeping gene as a positive control. This ultrasensitive RNA in situ hybridization technology allows detecting single-molecule mRNA^41^. Images were acquired using a Zeiss AxiopCam ICc 5 microscope (Carl Zeiss, Germany). The single mRNA transcript appears as red dot at brightfield microscope at ×40–100 magnification.

### Cell culture and construction of eukaryotic expressing vector

Human gastric cancer cells HGC27 and NCI-N87 were grown in RPMI-1640 medium supplemented with 10% fetal bovine serum (FBS; Gibco, Grand Island, NY). *GC0643* overexpression vectors and shRNA against *GC0643* were constructed by Genomeditech (Shanghai, China). Stable transfected cell lines that stably expressed *GC0643* were established by retroviral infection. Puromycin (2 μg/mL, Genomeditech, Shanghai, China) was used to select stable cells for 2 weeks. For knockdown of *GC0643*, shRNAs were used. Non-targeting control shRNA was used as a negative control. Stably transfected cells were then validated by mRNA and protein (HRP-conjugated mouse anti-DDDDK-Tag, 1:5000, AE024, ABclonal, China) expression analysis. HRP-conjugated GAPDH monoclonal antibody (1:5000, HRP-60004, Proteintech, USA) was used as control.

### Cell proliferation assay

For CCK8 analysis, cells are plated into 96-well plates by quadruplicate at a density of 3000 cells per well. Cell proliferation was measured at 72 h by Cell Counting Kit-8 kit (DOJINDO CK04, Kumamoto, Japan). The absorbance was measured by spectrophotometer (BioTek, Vermont, USA) at 450 nm. For colony formation assay, 1000 cells were seeded in a 6-well plate. Colonies were stained with 0.5% crystal violet after 10-day cultivation. For EdU cell proliferation assay, HGC27 or NCI-N87 cells (3 × 10^5^/well) were planted in the 24-well plate for 24 h, and then added 10 μM EdU (C0081S, Beyotime, China) reagent for 2 h incubation. The cells were fixed with 4% paraformaldehyde (P0099, Beyotime, China) for 15 min, washed three times with 3% BSA (ST023, Beyotime, China), and permeated by the Immunostaining Washing Solution (P0106, Beyotime, China) for 15 min. Then cells were stained by Click Reaction Solution (Click Reaction Buffer 430 μL, CuSO4 20 μL, Azide 555 1 μL, and Click Additive Solution 50 μL) (C0081S, Beyotime, China) for 30 min and Hoechst 33342 (1:1,000, Beyotime, China) for 10 min. After nuclear staining, the photos were captured using an inverted fluorescence microscope (Nikon TS2R-FL, Nikon, Japan) on randomly selected six fields. The proliferating cell nuclei incorporated with EdU were marked by red fluorescence and all cell nuclei were marked by blue fluorescence. The proportion of proliferating cell nuclei to all cell nuclei was calculated to reflect the proliferating cell rate.

### Cell migration and invasion assay

In the wound healing assay, cells were plated into 24-well plates in an equal count for the experimental group and control (5 × 10^5^/well). After incubating for 24 h, cell monolayers were scratched using a pipette tip. The cells were washed with the culture medium. Migration was photographed under a microscope at 0, 8, and 24 h after the scratch. The distances of the wound in the microscopic pictures were measured. For cell migration and invasion assay, the transwell chambers (Corning, Lowell, MA, USA) were coated with or without matrigel (BD Biosciences, Bedford, MA). Cells (3 × 10^4^/well) were added to the upper chamber and cultured for 48 h. Cells were then stained with 0.5% crystal violet for 30 min, and non-migrating or non-invading cells from the upper surface of the chambers were softly removed by cotton swabs. Permeating cells were counted under the inverted microscope in five random fields.

### Cell apoptosis and cell cycle assay

For apoptosis rate analysis, cells are washed with PBS and incubated with PE and FITC using Cell Cycle and Apoptosis Kit (40301ES50, Yeasen, Shanghai, China) according to the manufacturer’s protocol. For cell cycle assay, cells are harvested and fixed with 75% ethanol at 4 °C overnight. Then cells are washed with cold PBS and stained with propidium iodide for 30 min in dark. Apoptosis rate or cell cycle distribution was analyzed by FACS (Becton-Dickinson, Franklin Lakes, NJ, USA), and analyzed using FlowJo 7.6.1 software (FlowJo, RRID: SCR_008520).

### Statistics and reproducibility

All statistical tests were performed in R (version 4.0.2) and GraphPad Prism (version 8). The nonparametric Mann–Whitney *U* test, Fisher’s exact test, and Student’s *t*-test were used to compare between groups. We also used the log-rank test to perform survival analysis. Wilcoxon rank sum tests and Fisher’s exact tests were conducted between distributed genes and clinical phenotypes. For in vitro experiments, at least three independent replications were performed, and representative photos were shown. All statistical tests were two-sided except for special explanations. No data were excluded from the analyses.

### Reporting summary

Further information on research design is available in the Nature Research Reporting Summary linked to this article.

## Supplementary information

Supplementary Information

Reporting Summary

## Data Availability

The raw sequencing data of genomic and transcriptomic sequencing reported in this paper have been deposited in the Genome Sequence Archive in National Genomics Data Center, China National Center for Bioinformation (GSA-Human) HRA002344 for normal gastric mucosa and HRA002333 for gastric cancer. The raw sequencing data are available under restricted access due to data privacy laws. Readers can get access to data by sending requests to corresponding authors. Data will be available within a week once the access has been granted. The processed data and result files are available on the website http://cgm.sjtu.edu.cn/cpan/GCPAN.html. The 90 Han Chinese data [http://gigadb.org/dataset/100302] and SGDP data under accession number PRJEB9586 for supporting the findings of this study are open accessible. The proteomics data for gastric cancer (PDC000214) from the CPTAC project [https://pdc.cancer.gov/pdc/browse/filters/primary_site:Stomach] and the long-read sequencing data of humans (PRJNA301527, PRJA339722, PRJNA530217, and PRJNA551670) [https://www.ncbi.nlm.nih.gov/sra] for positioning the non-reference genes to corresponding chromosomes are open accessible. Source Data are provided with this paper.

## Source data

Source Data

## Code availability

The codes for this study are available at https://github.com/SJTU-CGM/CPAN.
