# Supplementary material for: Pangenomic analysis of Chinese gastric cancer
Source: Nat Commun. 2022 Sep 15;13:5412. doi: 10.1038/s41467-022-33073-7 (PMC9477819; doi:10.1038/s41467-022-33073-7)
Supplement: Supplementary file 1 — Supplementary Information [file 41467_2022_33073_MOESM1_ESM.pdf]

Supplementary information

Pangenomic analysis of Chinese gastric cancer

Yingyan Yu[1#*], Zhen Zhang[2#], Xiaorui Dong[3,#], Ruixin Yang[1#], Zhongqu Duan[3,4, #], Zhen Xiang[1], Jun Li[1], Guichao Li[2], Fazhe Yan[3], Hongzhang Xue[3], Du Jiao[3], Jinyuan Lu[3], Huimin Lu[3], Wenmin Zhang[3], Yangzhen Wei[3], Shiyu Fan[3], Jing Li[3], Jingya Jia[3], Jun Zhang[5], Jun Ji[1], Pixu Liu[6], Hui Lu[3,4], Hongyu Zhao[4], Hai Fang[7], Saijuan Chen[7], Chaochun Wei[3, 4,*], Hongzhuan Chen[8, 9*], Zhenggang Zhu[1*]

Supplementary figures

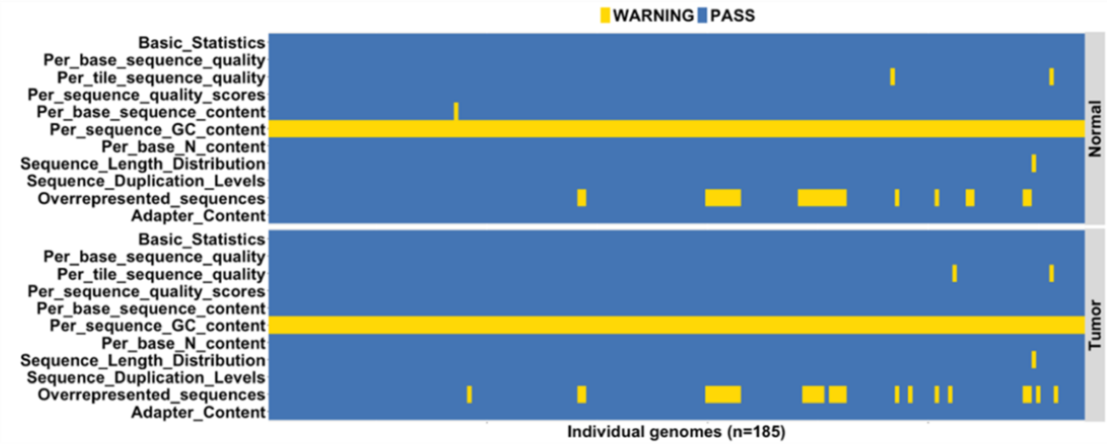

Supplementary Fig. 1 Summary of sequencing quality in current WGS study. The data are across 185 gastric tumor and normal mucosa using FastQC.

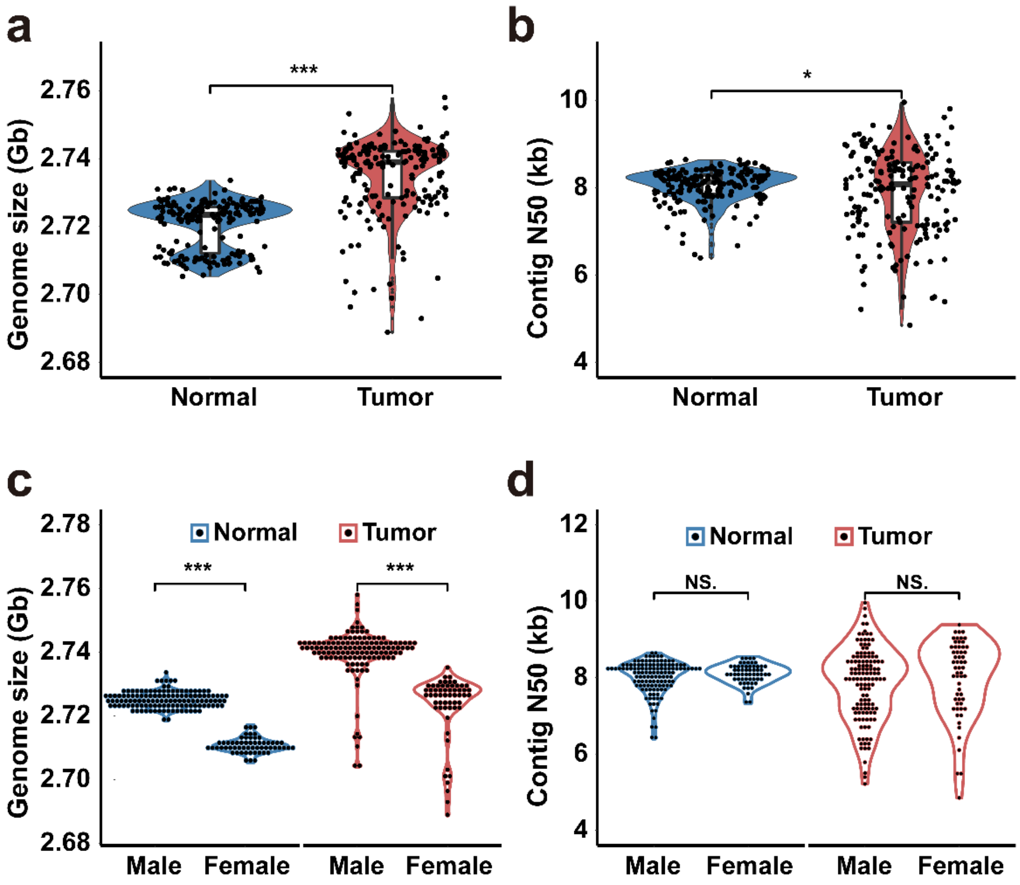

Supplementary Fig. 2 *De novo* assembly of genome sequences by contigs (≥ 500 bp) across 185 paired (370 samples) of normal mucosa and tumor tissues. **a** Comparison of genome sizes between tumor tissue and matched normal mucosa ($P <$ 2.2e-16). **b** Comparison of N50 sizes between tumor tissue and matched normal mucosa (P=0.025). The center lines of box plots in panel a and b stand for median values, hinges for the first and third quartiles and the whiskers for maxima and minima within 1.5 times of the interquartile range. **c** Comparison of genome sizes between male (n = 127) and female (n = 58) individuals (Normal: $P <$ 2.2e-16; Tumor: $P <$ 2.2e-16). **d** Comparison of N50 sizes between male and female individuals. The comparison between primary tumor tissue and matched normal mucosa was tested by paired t-test, and the comparison between male and female individuals was tested by Wilcox test. Note: *, $P \leq 0.05$; ***, $P \leq 0.001$; NS., not significant; Normal, matched normal mucosae; Tumor, tumor tissues.

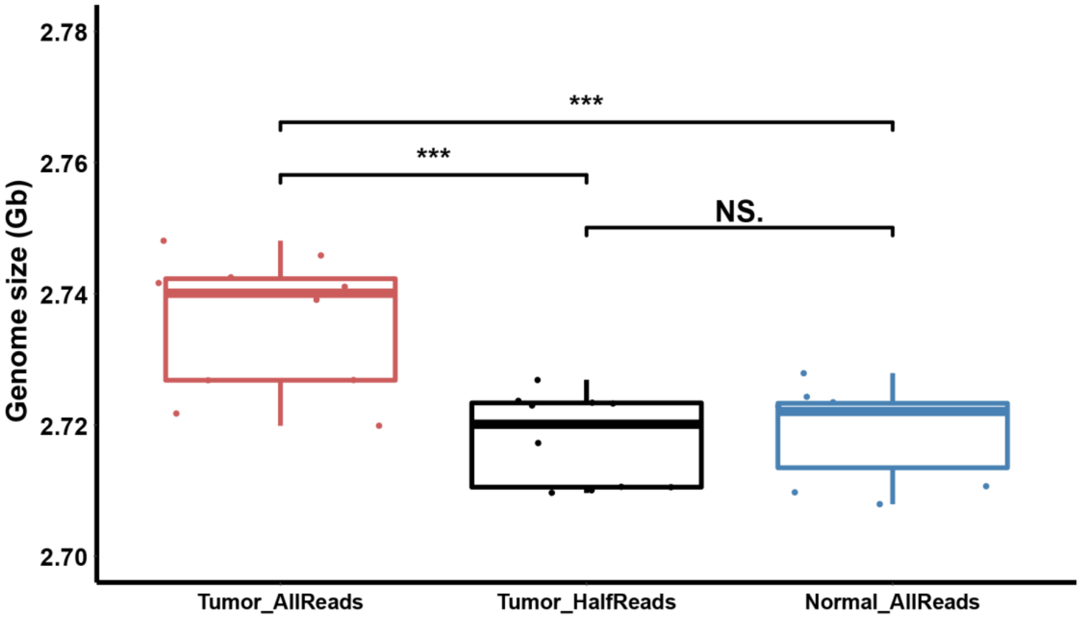

Supplementary Fig. 3 Evaluation of the impact of sequencing depth on assembled results using 10 randomly selected individual genomes. The center lines of the box plots stand for median values, with the hinges for the first and third quartiles and the whiskers for the maxima and minima within 1.5 times of the interquartile range. The comparisons were tested by two-sided paired t-test (Tumor_AllReads vs. Tumor_HalfReads: P = 3.6e-07; Tumor_AllReads vs. Normal_AllReads: P = 5.2e-05). Note: ***, $P \leq 0.001$; NS., not significant; Tumor_AllReads, *de novo* assembly was performed using all reads of tumor tissues; Tumor_HalfReads, *de novo* assembly was performed using half of reads from tumor tissues; Normal_AllReads, *de novo* assembly was performed using all reads of matched normal mucosae.

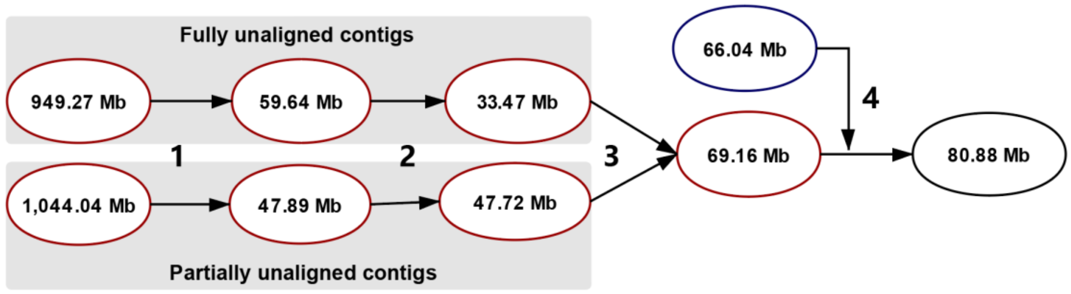

Supplementary Fig. 4 The procedure of obtaining non-reference sequences. 1, Removing redundant sequences; 2, Removing potential contaminations; 3, Removing the redundant sequences between fully and partially unaligned contigs; and 4, Removing the redundant sequences between matched normal mucosa (66.04 Mb) and primary tumor tissues (69.16Mb) to generate 80.88Mb non-redundant novel sequences.

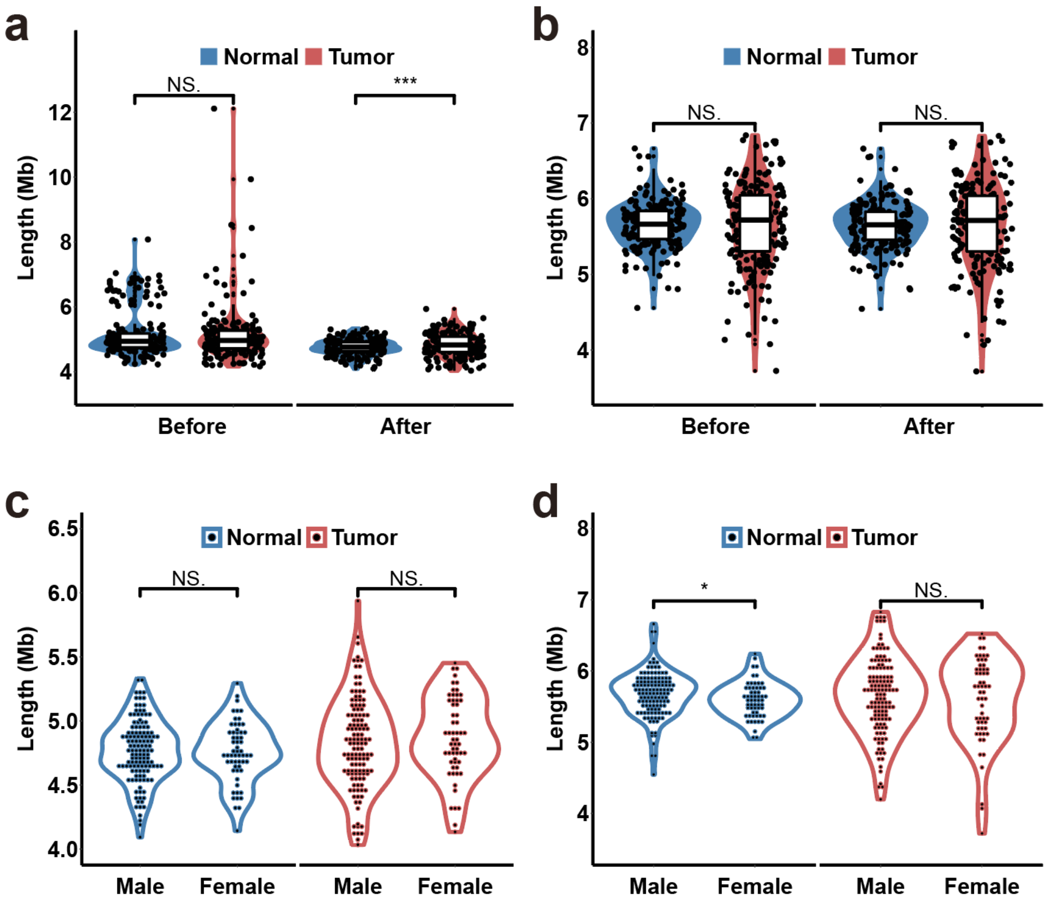

Supplementary Fig. 5 The length distributions of non-reference sequences. **a** Fully unaligned contigs of n=185 individual genomes compared to GRCh38 primary assembly sequences before and after removing potential contaminations (After: *P*=9.3e-6). **b** Partially unaligned contigs of n=185 individual genomes compared to GRCh38 primary assembly sequences before and after removing potential contaminations. The center lines in box plots in panel a and b are for median, hinges for the first and third quartiles and the whiskers for the maxima and minima within 1.5 times the interquartile range. **c** Comparison of the length of fully unaligned contigs per genome between male and female individuals. **d** Comparisons of the length of partially unaligned contigs per genome between male and female individuals (Normal: *P*=0.021). The comparison between primary tumor tissue and matched normal mucosa was tested by paired t-test, and the comparison between male and female individuals was tested by Wilcox test. Note: *, $P \leq 0.05$; NS., not significantly; Normal, matched normal mucosa; Tumor, tumor tissue.

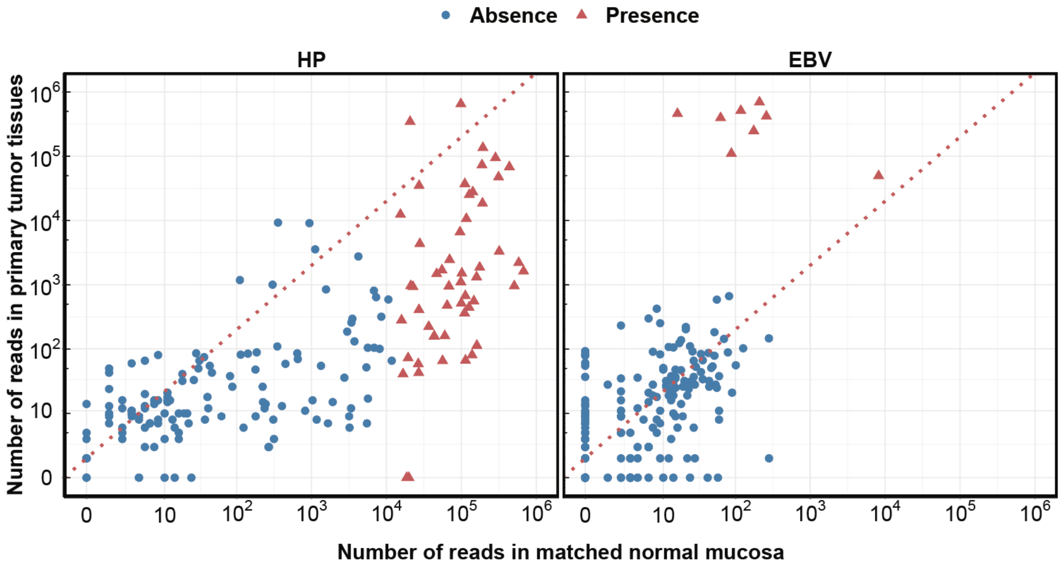

Supplementary Fig. 6 The alignment of fully unaligned sequences to the reference genome sequences of HP and EBV across 185 individuals. All reads that could not be mapped to the constructed GCPAN were aligned to the reference genome sequences of HP and EBV, respectively. The reads with CIGAR flag as “150M” in the alignment file were counted. Alternatively, all the unaligned contigs compared to the GRCh38 primary assembly sequences were aligned to the reference genome sequences of HP and EBV, respectively. For a sample, if at least one contig could be aligned to the reference genome sequences of HP or EBV, we considered it (HP or EBV) as presence in this sample. Otherwise, the HP or EBV was considered as absence in the sample. The red line ($\log_{10}y=\log_{10}2x$) denotes the average of sequencing depth of tumor tissue is twice as that of matched normal mucosa.

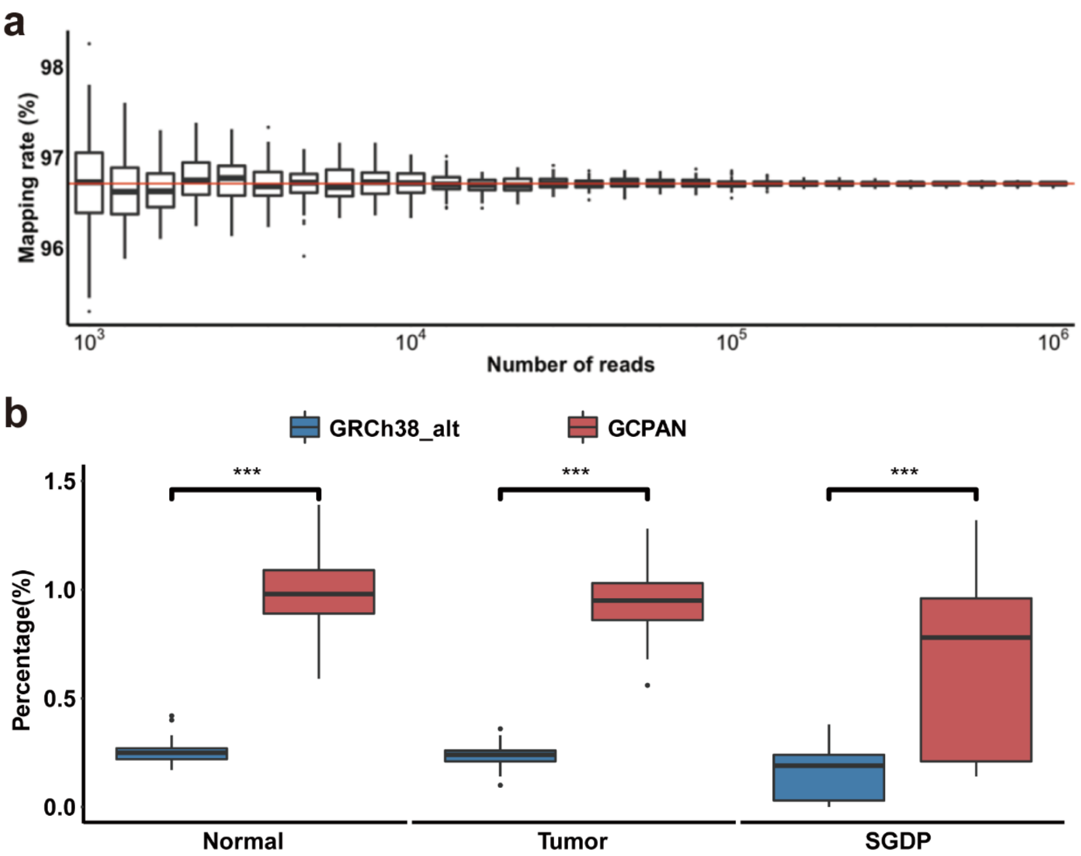

Supplementary Fig. 7 Comparison of read mapping rates between the human reference genome and GCPAN. **a** We randomly selected a number of reads (from 10^3 to 10^6) from sample NA12878. The mapping rate of one million reads is close to the overall mapping rate of using all reads. The red line is the overall mapping rate using all reads. **b** Comparison of the percentages of aligned reads to different reference genomes (Normal: P<2.2e-16; Tumor: P<2.2e-16; SGDP: P<2.2e-16). Y-axis represents the percentage of reads aligned to the alternative sequences of the human reference genome (GRCh38_alt) and GCPAN. For comparison, the increased percentages of aligned reads were obtained for our dataset (n=185) and the SGDP dataset (n=263) using GCPAN as the base line. The center lines of box plots in panel a and b stand for median values, hinges for the first and third quartiles and the whiskers for maxima and minima within 1.5 times of the interquartile range. The significance of difference was determined by Welch's two-sample paired t-test. Note: ***, $P \leq 0.001$; Normal, matched normal mucosa; Tumor, tumor tissue; SGDP, Simons Genome Diversity Project.

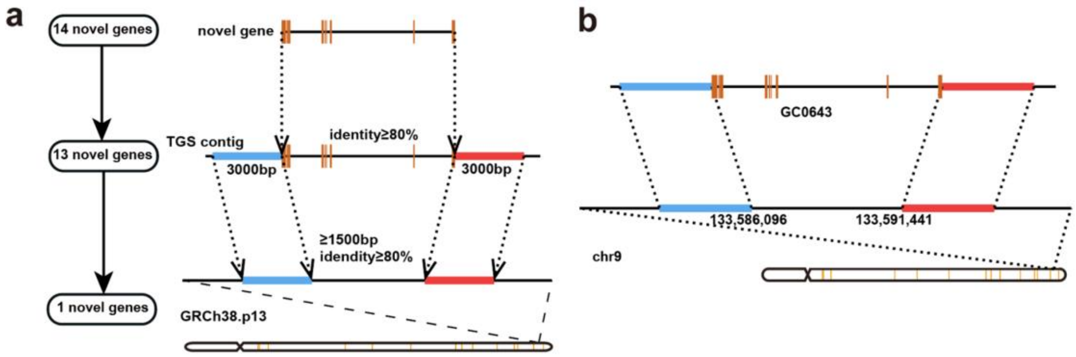

Supplementary Fig. 8 Positioning predicted genes on chromosomes using the third generation sequencing data. **a** Among 14 predicted new genes, 13 can be aligned to at least one publicly available read generated by the third generation sequencing technology and one of them was positioned on chromosome. **b** The figure shows the predicted gene *GC0643* that can be located on chromosome 9q34.2

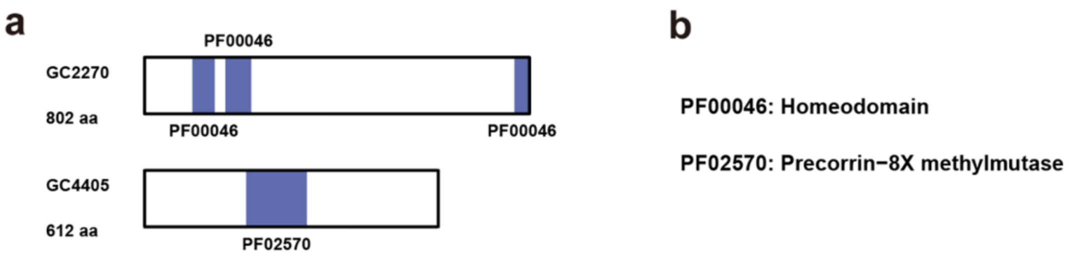

Supplementary Fig. 9 Potential functional domains for 14 predicted genes. The sequences of 2 (14.29%) predicted genes could be hit at least one functional domain in Pfam database using InterProScan (Version 5.39-77). **a** The gene names and sketch maps of 2 genes *GC2270* (distributed gene) and *GC4405* (core gene) with aligned functional domains (blue boxes). **b** The domain names and their functions are listed.

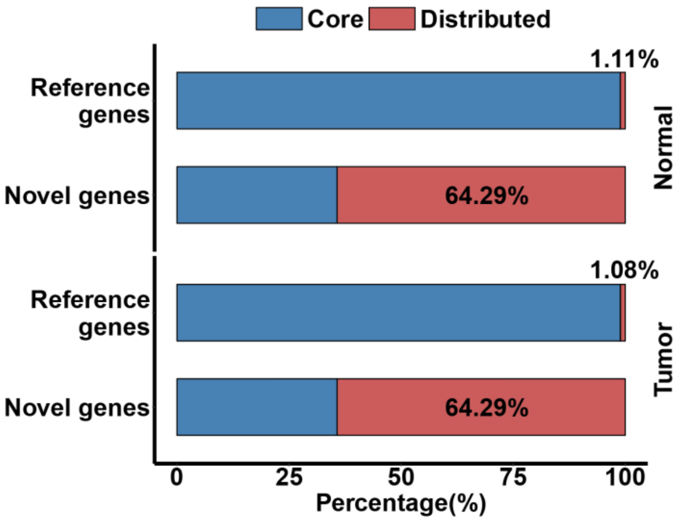

Supplementary Fig. 10 Comparison of the proportion differences of core genes and distributed genes in GRCh38 and novel predicted genes in the tumor and normal samples. Note: Normal, matched normal mucosae; Tumor, tumor tissues.

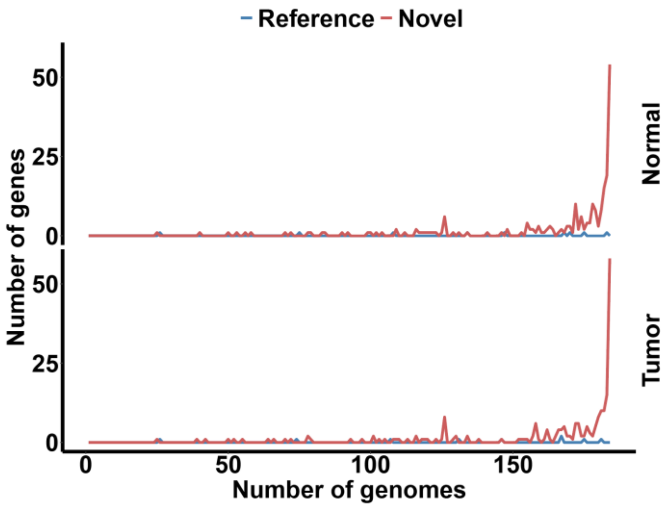

Supplementary Fig. 11 The numbers of distributed genes vs. the numbers of individual genomes containing them. The majority of distributed genes of human reference genome were sporadically missed in a small number of samples, whereas the distributed genes of non-reference sequences evenly spread across all samples. Note: Normal, matched normal mucosae; Tumor, tumor tissues.

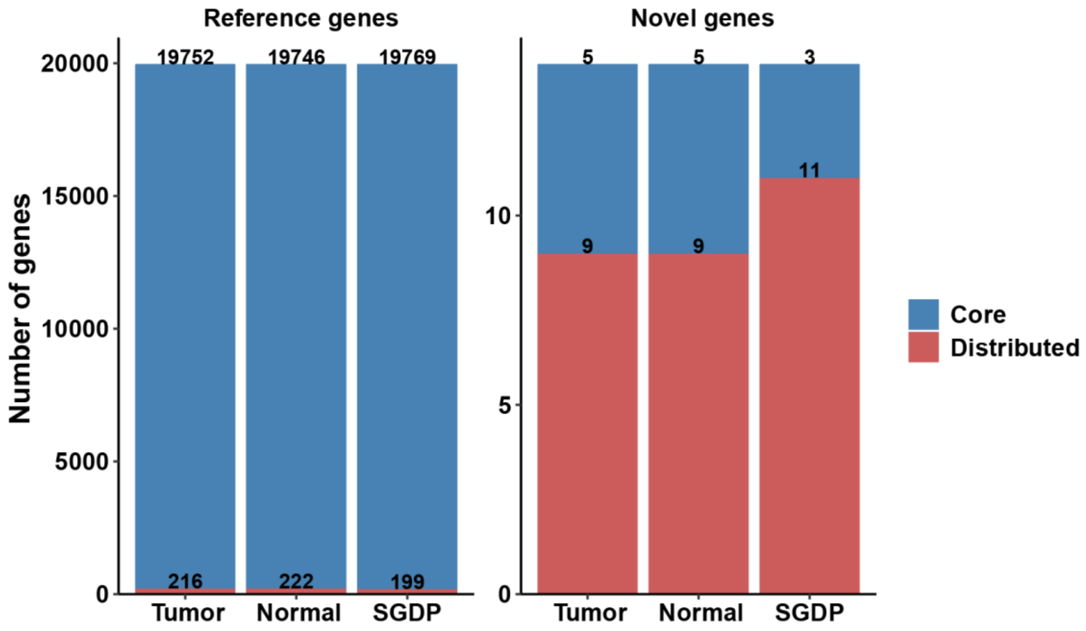

Supplementary Fig. 12 Comparison of the proportion differences of core genes and distributed genes in GRCh38 and predicted genes in different populations: Tumor, Normal and SGDP. Note: Normal, matched normal mucosae; Tumor, tumor tissues; SGDP, Simons Genome Diversity Project.

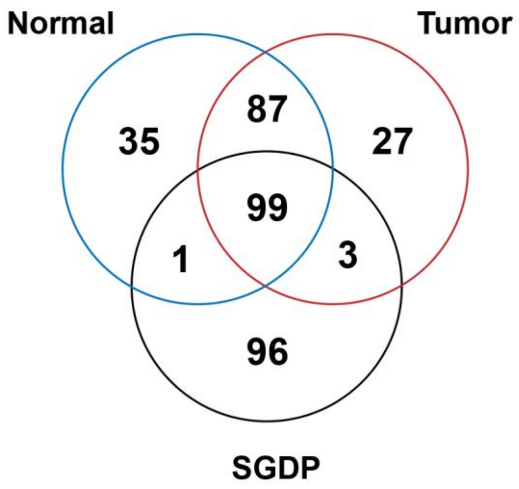

Supplementary Fig. 13 Overlapping of distributed genes between our groups and SGDP dataset for genes located on the human reference genome. Note: Normal, matched normal mucosae; Tumor, tumor tissues; SGDP, Simons Genome Diversity Project.

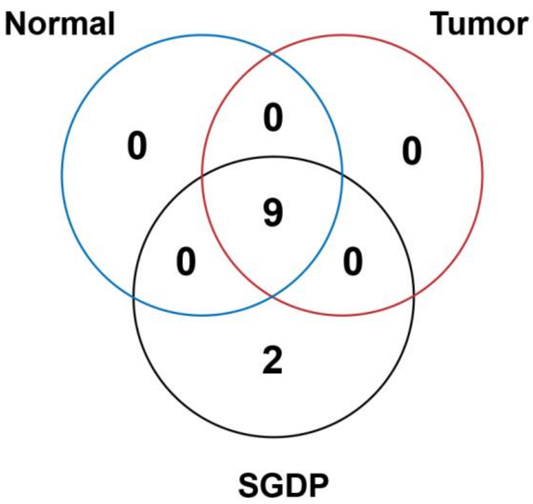

Supplementary Fig. 14 Overlapping of distributed genes between our groups and SGDP dataset for predicted genes. Note: Normal, matched normal mucosae; Tumor, tumor tissues; SGDP, Simons Genome Diversity Project.

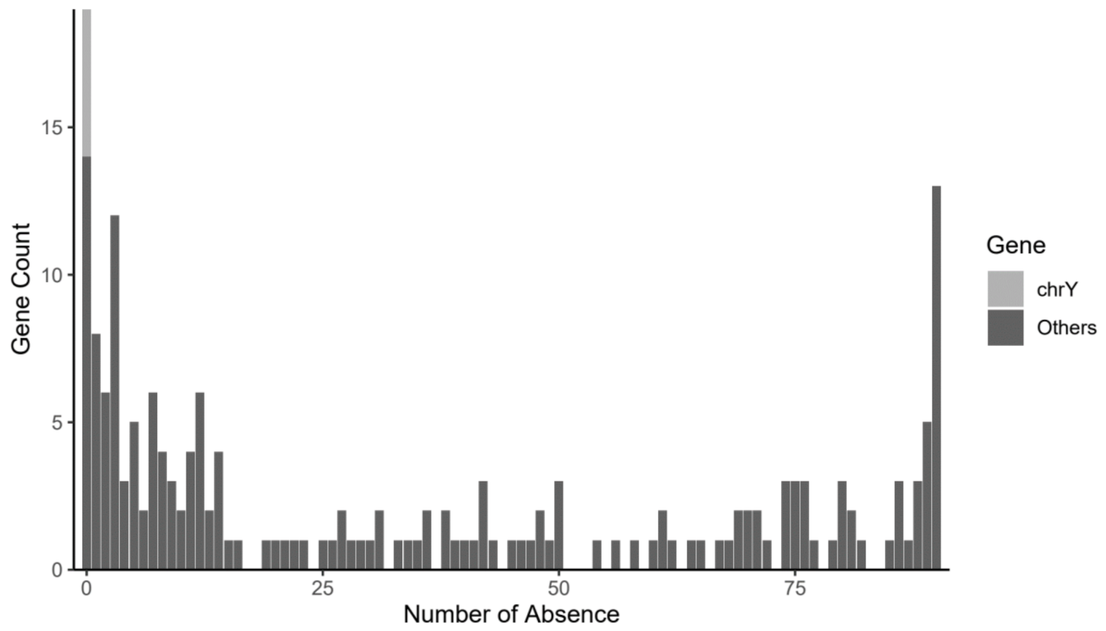

Supplementary Fig. 15 The number of genes vs the number of samples containing their absence variations in the 90 Han Chinese dataset. There are 19 genes showed no absence in all genomes of the 90 Han Chinese dataset. In total, 146 genes were absent in at least 5 genomes in the 90 Han Chinese data. This indicated that gene absence variations found in gastric cancer population were supported by the 90 Han Chinese dataset.

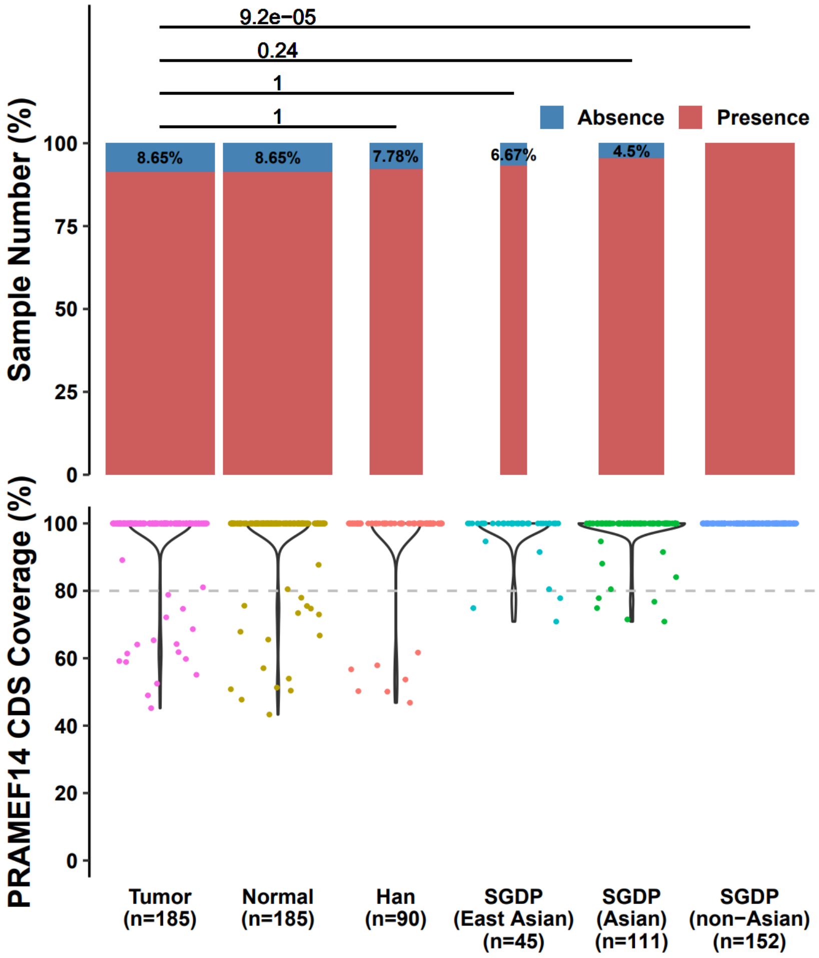

Supplementary Fig. 16 Comparison of gene absencce frequencies of *PRAMEF14* in different populations. The gene abasence frequencies in gastric cancer population and in East Asian population are significantly higher than that in SGDP non-Asian population. Fisher's exact test was used for statistical tests. Note: Normal, matched normal mucosae; Tumor, tumor tissues; SGDP, Simons Genome Diversity Project.

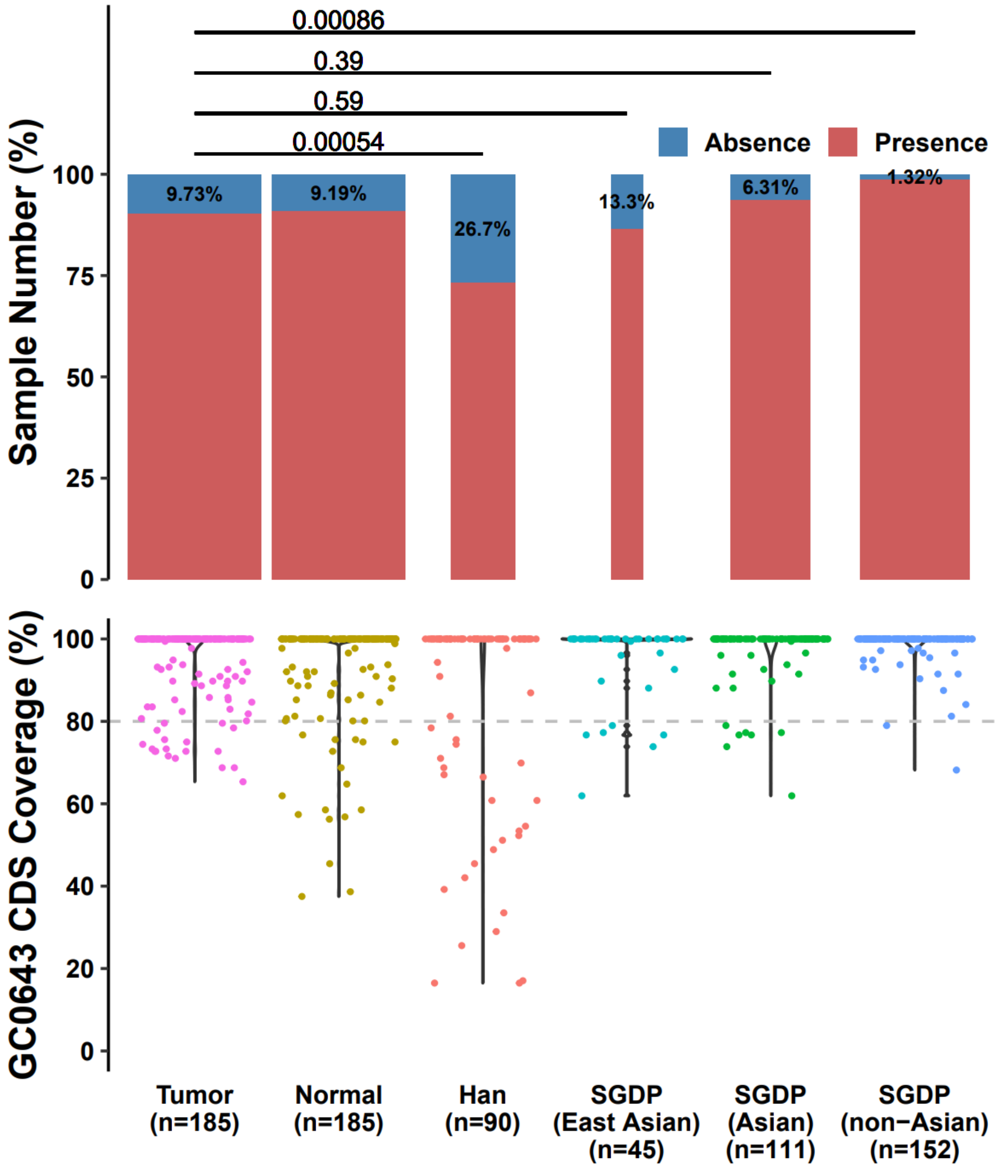

Supplementary Fig. 17 Comparison of gene absencce frequencies in different populations for gene *GC0643*. The gene abasence frequencies in gastric cancer population and in East Asian population are significantly higher than in SGDP non-Asian population. Fisher's exact test was used for the statistical tests. Note: Normal: matched normal mucosae; Tumor: primary tumor tissues; SGDP, Simons Genome Diversity Project.

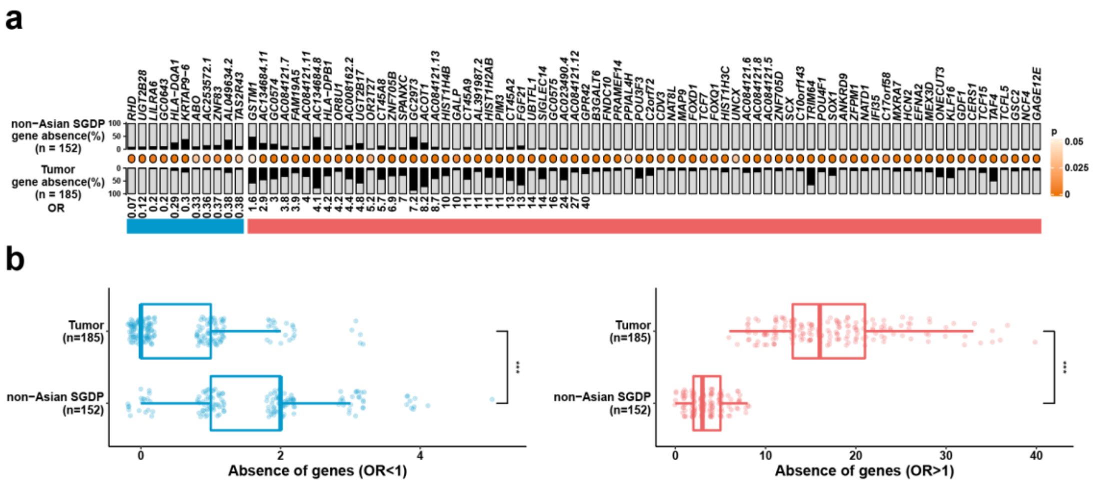

Supplementary Fig. 18 The absence variation features of distributed genes on GRCh38 between gastric cancer group and non-Asian population of SGDP groups. **a** The gene names, CDS coverage, *P* values (Fisher's exact test), and odds ratios of genes. Those genes without OR numbers marked are core genes in SGDP while distributed gene in gastric cancer group. **b** The comparison of gene numbers per sample in SGDP (n=152) and gastric cancer groups (n=185) (Wilcoxon test). The center lines of box plots in panel b stand for median values, hinges for the first and third quartiles and the whiskers for maxima and minima within 1.5 times of the interquartile range. Note: Tumor, tumor tissues; SGDP, Simons Genome Diversity Project.

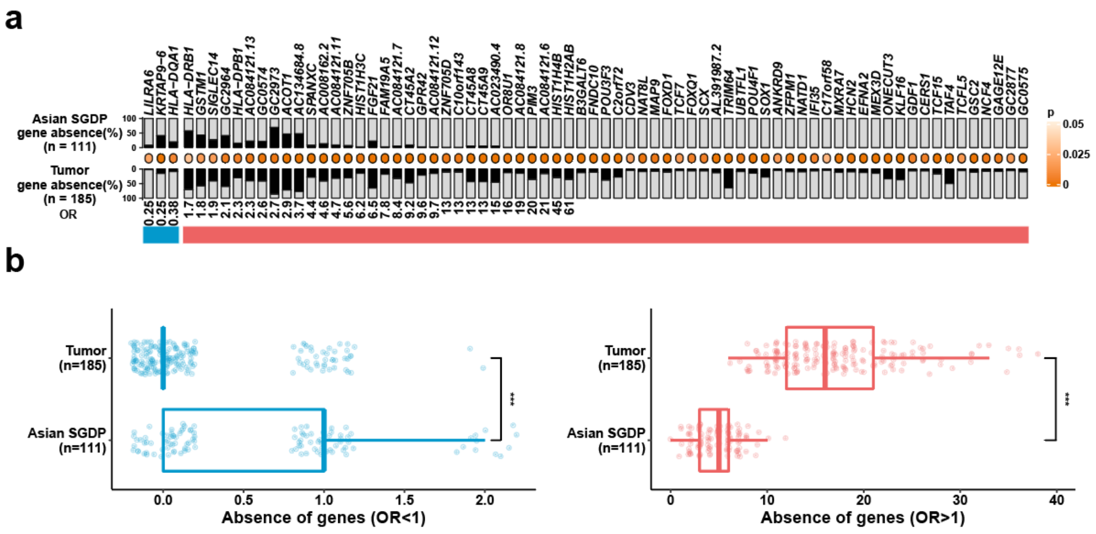

Supplementary Fig. 19 The absence variation features of distributed genes on GRCh38 between gastric cancer group and Asian population of SGDP groups. **a** The gene names, CDS coverage, *P* values (Fisher's exact test), and odds ratios of genes. Genes without OR numbers marked are core genes in SGDP while distributed in gastric cancer group. **b** The comparison of genes numbers per sample in SGDP (n=111) and gastric cancer groups (n=185) (Wilcoxon test). The center lines of box plots in b stand for median values, hinges for the first and third quartiles and the whiskers for maxima and minima within 1.5 times of the interquartile range. Note: ***, $P \leq 0.001$; Tumor, tumor tissues; SGDP, Simons Genome Diversity Project.

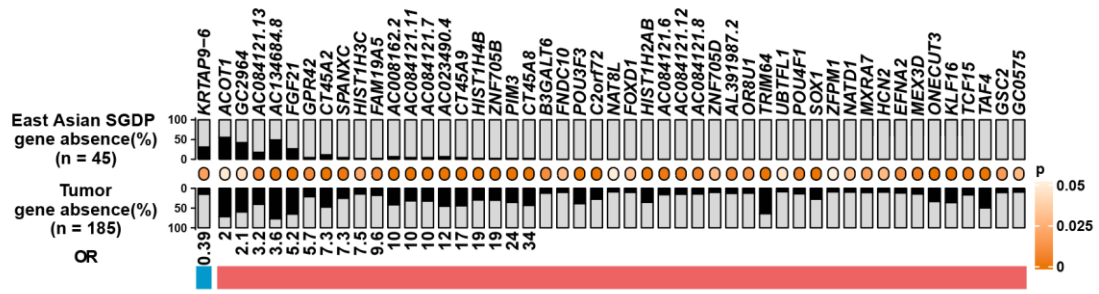

Supplementary Fig. 20 The absence variation features of distributed genes on GRCh38 between gastric cancer group and East Asian population of SGDP groups. The comparison of gene absence frequencies for each gene was performed by Fisher's exact test. Those genes without OR numbers marked are core in SGDP while distributed in gastric cancer group. Note: Tumor, tumor tissues; SGDP, Simons Genome Diversity Project.

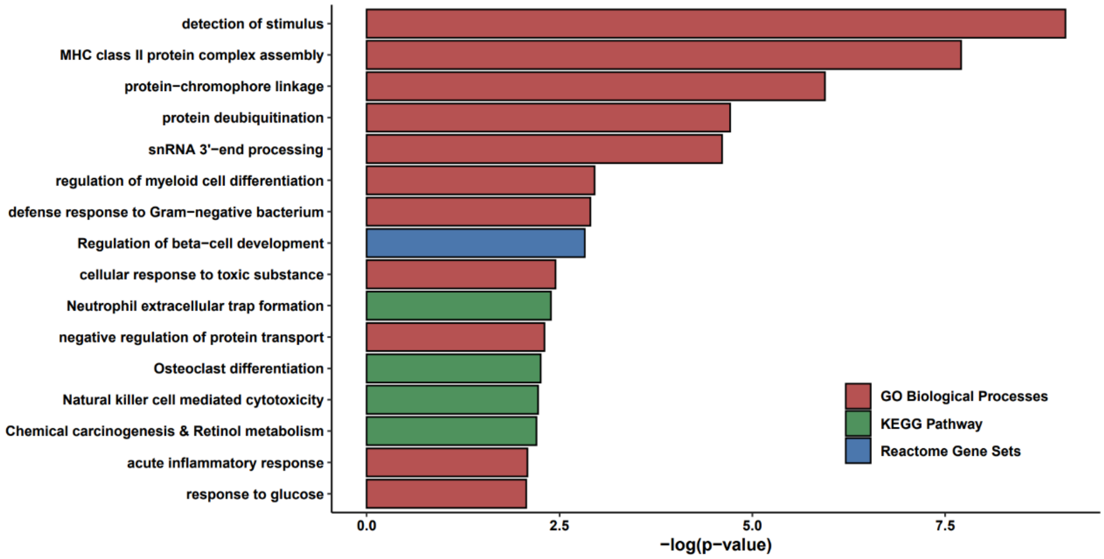

Supplementary Fig. 21 The enriched analysis of 186 distributed genes on GRCh38 using Metascape. Only functions with P values < 0.01 are listed. Note: GO, Gene Ontology; KEGG, Kyoto Encyclopedia of Genes and Genomes.

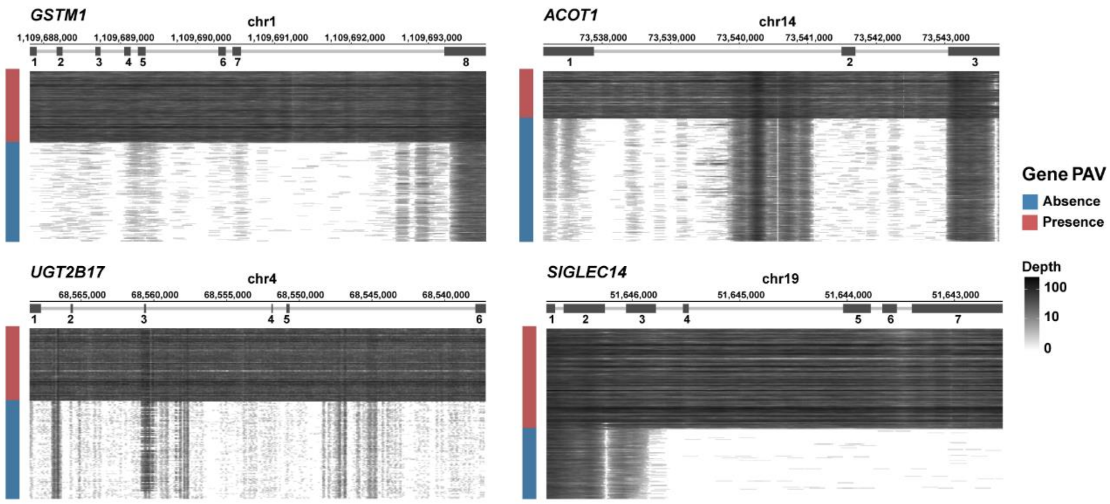

Supplementary Fig. 22 Gene PAVs profile of four highly absent genes of *ACOT1*, *GSTM1*, *UGT2B17*, and *SIGLEC14* in 185 gastric cancer samples. On the top of each heatmap is the gene structure. The black boxes stand for the exons. In the heatmap, the sequencing depth was represented in degree of grey. The bars in the left of heatmaps, blue represents gene absence and red shows gene presence for each sample.

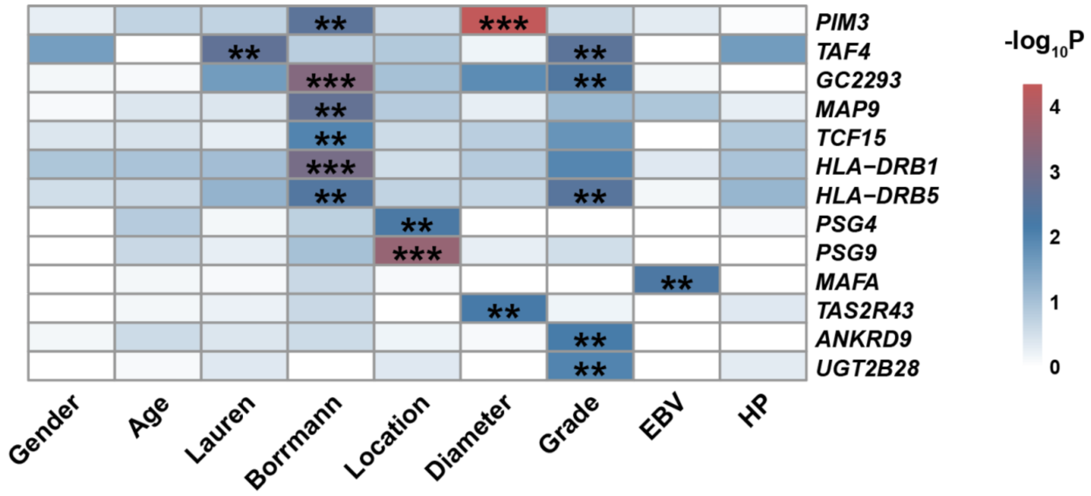

Supplementary Fig. 23 The association of distributed genes and clinical phenotypes on gastric cancer. Fisher exact test was used to calculate the significance of associations between phenotypes and gene PAVs. Note: **, $P \leq 0.01$, ***, $P \leq 0.001$.

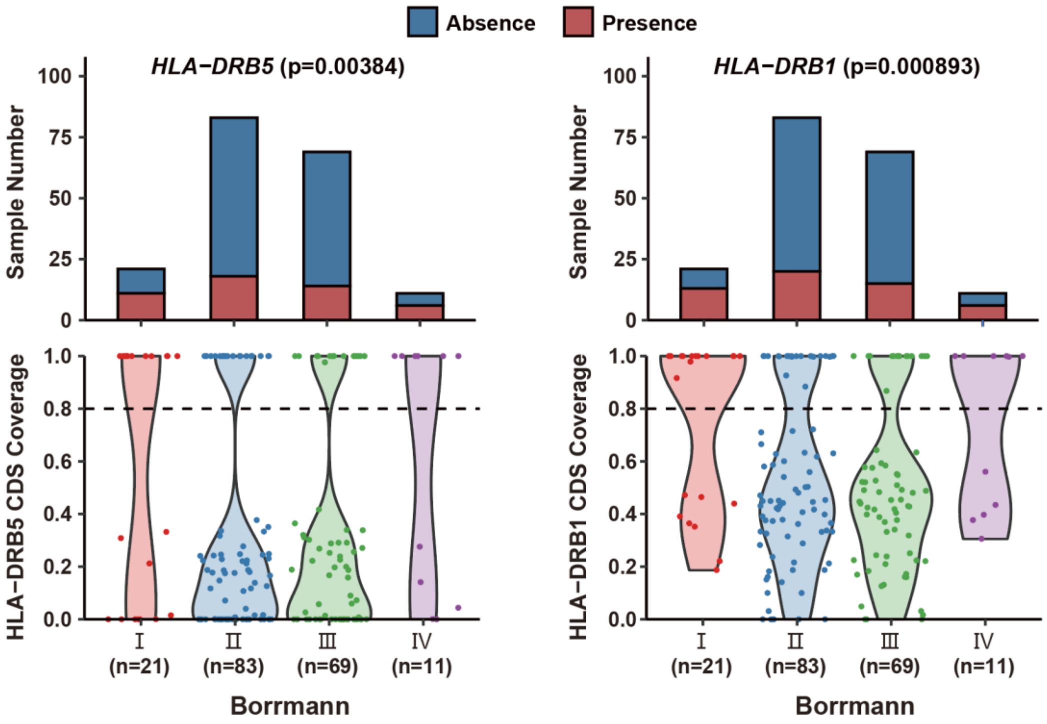

Supplementary Fig. 24 The association of distributed genes (*HLA-DRB5* and *HLA-DRB1*) and Borrmann classification. The ulcerative (Ⅱ) and infiltrative ulcerative types (III) were often occurred in the individuals with gene absence of *HLA-DRB1* and *HLA-DRB5*.

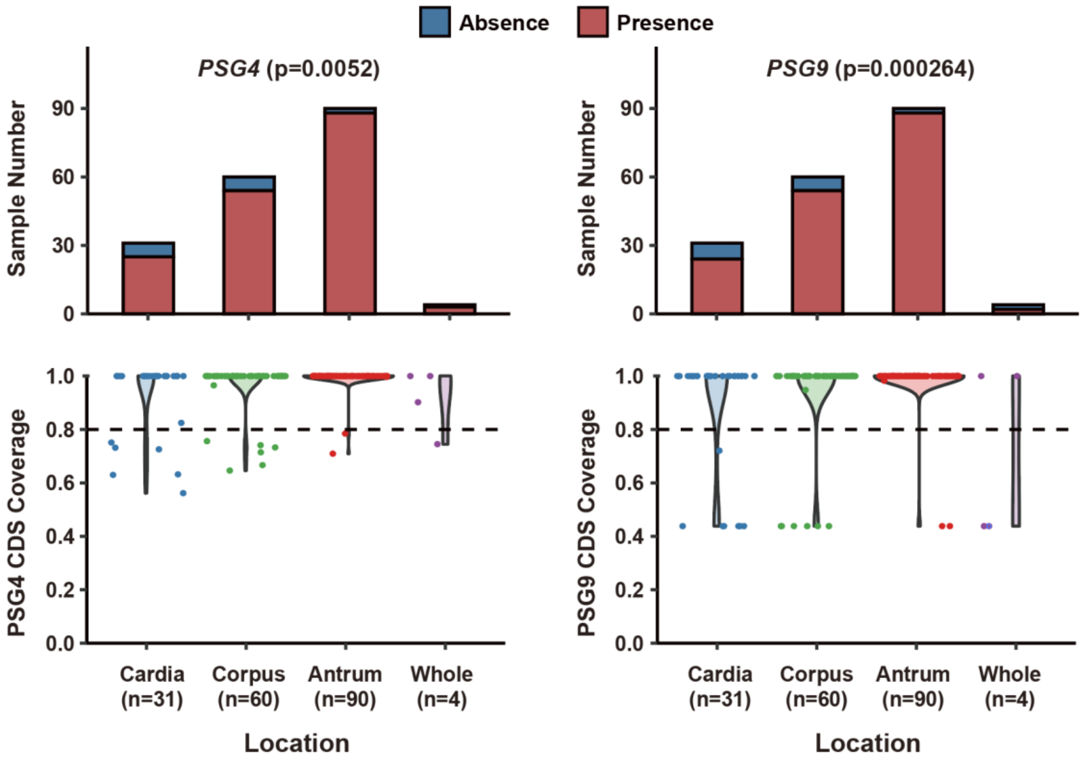

Supplementary Fig. 25 The association of distributed genes (*PSG4* and *PSG9*) and cancer location. The absence of *PSG9* and *PSG4* was closely related to increased incidence of gastric cancer in corpus and cardia of stomach.

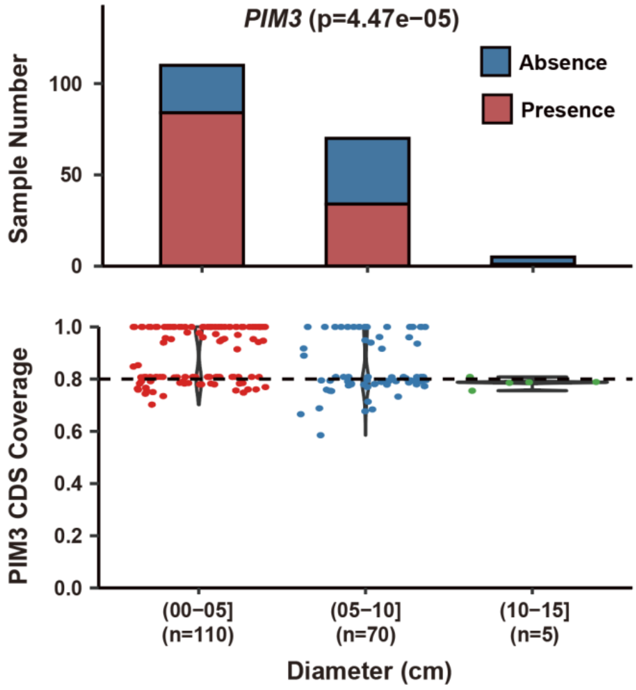

Supplementary Fig. 26 The association of *PIM3* distributed gene and tumor diameter. The higher frequency of *PIM3* absence was related to larger tumor diameter.

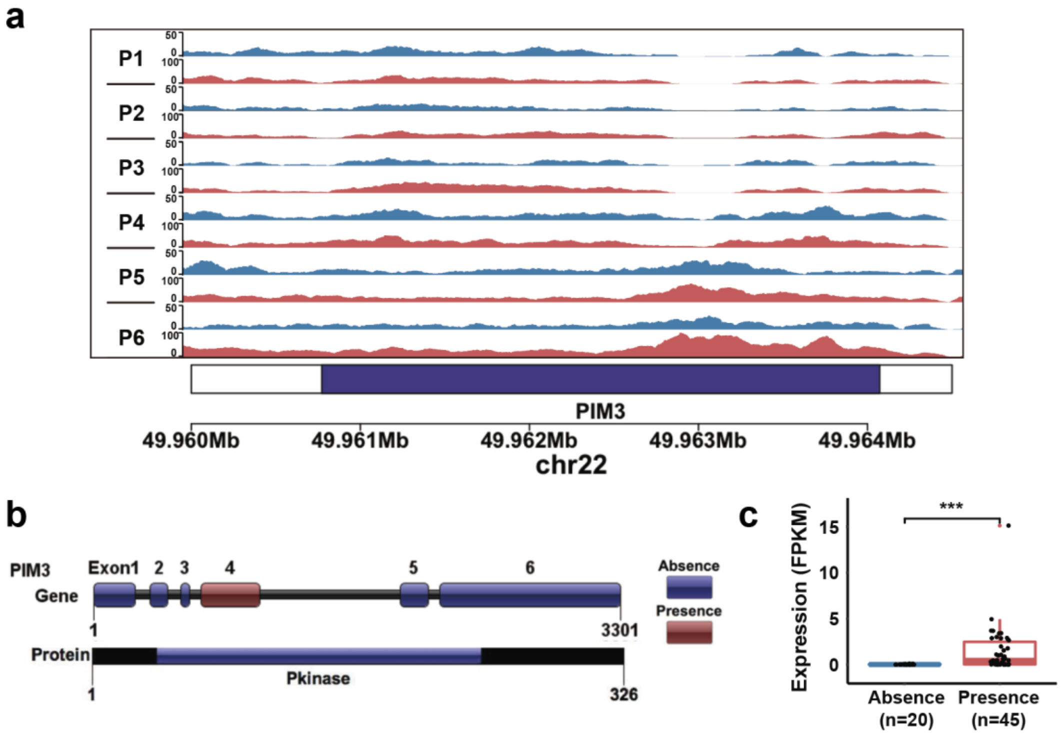

Supplementary Fig. 27 The gene PAVs and expression features of distributed gene *PIM3*. **a** Read coverage of *PIM3* gene in randomly picked six patients. The tracks in blue and red stand for matched normal mucosa and tumor tissue, respectively. **b** The sketches of gene and protein structures. The absence of several exons was found in some individuals. Part of the protein regions corresponding to the protein kinase domain (Pkinase) from UniProt were absent in some individuals. **c** Validation of *PIM3* gene mRNA expression (FPKM) of absence group (n=20) and presence group (n=45) in gastric cancers by RNA-Seq. The center lines of box plots stand for median values, hinges for the first and third quartiles and the whiskers for maxima and minima within 1.5 times of the interquartile range. The comparison was tested by Wilcoxon test (P = 2.4e-5). Note: ***, $P \leq 0.001$.

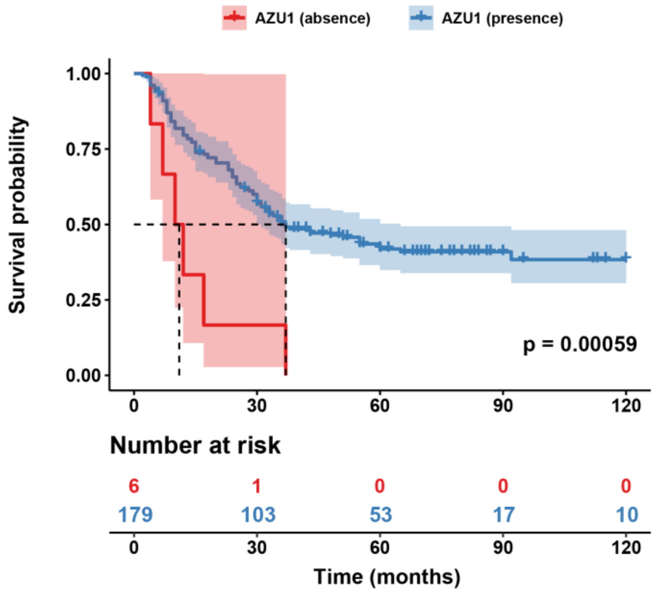

Supplementary Fig. 28 Survival analysis of patients with absence/presence of distributed gene *AZU1*. In Kaplan-Meier analysis, the shaded regions show 95% confidence intervals of the survival curves.

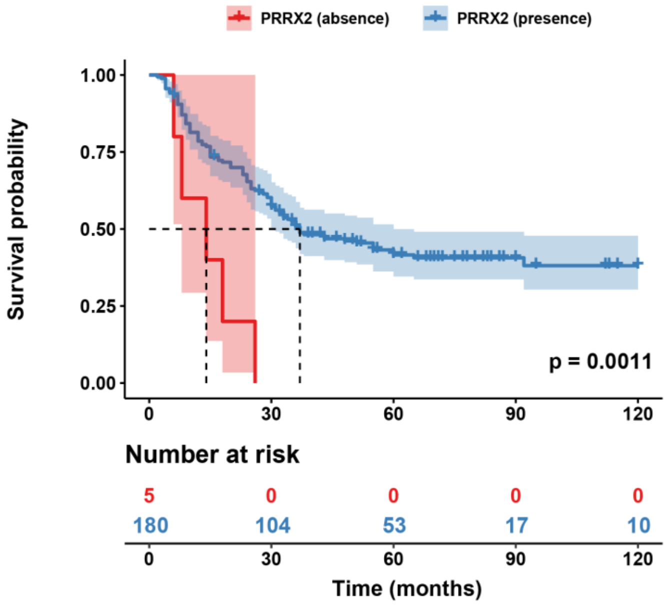

Supplementary Fig. 29 Survival analysis of patients with absence/presence of distributed gene *PRRX2*. In Kaplan-Meier analysis, the shaded regions show 95% confidence intervals of the survival curves.

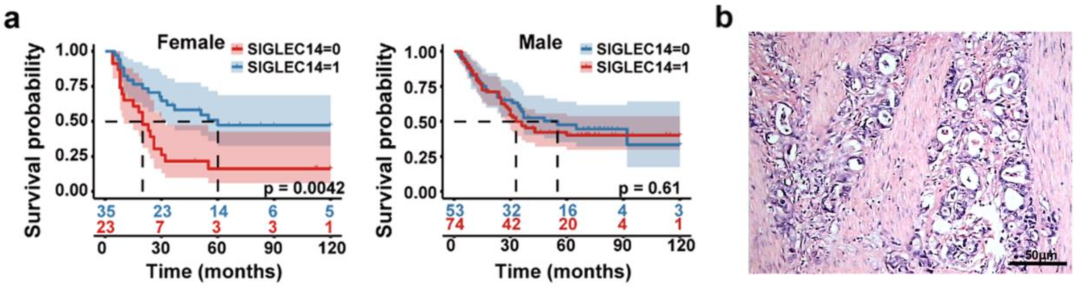

Supplementary Fig. 30 Survival analysis of patients with absence/presence of distributed gene *SIGLEC14*. **a** The absence/presence of *SIGLEC14* is associated with survival of female but not male patients. In Kaplan-Meier analysis, the shaded regions show 95% confidence intervals of the survival curves. **b** A typical histology figure of poorly-differentiated adenocarcinoma cancer in a female patients with *SIGLEC14* absence.

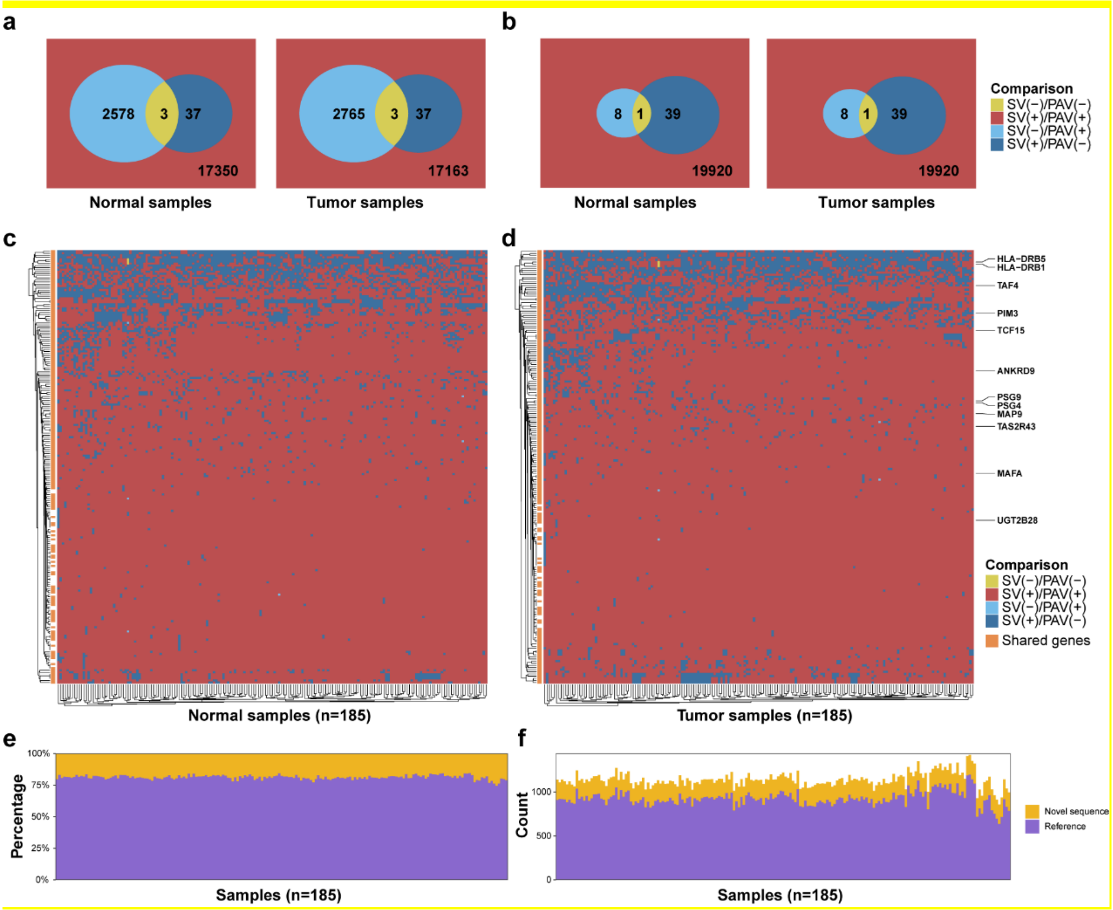

Supplementary Fig. 31 Comparison of GRCh38-based SVs and GCPAN-based PAVs. **a** Venn diagram of genes classified as presence/absence by GRCh38-based SV method and GCPAN-based PAV method, where all deletion SVs were used. **b** The Venn diagram where only homozygous deletion SVs were used in GRCh38-based SV method. Both in subfigures a and b, for each gene category, the gene number was the average value in all samples. **c** The presence/absence patterns of 212 genes identified as absence by GCPAN-based PAV method but presence by GRCh38-based SV method in at least one normal mucosae sample. The twelve distributed genes significantly associated with clinical phenotypes were marked to the right side of the heatmap. Note：(-) represents for gene absence, and (+) represents for gene presence. **d** The 203 genes identified as absence by PAV method but presence by SV method in at least one primary tumor samples. The names of 12 genes associated with tumor phenotypes were listed on the right of the heatmap. **e and f** the ratios (e) and numbers (f) of SV insertion sequences aligned to novel sequences of GCPAN (orange) and SV insertion sequences aligned to the human reference genome GRCh38 (purple).

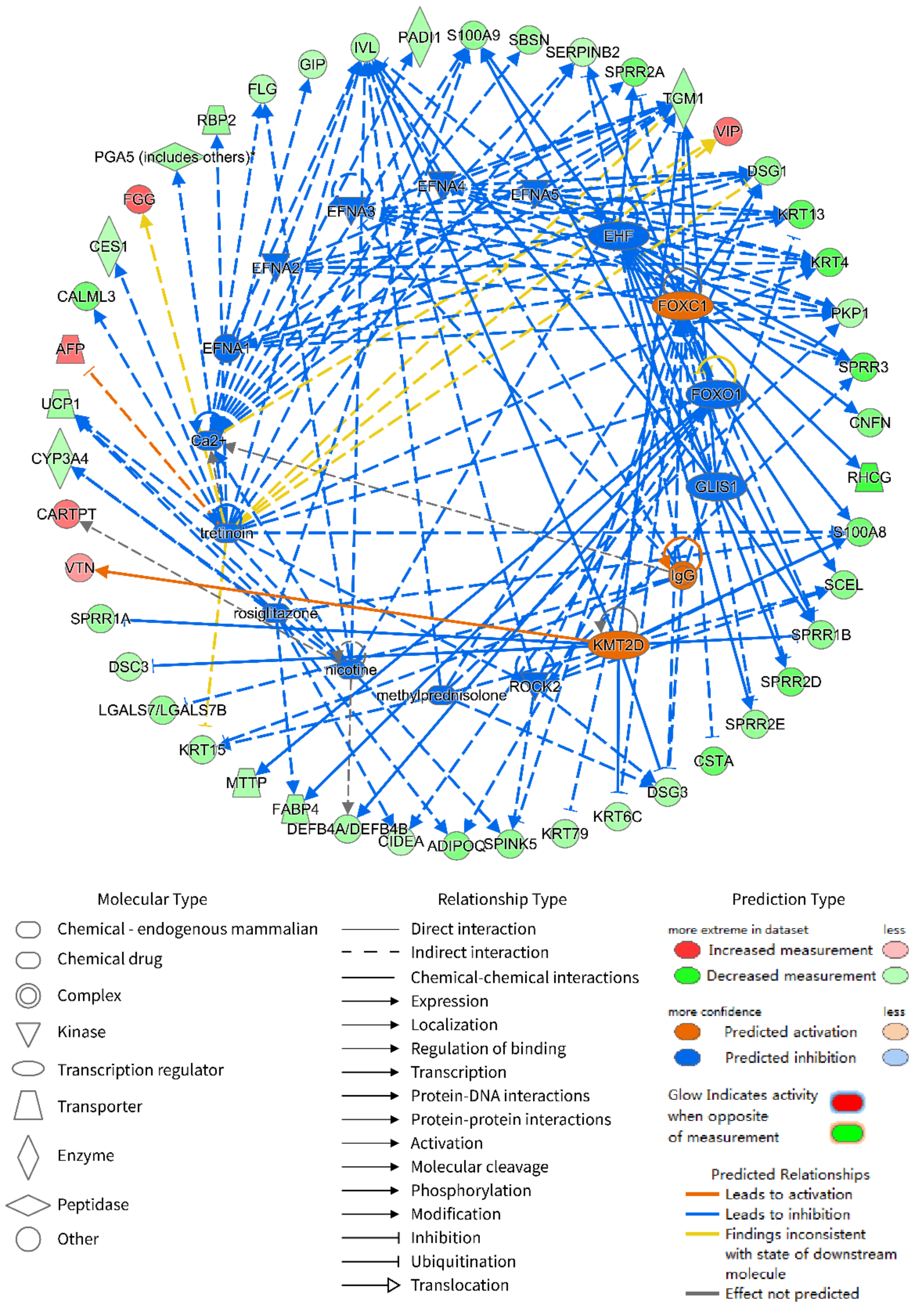

Supplementary Fig. 32 The upstream regulators predicted by IPA for genes related to *GC0643* PAVs. This figure was generated by IPA (version 70750971) upstream regulation analysis. The 10 up-regulated and 138 down-regulated genes with fold change > 3 were input together. The 17 upstream regulators and the relationship with *P*-value less than 0.001 are presented.

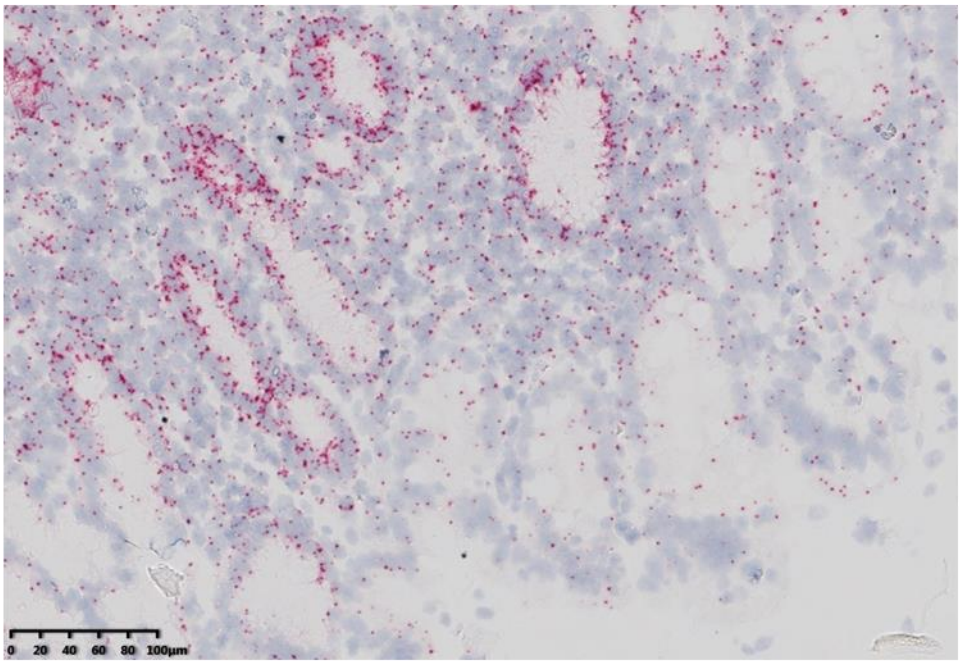

Supplementary Fig. 33 The positive control result targeting housekeeping gene *PPIB* by RNAScope. To examine the mRNA transcript of *GC0643*, the probe targeting housekeeping gene *PPIB* was used to examine the mRNA expression in gastric mucosa tissue. The positive signals present as red dots, indicating that RNA integrity of gastric mucosa is proper for mRNA detection, and the reagent quality is qualified (RNA in situ hybridization, 200×magnification).

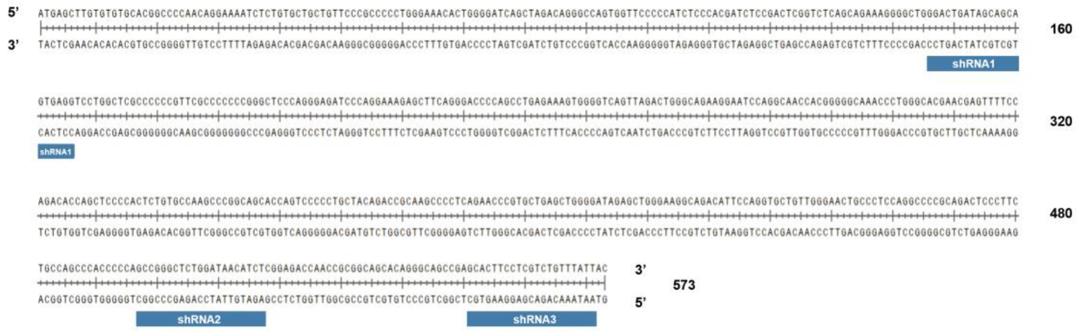

Supplementary Fig. 34 The shRNA interfering sequences targeting *GC0643* gene. The sequences of shRNA2 and shRNA3 showed well efficacy.

The primers of *GC0643* mRNA assay (5’-3’)

Primer name	Sequences (5’to 3’)
H_643-RA-qF	TCTCCCACGATCTCCGACTC
H_643-RA-qR	TGGTTGCCTGGATTCCTTCTG
H_GAPDH-F	GTCTCCTCTGACTTCAACAGCG
H_GAPDH-R	ACCACCCTGTTGCTGTAGCCAA

Supplementary Fig. 35 The primers of *GC0643* mRNA expression detection. Housekeeping gene *GAPDH* was used and internal control.

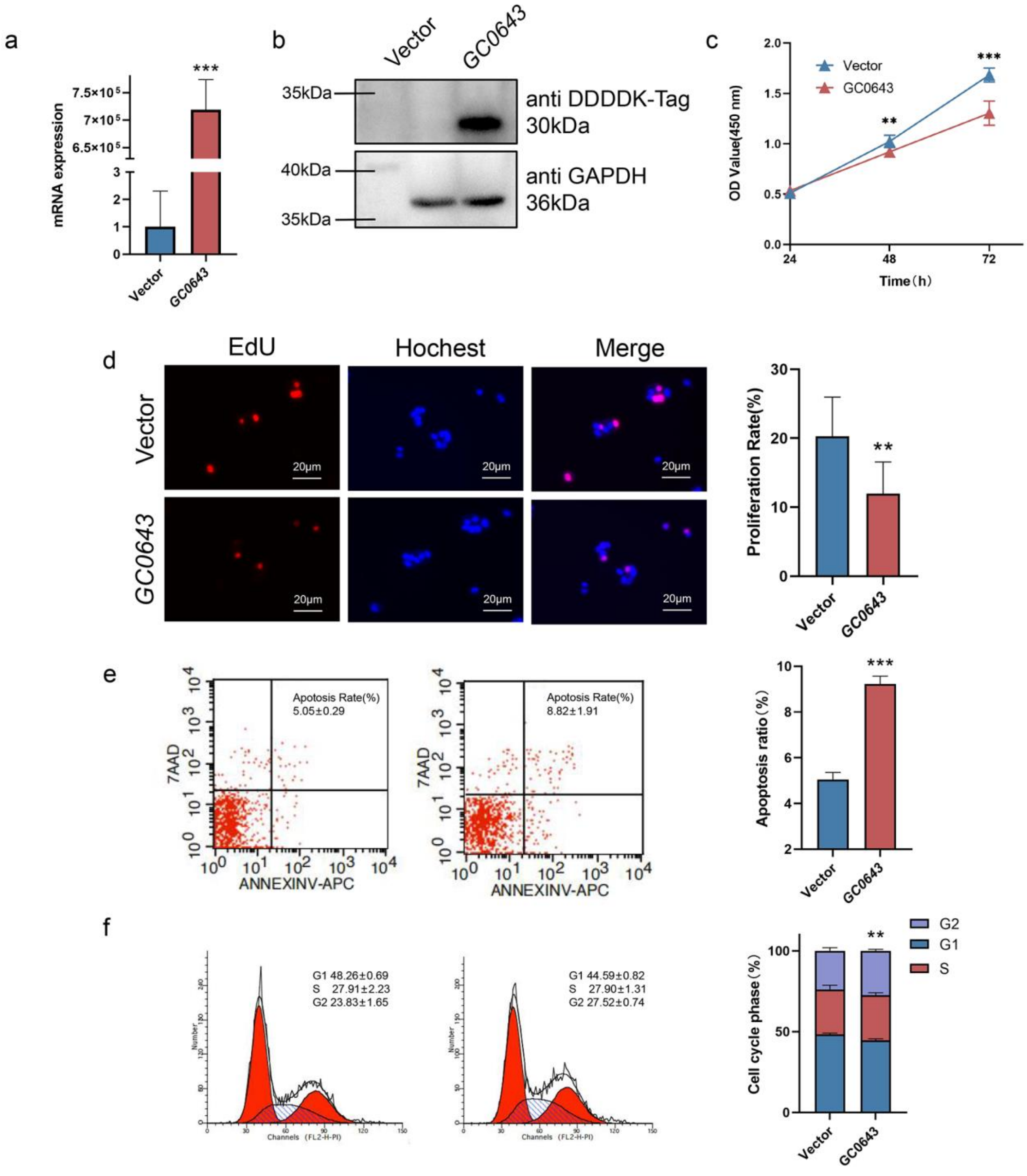

Supplementary Fig. 36 The biological functions of *GC0643* overexpression. **a** Effect of *GC0643* mRNA transcription on NCI-N87 cancer cells by qRT-PCR. Data are presented as mean values ± SD from n = 3 biological replicates. Data were analyzed statistically by two-tailed Student's t test. $^{***}P$ = 2.17E-05. **b** Increased protein level was observed after gene transfection. **c** Cell growth activity was suppressed after gene transfection based on CCK-8 assays. Data are presented as mean values ± SD. of 7 biological replicates. Data were analyzed statistically by two-tailed Student's t test with $^{**}P$ = 0.008, $^{***}P$ = 4.61E-05. **d** Representative images of EdU positive cells and nuclei stained with Hoechest from n = 5 biological replicates. Scale bar represents

20μm. Data are presented as mean values ± SD. of 7 biological replicates. Data were analyzed statistically by two-tailed Student's t test with $^{**}P = 0.007$. **e** Overexpression of *GC0643* induced cell apoptosis. Data are presented as mean values ± SD. of 3 biological replicates. *P* values were derived from two-tailed Student's t test with $^{***}P = 0.0001$. **f** Overexpression of *GC0643* changed cell cycle resulted in G2/M arrest with increased G2/M fraction. Data are presented as mean values ± SD. of 4 biological replicates. *P* values were derived from two-tailed Student's t test with $^{**}P = 0.001$. Source data are provided as a Source Data file.

Supplementary tables

Supplementary Table 1 The parameters used in the process of *de novo* assembly by SGA for this study

SGA step	Parameters
preprocess (preprocess fastq files)	--pe-mode 1
index (index preprocessed data)	-a ropebwt –no-reverse –t 16
correct (error correct preprocessed data)	-k 55 –learn –t 16
index (index error-corrected data)	-a ropebwt –t 16
filter (filter error-corrected data)	-x 2 –t 16
fm-merge (merge reads)	-m 85 –t 16
index (index FM-merged reads)	-d 20000000 –t 16
rmdup (remove duplicates)	-t 16
overlap (construct string graph)	-m 85 –t 16
assemble (contig assemble)	-m 97 –d 0.4 –g 0.1 –r 50 –l 160

Supplementary Table 2 The reads, contigs, and length with HP genome mapping in 52 individuals

Matched normal mucosa				Primary tumor tissue			
Name	#Reads	#Contigs	Length (bp)	Name	#Reads	#Contigs	Length (bp)
GCH1N00031G	188,172	4	12,999	GCH1T00031G	73,177	530	528,395
GCH2N00008G	284,963	9	35,991	GCH2T00008G	94,684	375	657,942
GCH2N00009G	312,923	13	88,601	GCH2T00009G	47,484	331	254,377
GCH2N00011G	436,546	828	843,807	GCH2T00011G	68,339	555	505,473
GCH2N00015G	98,357	100	387,093	GCH2T00015G	653,152	14	110,521
GCH2N00020G	111,160	94	342,049	GCH2T00020G	37,227	137	91,371
GCH2N00022G	193,459	710	792,652	GCH2T00022G	136,053	241	530,175
GCH2N00032G	191,255	16	102,774	GCH2T00032G	18,583	1	622
GCH2N00033G	27,305	9	6,218	GCH2T00033G	34,996	101	66,975
GCH2N00035G	142,110	18	102,334	GCH2T00035G	27,822	44	27,530
GCH2N00039G	20,692	13	8,157	GCH2T00039G	346,244	224	452,513
GCH2N00046G	127,511	28	205,378	GCH2T00046G	25,292	15	8,483
GCH1N00004G	101,332	125	436,842	GCH1T00004G	1,514	0	0
GCH1N00028G	27,159	49	31,933	GCH1T00028G	409	0	0
GCH1N00029G	138,913	36	222,832	GCH1T00029G	80	0	0
GCH1N00039G	16,640	1	549	GCH1T00039G	40	0	0
GCH1N00042G	99,769	184	517,405	GCH1T00042G	517	0	0
GCH1N00050G	160,819	5	31,432	GCH1T00050G	113	0	0
GCH1N00051G	36,678	194	137,444	GCH1T00051G	223	0	0
GCH1N00059G	56,135	482	470,645	GCH1T00059G	65	0	0
GCH1N00060G	64,599	493	426,759	GCH1T00060G	477	0	0
GCH1N00061G	60,064	526	542,250	GCH1T00061G	160	0	0
GCH1N00068G	113,390	46	254,497	GCH1T00068G	66	0	0
GCH1N00078G	110,252	77	329,952	GCH1T00078G	362	0	0
GCH1N00086G	68,171	464	601,895	GCH1T00086G	953	0	0
GCH1N00088G	22,603	24	15,187	GCH1T00088G	934	0	0
GCH1N00090G	26,595	42	25,657	GCH1T00090G	58	0	0
GCH1N00095G	19,473	5	3,024	GCH1T00095G	72	0	0
GCH1N00099G	26,971	63	39,461	GCH1T00099G	42	0	0
GCH1N00100G	43,101	246	180,723	GCH1T00100G	157	0	0
GCH1N00120G	18,641	3	1,822	GCH1T00120G	0	0	0
GCH1N00121G	43,572	175	123,822	GCH1T00121G	158	0	0
GCH1N00133G	116,513	38	216,585	GCH1T00133G	10,698	0	0
GCH1N00138G	46,809	392	308,568	GCH1T00138G	1,488	0	0
GCH2N00001G	21,107	11	6,456	GCH2T00001G	966	0	0
GCH2N00004G	175,453	5	27,162	GCH2T00004G	1,873	0	0
GCH2N00007G	27,883	18	10,790	GCH2T00007G	4,361	0	0
GCH2N00014G	319,285	814	806,442	GCH2T00014G	3,316	0	0
GCH2N00016G	55,040	474	489,000	GCH2T00016G	1,697	0	0
GCH2N00018G	159,440	10	59,647	GCH2T00018G	1,323	0	0

GCH2N00023G	98,289	154	456,140	GCH2T00023G	1,109	0	0
GCH2N00024G	675,399	148	314,375	GCH2T00024G	1,632	0	0
GCH2N00025G	69,544	480	581,697	GCH2T00025G	2,458	0	0
GCH2N00026G	507,372	123	301,467	GCH2T00026G	960	0	0
GCH2N00027G	146,734	9	76,990	GCH2T00027G	559	0	0
GCH2N00029G	126,329	25	116,490	GCH2T00029G	446	0	0
GCH2N00030G	112,832	505	789,770	GCH2T00030G	675	0	0
GCH2N00031G	15,385	1	632	GCH2T00031G	12,459	0	0
GCH2N00034G	15,903	2	1,123	GCH2T00034G	282	0	0
GCH2N00036G	95,881	177	538,122	GCH2T00036G	6,635	0	0
GCH2N00037G	581,853	126	355,832	GCH2T00037G	2,214	0	0
GCH2N00044G	19,942	15	9,223	GCH2T00044G	0	0	0

Supplementary Table 3 The information (reads, contigs, and their lengths) about EBV genome sequences in 8 individuals

Matched normal mucosa				Primary tumor tissue			
Name	#Reads	#Contigs	Length (bp)	Name	#Reads	#Contigs	Length (bp)
GCH1N00006G	8,197	6	49,631	GCH1T00006G	49,645	88	86,232
GCH1N00017G	260	0	0	GCH1T00017G	419,253	111	97,700
GCH1N00102G	63	0	0	GCH1T00102G	396,939	106	96,699
GCH2N00028G	88	0	0	GCH2T00028G	110,367	12	95,906
GCH2N00042G	176	0	0	GCH2T00042G	248,498	127	93,928
GCH2N00014G	118	0	0	GCH2T00014G	514,024	68	57,380
GCH1N00100G	16	0	0	GCH1T00100G	461,023	100	79,891
GCH1N00120G	210	0	0	GCH1T00120G	695,496	24	15,642

Supplementary Table 4 Information about 14 predicted genes

Gene name	Contigs ID	Start	End	Unaligned Contigs	Presence No		Gene Type
					T	N	
GC0574	GCH2T00005G_517089	8942	9760	Partially	107	108	Distributed
GC0575	GCH2T00005G_517089	9874	10461	Partially	167	170	Distributed
GC0643	GCH1N00052G_3295248	8967	16379	Partially	167	168	Distributed
GC1150	GCH1T00132G_5456497	1686	1928	Partially	185	185	Core
GC1732	GCH1T00071G_8237040	1048	1203	Partially	185	185	Core
GC2270	GCH2N00014G_1745986	456	4418	Partially	181	183	Distributed
GC2293	GCH2N00030G_2058889	3485	4165	Fully	146	147	Distributed
GC2644	GCH1N00111G_2258067	1212	2582	Partially	185	185	Core
GC2877	GCH1T00030G_11745596	1488	2775	Partially	175	175	Distributed
GC2964	GCH1T00057G_1950872	1447	1953	Partially	74	75	Distributed
GC2973	GCH1T00139G_13867175	2100	2288	Partially	26	26	Distributed
GC3528	GCH1T00105G_301201	1085	1750	Partially	131	134	Distributed
GC4405	GCH1T00101G_5055647	140	775	Partially	185	185	Core
GC6055	GCH2T00032G_6945605	153	266	Fully	185	185	Core

Supplementary Table 5 The genes with protein coding regions (CDSs) or UTR regions overlapped with partially unaligned sequences*.

	Chromosome	Gene	Region		Unaligned region		
			Start	End	Start	End	
CDS	chr5	*BOD1*	173609239	173609434	173609239		#
	chr6	*HLA-DRB1*	32579091	32579104	32486907	32580133	
	chr6	*HLA-DRB5*	32521905	32522174	32486907	32580133	
	chr6	*HLA-DRB5*	32519370	32519651	32486907	32580133	
	chr6	*HLA-DRB5*	32518556	32518666	32486907	32580133	
	chr6	*HLA-DRB5*	32518054	32518077	32486907	32580133	
	chr6	*HLA-DRB5*	32517739	32517752	32486907	32580133	
	chr6	*HLA-DRB5*	32530125	32530224	32486907	32580133	
	chr11	*MUC6*	1015762	1018770	1017632		#
	chr11	*OR8U1*	56375624	56376553	56375616	56376550	
	chr15	*GOLGA6L2*	23439745	23441682	23440319		#
UTR	chr2	*PRKRA*	178431414	178432096	178431414		#
	chr3	*ROBO1*	79527821	79527887	79527830	79527977	
	chr5	*BOD1*	173609238	173609238	173609239		#
	chr6	*HLA-DRB5*	32517353	32517738	32486907	32580133	
	chr6	*HLA-DRB5*	32530225	32530287	32486907	32580133	
	chr6	*HLA-DRB1*	32578769	32579090	32486907	32580133	
	chr6	*MTO1*	73500736	73509236	73506692		#
	chr6	*MTO1*	73500736	73509236	73506915	73506992	
	chr8	*C8orf34*	68791015	68792571	68792303		#
	chr8	*TMEM68*	55746172	55748115	55746203		#
	chr9	*ABO*	133255176	133255665	133254865	133255322	
	chr9	*MAN1B1*	137101484	137101853	137101832		#
	chr11	*AP2A2*	943100	943314	943119	943230	#
	chr11	*MOGAT2*	75731287	75732953	75731454		#
	chr11	*MOGAT2*	75731287	75732958	75731454		#
	chr15	*GOLGA6L2*	23439498	23441682	23440319		#
	chr16	*MEIOB*	1871993	1872178	1872059		#
	chr16	*MEIOB*	1871993	1872144	1872059		#
	chr16	*MEIOB*	1871993	1872095	1872059		#
	chrX	*KRBOX4*	46497166	46497422	46497278	46497470	

one-end aligned. * The start and end positions of CDS regions and UTR regions overlapped by partially unaligned sequences are listed in the table as well as the start and end positions of unaligned regions. All coordinates are based on the human reference genome (GRCh38).

Supplementary Table 6 Closure of 25 gaps for GRCh38 primary assembly sequences by two-end placed sequences

Gap coordinate	Reference gap length (bp)	Matched normal mucosa				Primary tumor tissue			
		Number	Minimum (bp)	Maximum (bp)	Mean (bp)	Number	Minimum (bp)	Maximum (bp)	Mean (bp)
chr1:223558936-223608935	50,000	61	812	836	828	94	814	836	830
chr4:8797478-8816477	19,000	23	732	750	749	7	741	750	749
chr6:167591394-167641393	50,000	1	587	587	587	NA	NA	NA	NA
chr9:43332175-43333269	1,095	33	1,249	1,311	1,268	38	1,249	1,311	1,262
chr10:38906037-38911580	5,544	2	5,737	5,767	5,752	NA	NA	NA	NA
chr12:37379852-37380460	609	101	641	641	641	79	641	641	641
chr13:113673021-113723020	50,000	1	712	712	712	NA	NA	NA	NA
chr17:490396-491111	716	NA	NA	NA	NA	2	738	742	740
chr17:21984550-21985100	551	2	748	758	753	6	743	758	750
chr18:54536575-54537528	954	4	918	918	918	1	918	918	918
chr20:63216-63840	625	39	599	604	601	42	599	604	600
chr20:36314372-36314719	348	53	1,971	1,973	1,972	47	1,971	1,973	1,972
chr22:49973866-49975365	1,500	6	1,491	1,848	1,617	13	1,491	1,848	1,660
chrX:50228965-50278964	50,000	NA	NA	NA	NA	2	3,619	3,646	3,633
chrY:9046915-9055174	8,260	65	3,186	3,186	3,186	74	3,186	3,186	3,186
chrY:10633441-10645833	12,393	2	1,412	1,427	1,420	12	1,326	2,222	1,669
chrY:10649990-10651421	1,432	2	1,412	1,427	1,420	12	1,326	2,222	1,669
chrY:10896526-10898184	1,659	5	1,228	1,228	1,228	37	1,228	1,228	1,228
chrY:10908520-10909101	582	14	4,177	4,504	4,481	10	4,177	4,504	4,341
chrY:10922487-10923564	1,078	9	698	763	705	32	698	763	700

chrY:11016993-11017247	255	56	595	595	595	72	595	595	595
chrY:11023375-11024266	892	7	857	891	876	9	857	857	857
chrY:11028343-11029160	818	8	748	753	752	17	748	753	753
chrY:11647443-11647709	267	56	692	727	717	65	692	727	717
chrY:11663987-11664006	20	9	1,460	1,460	1,460	8	1,460	1,460	1,460

NA, Not available.

Supplementary Table 7 Extension of 85 ends of 69 gaps for GRCh38 primary assembly sequences by one-end placed sequences

Gap coordinate	Gap size (bp)	Status	Length (bp)
chr2:16145120-16146119	1000	left	1682
chr4:49658101-49708100	50000	left	964
chr4:49711962-49712061	100	left	804
chr4:51418957-51419056	100	left	1484
chr5:47079734-47082080	2347	right	2293
chr5:49600987-49601086	100	right	609
chr5:49600987-49601086	100	left	1213
chr6:59829935-60229934	400000	right	2133
chr6:61370555-61371372	818	right	575
chr6:61398162-61398261	100	right	534
chr6:95020791-95070790	50000	left	1408
chr6:95020791-95070790	50000	right	1106
chr7:62456780-62506779	50000	left	8463
chr10:38529908-38573338	43431	left	552
chr10:38529908-38573338	43431	right	4208
chr10:38913439-38918269	4831	left	1219
chr10:39409793-39410237	445	right	553
chr10:39409793-39410237	445	left	522
chr11:50821349-51078348	257000	left	9102
chr11:70955697-71055696	100000	right	12059
chr11:70955697-71055696	100000	left	3895
chr12:34719408-34769407	50000	left	1698
chr13:16282074-16282173	100	left	628
chr13:111793442-111843441	50000	right	704
chr13:111793442-111843441	50000	left	9550
chr14:16096531-16096630	100	right	587
chr14:16096531-16096630	100	left	8407
chr14:16105377-16113232	7856	left	1297
chr15:17083574-17083673	100	left	5863
chr17:21795851-21814103	18253	right	6266
chr17:21992062-22042061	50000	left	3385
chr17:26643469-26643843	375	right	503
chr17:26805756-26805775	20	left	18948
chr17:26880255-26880354	100	left	3669
chr17:26885981-26935980	50000	right	909
chr19:24898314-24904771	6458	left	1051
chr19:24898314-24904771	6458	right	738
chr20:26364241-26365414	1174	left	1221
chr20:26608046-26608145	100	right	595
chr20:28508898-28508997	100	right	1963
chr20:28728875-28728974	100	right	5834

chr20:28751120-28752590	1471	left	759
chr20:28867525-28868452	928	left	584
chr20:28890336-28896362	6027	left	1189
chr20:29447839-29447883	45	left	789
chr20:29452159-29452178	20	right	2568
chr20:29540235-29540284	50	left	815
chr20:30425129-30456077	30949	left	599
chr20:30425129-30456077	30949	right	702
chr20:31001509-31051508	50000	right	616
chr20:31107037-31157036	50000	right	913
chr20:31159120-31161625	2506	right	4359
chr20:31159120-31161625	2506	left	1047
chr21:7865747-7915746	50000	right	1986
chr21:43212463-43262462	50000	left	616
chr22:11160922-11210921	50000	right	1001
chr22:11378057-11428056	50000	left	1753
chr22:11631289-11681288	50000	left	2221
chr22:16313517-16314010	494	left	509
chr22:16313517-16314010	494	right	531
chrX:114281199-114331198	50000	left	1488
chrX:116557780-116595566	37787	left	599
chrX:144425607-144475606	50000	right	2609
chrY:9057609-9107608	50000	left	2903
chrY:9057609-9107608	50000	right	4388
chrY:9111869-9112715	847	left	578
chrY:9114320-9116371	2052	left	979
chrY:10669738-10670732	995	right	554
chrY:10674059-10676544	2486	right	735
chrY:10674059-10676544	2486	left	574
chrY:10679716-10682442	2727	left	739
chrY:10679716-10682442	2727	right	1207
chrY:10694193-10744192	50000	right	1197
chrY:10694193-10744192	50000	left	1005
chrY:10816759-10817292	534	right	519
chrY:10961626-10962533	908	right	570
chrY:10965695-10967284	1590	left	2129
chrY:10965695-10967284	1590	right	1157
chrY:11012529-11013046	518	left	6867
chrY:11592903-11642902	50000	right	4496
chrY:11660375-11662181	1807	left	52171
chrY:11669949-11670088	140	right	13086
chrY:11671801-11671820	20	right	29068
chrY:11673796-11674123	328	right	31837
chrY:21789282-21805281	16000	right	14766

Supplementary Table 8 Hit of 10 predicted genes with proteomic sequences from 80 diffuse gastric cancer samples in CPTAC dataset

Gene name	Number of supporting patients (gene)	Peptide sequences	Number of supporting patients (peptide)	Patient IDs
GC0574	16	WTPGQPVRSQMGPPPTGAAR	16	#3; #5; #6; #7; #8; #11; #23; #24; #28; #31; #37; #48; #67; #68; #79; #80
GC0575	12	PPPPTQGK	6	#13; #26; #37; #53; #56; #61
		QGNITQGGRALPDTNAQDR	3	#38; #49; #55
		SRDELAAMRAHSGCR	2	#13; #53
		TARPVPRSRDELAAMR	3	#47; #51; #64
GC0643	2	SLCVHGPNRKISVLLFPPPGK	2	#65; #70
GC1150	5	ATFNLSVFIGQK	2	#28; #31
		MFLERVWSLVR	3	#27; #75; #76
GC2270	24	AIHEVRNQYPGIANR	3	#5; #7; #8
		FPGITAR	4	#15; #20; #41; #48
		HEVRNQYPGIANR	2	#52; #54
		KRTTVTR	2	#18; #45
		LPQQAGR	5	#11; #52; #54; #60; #62
		NQYPGIANR	5	#3; #7; #8; #10; #47
		REGLEHSR	5	#22; #29; #42; #65; #70
		VAIHEVRNQYPGIANR	8	#5; #7; #8; #10; #22; #52; #54; #60
		VQNWFQNER	2	#27; #58
GC2293	9	ALPGLVPQPRR	2	#25; #69
		ALPGLVPQPRRLHPNPLMR	3	#1; #31; #66
		KAGKNGPPSLDPRPPSSSEALR	2	#67; #68
		RLHPNPLMR	2	#6; #16
GC2644	2	MVPSLSLPVSKPATLQTPQVAK	2	#38; #55
GC2964	7	AGEGPRAR	3	#39; #65; #70

		MAGEGPRAR	4	#11; #13; #16; #19
GC3528	9	AGAIMPPAGVGKKPRGQK	2	#56; #61
		GAIMPPAGVGK	3	#26; #45; #47
		GSEGKK	2	#21; #24
		TQKATVAAAK	2	#77; #78
GC4405	2	RGSGGK	2	#65; #70

Supplementary Table 9 The absence status of 186 distributed genes of GRCh38 shared in tumor tissue and matched normal mucosa

Chr	Gene name	Absence Number		Chr	Gene name	Absence Number	
		Normal	Tumor			Normal	Tumor
chr1	*B3GALT6*	28	23	chr11	*OR4C11*	26	27
chr1	*VWA1*	1	3	chr11	*OR4P4*	26	27
chr1	*FNDC10*	21	19	chr11	*OR4S2*	26	27
chr1	*PRAMEF11*	1	1	chr11	*OR8U1*	19	23
chr1	*PRAMEF13*	7	7	chr11	*TRIM64*	95	119
chr1	*PRAMEF14*	16	16	chr11	*UBTFL1*	12	16
chr1	*RHD*	1	1	chr12	*KLRC2*	11	13
chr1	*POU3F1*	1	2	chr12	*TAS2R43*	7	8
chr1	*GBP3*	9	9	chr13	*POU4F1*	22	26
chr1	*DNTTIP2*	15	3	chr13	*SOX1*	44	51
chr1	*GSTM1*	107	107	chr14	*DHRS4L2*	1	1
chr1	*AC253572.1*	10	10	chr14	*ACOT1*	132	133
chr1	*HIST2H3PS2*	2	2	chr14	*ANKRD9*	13	9
chr1	*PPIAL4H*	6	6	chr15	*AC134980.3*	4	4
chr1	*LCE3C*	69	69	chr15	*OR4M2*	6	7
chr1	*LCE3B*	69	69	chr15	*OR4N4*	4	4
chr1	*FCGR3B*	4	1	chr15	*TJP1*	1	1
chr1	*OR2T10*	13	13	chr16	*TP53TG3F*	1	1
chr1	*OR2T11*	13	13	chr16	*HP*	1	1
chr1	*OR2T27*	9	12	chr16	*HPR*	1	1
chr2	*CYS1*	7	4	chr16	*ZFPM1*	9	17
chr2	*PKDCC*	2	1	chr17	*NATD1*	13	18
chr2	*FOXI3*	1	1	chr17	*COPRS*	5	4
chr2	*POU3F3*	64	72	chr17	*TBC1D3F*	1	1
chr2	*C2orf72*	62	51	chr17	*KRTAP9-6*	30	28
chr2	*AQP12A*	3	3	chr17	*IFI35*	24	13
chr3	*CDV3*	6	9	chr17	*LRRC37A*	1	4
chr4	*ZNF718*	115	115	chr17	*ARL17A*	6	2
chr4	*TMEM271*	4	4	chr17	*C17orf58*	9	8
chr4	*NAT8L*	12	17	chr17	*MXRA7*	22	20

chr4	*FAM90A26*	145	146	chr18	*KCNG2*	3	5
chr4	*USP17L10*	160	160	chr19	*HCN2*	11	18
chr4	*USP17L23*	1	1	chr19	*PLPPR3*	4	3
chr4	*UGT2B17*	106	106	chr19	*AZU1*	5	6
chr4	*UGT2B28*	1	2	chr19	*EFNA2*	28	22
chr4	*MAP9*	4	14	chr19	*MEX3D*	37	27
chr5	*FOXD1*	23	19	chr19	*ONECUT3*	63	62
chr5	*TCF7*	7	11	chr19	*KLF16*	68	67
chr5	*PCDHA8*	1	1	chr19	*SAMD1*	1	1
chr5	*NEURL1B*	2	1	chr19	*GDF1*	7	12
chr5	*BTNL3*	17	18	chr19	*CERS1*	7	12
chr6	*FOXQ1*	13	12	chr19	*WTIP*	3	1
chr6	*HIST1H4B*	51	55	chr19	*GPR42*	32	39
chr6	*HIST1H2AB*	66	65	chr19	*PSG4*	13	15
chr6	*HIST1H3C*	25	27	chr19	*PSG9*	16	17
chr6	*MUC22*	2	2	chr19	*IGFL3*	2	2
chr6	*HLA-DRB5*	135	135	chr19	*DACT3*	3	5
chr6	*HLA-DRB1*	129	130	chr19	*FGF21*	101	121
chr6	*HLA-DQA1*	21	17	chr19	*SIGLEC14*	76	76
chr6	*HLA-DQB1*	113	113	chr19	*ZNF83*	11	11
chr6	*HLA-DPB1*	56	55	chr19	*LILRB3*	6	4
chr6	*MARCKS*	1	1	chr19	*LILRA6*	6	4
chr7	*UNCX*	3	6	chr19	*KIR2DL3*	1	1
chr7	*HOXA13*	1	1	chr19	*TMEM238*	13	5
chr7	*ZNRF2*	7	3	chr19	*GALP*	13	12
chr7	*PPP1R17*	11	5	chr20	*TCF15*	29	31
chr7	*ZNF138*	10	6	chr20	*AL049634.2*	37	32
chr8	*OR4F21*	4	1	chr20	*INSM1*	3	1
chr8	*USP17L1*	1	2	chr20	*TAF4*	93	92
chr8	*AC134684.11*	83	84	chr20	*TCFL5*	8	10
chr8	*AC134684.8*	127	143	chr21	*KCNE1B*	8	8
chr8	*AC134684.9*	1	1	chr21	*GATD3A*	5	4
chr8	*AC084121.11*	65	59	chr21	*PFKL*	8	5
chr8	*AC084121.6*	30	30	chr22	*POTEH*	2	2
chr8	*AC084121.12*	27	28	chr22	*AC023490.4*	76	84

chr8	*AC084121.8*	29	27	chr22	*GSC2*	16	20
chr8	*AC084121.7*	67	60	chr22	*NCF4*	13	13
chr8	*AC084121.5*	11	9	chr22	*GALR3*	1	1
chr8	*AC084121.13*	73	75	chr22	*APOBEC3B*	23	23
chr8	*ZNF705B*	54	56	chr22	*FAM19A5*	30	33
chr8	*USP17L8*	102	107	chr22	*PIM3*	60	66
chr8	*USP17L3*	7	5	chrX	*GAGE12E*	13	13
chr8	*ZNF705D*	14	19	chrX	*GAGE12H*	3	1
chr8	*USP17L7*	22	23	chrX	*DGAT2L6*	1	1
chr8	*USP17L2*	18	17	chrX	*RHOXF2B*	2	2
chr8	*PTK2B*	4	3	chrX	*AC008162.2*	59	77
chr8	*MAFA*	6	3	chrX	*CT47A6*	2	3
chr8	*SCX*	15	12	chrX	*ACTRT1*	1	1
chr9	*WASHC1*	3	2	chrX	*CT45A2*	85	88
chr9	*SPATA31A6*	7	5	chrX	*CT45A8*	86	80
chr9	*AL391987.2*	30	24	chrX	*CT45A9*	81	82
chr9	*PRRX2*	7	5	chrX	*CT45A10*	3	2
chr9	*ABO*	6	6	chrX	*SPANXC*	39	47
chr10	*C10orf143*	11	19	chrX	*MAGEA3*	1	1
chr11	*SSU72P5*	2	1	chrX	*OPN1LW*	1	1
chr11	*SSU72P2*	1	1	chrX	*OPN1MW*	1	2
chr11	*OR51A2*	18	18	chrX	*OPN1MW2*	2	1
chr11	*HBG1*	3	4	chrX	*OPN1MW3*	2	1
chr11	*OR52N5*	8	9	chrY	*BPY2*	1	1
chr11	*OR52N1*	1	3	chrY	*CDY1B*	1	1
chr11	*OR5P2*	3	3	chrY	*BPY2B*	1	1
chr11	*MRGPRX1*	1	1	chrY	*BPY2C*	1	1
chr11	*TRIM48*	15	15	chrY	*CDY1*	1	1

Supplementary Table 10 The absence status of 9 distributed predicted genes located at non-reference sequence shared in tumor tissue and matched normal mucosae

Gene name	Absence number		Gene name	Absence number	
	Normal	Tumor		Normal	Tumor
GC2973	159	159	*GC0643*	17	18
GC2964	110	111	*GC0575*	15	18
GC0574	77	78	*GC2877*	10	10
GC3528	51	54	*GC2270*	2	4
GC2293	38	39			

Supplementary Table 11 The absence status of 36 distributed genes of GRCh38 from normal mucosa

Chr	Gene name	Absence number	Chr	Gene name	Absence number
chr1	*RNF187*	3	chr12	*PTPN6*	1
chr1	*CITED4*	1	chr12	*MAGOHB*	1
chr1	*OR4F29*	1	chr13	*METTL21C*	2
chr1	*CAMK2N1*	2	chr15	*KLF13*	3
chr1	*FOXE3*	1	chr16	*TP53TG3E*	1
chr2	*UCN*	1	chr17	*UBALD2*	2
chr2	*FOXD4L1*	1	chr19	*CEBPA*	1
chr3	*TMEM158*	1	chr19	*CTU1*	2
chr4	*HMX1*	1	chr20	*SYNDIG1*	1
chr5	*AC026740.3*	1	chr21	*CRYAA2*	2
chr5	*ZDHHC11B*	1	chr22	*PDXP*	1
chr5	*UBE2QL1*	3	chrX	*VCX3B*	2
chr6	*HIST1H3B*	1	chrX	*NYX*	2
chr7	*POLR2J2*	1	chrX	*AC235565.2*	1
chr7	*AC105052.3*	1	chrX	*CSAG2*	1
chr7	*ZNF316*	3	chrX	*GAGE12C*	1
chr7	*LRRC17*	1	chrX	*F8A3*	1
chr9	*TPRN*	1			
chr12	*HRK*	2			

Supplementary Table 12 The absence status of 30 distributed genes of GRCh38 from tumor tissues

Chr	Gene name	Absence number	Chr	Gene name	Absence number
chr1	*PRAMEF1*	1	chr17	*FAM171A2*	1
chr1	*PRAMEF4*	1	chr18	*RAB12*	1
chr2	*SMIM39*	1	chr19	*KIR3DL1*	1
chr2	*HOXD11*	1	chr19	*AC008554.1*	1
chr2	*IL1B*	1	chr19	*GRIN2D*	2
chr4	*USP17L13*	1	chr19	*CD177*	1
chr5	*TAF11L2*	1	chr20	*NKX2-4*	1
chr9	*RLN1*	1	chr21	*PWP2*	1
chr10	*SPRN*	1	chr21	*U2AF1L5*	2
chr11	*BET1L*	1	chr22	*AP000552.3*	1
chr12	*BRI3BP*	1	chr22	*TBX1*	1
chr14	*FOXG1*	1	chr22	*APOBEC3F*	1
chr16	*FOXC2*	1	chrY	*EIF1AY*	1
chr16	*HS3ST4*	1	chrY	*SRY*	1
chr16	*TP53TG3*	1			
chr17	*BHLHA9*	2			

Supplementary Table 13 The significantly different PAVs of 78 distributed genes on GRCh38 between our group and SGDP group#, *

Gene		Number of gene absence			Adjusted p-values	
Chromosome	Name	Normal# (n=185)	Tumor# (n=185)	SGDP (n=263)	Normal/SGDP	Tumor/SGDP
chr14	*ACOT1*	132	133	88	2.08E-14	8.61E-15
chr8	*AC134684.8*	127	143	125	3.17E-05	1.10E-09
chr1	*GSTM1*	107	107	119	2.18E-02	2.39E-02
chr4	*UGT2B17*	106	106	98	1.12E-04	1.02E-04
chr19	*FGF21*	101	121	45	1.87E-15	3.62E-24
chr11	*TRIM64*	95	119	0	3.24E-43	1.41E-58
chr20	*TAF4*	93	92	0	2.43E-42	9.32E-42
chrX	*CT45A8*	86	80	24	2.15E-18	6.12E-16
chrX	*CT45A2*	85	88	20	8.02E-20	2.48E-21
chr8	*AC134684.11*	83	84	81	6.92E-03	5.42E-03
chrX	*CT45A9*	81	82	16	1.39E-20	4.11E-21
chr22	*AC023490.4*	76	84	11	1.43E-21	2.36E-25
chr19	*SIGLEC14*	76	76	37	1.00E-09	1.08E-09
chr8	*AC084121.13*	73	75	36	4.73E-09	9.79E-10
chr19	*KLF16*	68	67	0	7.08E-29	1.72E-28
chr8	*AC084121.7*	67	60	23	1.68E-11	2.97E-09
chr6	*HIST1H2AB*	66	65	8	1.71E-19	3.99E-19
chr8	*AC084121.11*	65	59	26	7.16E-10	4.67E-08
chr2	*POU3F3*	64	72	0	5.58E-27	6.14E-31
chr19	*ONECUT3*	63	62	0	1.41E-26	4.42E-26
chr2	*C2orf72*	62	51	0	3.68E-26	4.11E-21
chr22	*PIM3*	60	66	10	1.97E-15	4.13E-18
chrX	*AC008162.2*	59	77	36	2.70E-05	2.76E-10
chr6	*HLA-DPB1*	56	55	31	7.21E-06	1.10E-05
chr8	*ZNF705B*	54	56	17	1.00E-09	2.76E-10
chr6	*HIST1H4B*	51	55	7	6.89E-14	1.17E-15
chr13	*SOX1*	44	51	0	6.59E-18	4.11E-21
chrX	*SPANXC*	39	47	12	4.52E-07	1.10E-09
chr19	*MEX3D*	37	27	0	7.37E-15	1.24E-10
chr19	*GPR42*	32	39	4	5.55E-09	1.76E-11
chr8	*AC084121.6*	30	30	1	1.13E-10	1.24E-10
chr9	*AL391987.2*	30	24	2	1.00E-09	1.42E-07
chr22	*FAM19A5*	30	33	11	6.93E-05	1.10E-05
chr17	*KRTAP9-6*	30	28	103	5.67E-07	1.35E-07
chr8	*AC084121.8*	29	27	1	2.81E-10	1.53E-09
chr20	*TCF15*	29	31	0	1.77E-11	2.73E-12
chr1	*B3GALT6*	28	23	0	4.28E-11	3.87E-09
chr19	*EFNA2*	28	22	0	4.28E-11	9.79E-09
chr8	*AC084121.12*	27	28	3	7.00E-08	3.19E-08

chr6	*HIST1H3C*	25	27	3	3.83E-07	6.81E-08
chr17	*IFI35*	24	13	0	1.58E-09	2.71E-05
chr5	*FOXD1*	23	19	0	4.00E-09	1.35E-07
chr17	*MXRA7*	22	20	0	9.20E-09	5.69E-08
chr13	*POU4F1*	22	26	0	9.20E-09	2.76E-10
chr1	*FNDC10*	21	19	0	2.26E-08	1.35E-07
chr6	*HLA-DQA1*	21	17	59	6.49E-03	5.72E-04
chr11	*OR8U1*	19	23	6	1.54E-03	6.80E-05
chr22	*GSC2*	16	20	0	2.24E-06	5.69E-08
chr1	*PRAMEF14*	16	16	5	3.25E-03	3.20E-03
chr8	*SCX*	15	12	0	5.42E-06	6.35E-05
chr8	*ZNF705D*	14	19	1	1.02E-04	1.43E-06
chr14	*ANKRD9*	13	9	0	2.92E-05	8.89E-04
chr6	*FOXQ1*	13	12	0	2.92E-05	6.35E-05
chrX	*GAGE12E*	13	13	0	2.92E-05	2.71E-05
chr19	*GALP*	13	12	3	3.56E-03	6.87E-03
chr17	*NATD1*	13	18	0	2.92E-05	3.17E-07
chr22	*NCF4*	13	13	0	2.92E-05	2.71E-05
chr19	*TMEM238*	13	5	0	2.92E-05	2.67E-02
chr4	*NAT8L*	12	17	0	6.95E-05	7.78E-07
chr11	*UBTFL1*	12	16	1	5.24E-04	1.88E-05
chr8	*AC084121.5*	11	9	1	1.14E-03	5.42E-03
chr10	*C10orf143*	11	19	1	1.14E-03	1.43E-06
chr19	*HCN2*	11	18	0	1.62E-04	3.17E-07
chr7	*PPP1R17*	11	5	0	1.62E-04	2.67E-02
chr1	*AC253572.1*	10	10	33	3.04E-02	3.15E-02
chr17	*C17orf58*	9	8	0	9.63E-04	2.18E-03
chr16	*ZFPM1*	9	17	0	9.63E-04	7.78E-07
chr21	*PFKL*	8	5	0	2.21E-03	2.67E-02
chr20	*TCFL5*	8	10	0	2.21E-03	3.74E-04
chr19	*CERS1*	7	12	0	4.88E-03	6.35E-05
chr19	*GDF1*	7	12	0	4.88E-03	6.35E-05
chr9	*PRRX2*	7	5	0	4.88E-03	2.67E-02
chr5	*TCF7*	7	11	0	4.88E-03	1.52E-04
chr3	*CDV3*	6	9	0	1.11E-02	8.89E-04
chr19	*LILRA6*	6	4	23	4.23E-02	1.06E-02
chr1	*PPIAL4H*	6	6	1	4.56E-02	4.78E-02
chr1	*RHD*	1	1	14	1.33E-02	1.47E-02
chr4	*UGT2B28*	1	2	14	1.33E-02	4.21E-02

#:No significant difference of gene absence frequencies was found between tumor tissues (Tumor) and normal gastric mucosae (Normal).

*The comparison of gene absence frequencies for each gene between that in the 185 gastric cancer samples and that in 263 individual genomes from SGDP was performed by Fisher's exact test, and the p-values were adjusted by multiple testing corrections (false discovery rate, FDR).

Supplementary Table 14 The 85 significantly different genes between gastric cancer and non-Asian population of SGDP groups*

Gene	Chr.	Tumor Presence	Tumor Absence	SGDP Presence	SGDP Absence	P value	FDR	OR
B3GALT6	chr1	162	23	152	0	6.69E-07	3.84E-06	
FNDC10	chr1	166	19	152	0	1.11E-05	4.79E-05	
PRAMEF14	chr1	169	16	152	0	9.21E-05	3.45E-04	
RHD	chr1	184	1	141	11	1.56E-03	4.41E-03	0.07
GSTM1	chr1	78	107	81	71	4.85E-02	1.11E-01	1.57
AC253572.1	chr1	175	10	131	21	1.27E-02	3.18E-02	0.36
PPIAL4H	chr1	179	6	152	0	3.43E-02	8.04E-02	
OR2T27	chr1	173	12	150	2	2.53E-02	6.16E-02	5.20
POU3F3	chr2	113	72	152	0	8.55E-23	4.17E-21	
C2orf72	chr2	134	51	152	0	2.51E-15	3.77E-14	
CDV3	chr3	176	9	152	0	4.81E-03	1.25E-02	
ZNF718	chr4	70	115	111	41	1.23E-10	1.27E-09	4.45
NAT8L	chr4	168	17	152	0	4.54E-05	1.74E-04	
UGT2B17	chr4	79	106	119	33	3.14E-11	3.41E-10	4.84
UGT2B28	chr4	183	2	139	13	1.00E-03	2.91E-03	0.12
MAP9	chr4	171	14	152	0	1.89E-04	6.60E-04	
FOXD1	chr5	166	19	152	0	1.11E-05	4.79E-05	
TCF7	chr5	174	11	152	0	1.32E-03	3.77E-03	
FOXQ1	chr6	173	12	152	0	6.90E-04	2.07E-03	
HIST1H4B	chr6	130	55	146	6	1.82E-10	1.69E-09	10.29
HIST1H2AB	chr6	120	65	145	7	6.70E-13	7.68E-12	11.22
HIST1H3C	chr6	158	27	152	0	4.15E-08	2.61E-07	
HLA-DQA1	chr6	168	17	115	37	1.78E-04	6.43E-04	0.31
HLA-DPB1	chr6	130	55	138	14	2.77E-06	1.46E-05	4.17
UNCX	chr7	179	6	152	0	3.43E-02	8.04E-02	
AC134684.11	chr8	101	84	118	34	1.16E-05	4.92E-05	2.89
AC134684.8	chr8	42	143	83	69	2.50E-09	2.10E-08	4.10
AC084121.11	chr8	126	59	136	16	1.79E-06	9.71E-06	3.98
AC084121.6	chr8	155	30	152	0	5.17E-09	3.74E-08	
AC084121.12	chr8	157	28	151	1	5.12E-07	3.02E-06	26.93
AC084121.8	chr8	158	27	152	0	4.15E-08	2.61E-07	
AC084121.7	chr8	125	60	135	17	3.70E-06	1.90E-05	3.81
AC084121.5	chr8	176	9	152	0	4.81E-03	1.25E-02	
AC084121.13	chr8	110	75	141	11	4.23E-13	5.50E-12	8.74
ZNF705B	chr8	129	56	143	9	5.17E-09	3.74E-08	6.90
ZNF705D	chr8	166	19	152	0	1.11E-05	4.79E-05	
SCX	chr8	173	12	152	0	6.90E-04	2.07E-03	
AL391987.2	chr9	161	24	150	2	4.28E-05	1.70E-04	11.18

ABO	chr9	179	6	138	14	3.46E-02	8.04E-02	0.33
C10orf143	chr10	166	19	152	0	1.11E-05	4.79E-05	
OR8U1	chr11	162	23	147	5	2.53E-03	6.95E-03	4.17
TRIM64	chr11	66	119	152	0	4.07E-43	7.94E-41	
UBTFL1	chr11	169	16	151	1	6.44E-04	2.06E-03	14.30
TAS2R43	chr12	177	8	136	16	3.33E-02	8.02E-02	0.38
POU4F1	chr13	159	26	152	0	8.30E-08	5.06E-07	
SOX1	chr13	134	51	152	0	2.51E-15	3.77E-14	
ACOT1	chr14	52	133	116	36	4.36E-19	1.06E-17	8.24
ANKRD9	chr14	176	9	152	0	4.81E-03	1.25E-02	
ZFPM1	chr16	168	17	152	0	4.54E-05	1.74E-04	
NATD1	chr17	167	18	152	0	2.24E-05	9.09E-05	
KRTAP9-6	chr17	157	28	95	57	4.37E-06	2.19E-05	0.30
IFI35	chr17	172	13	152	0	3.61E-04	1.19E-03	
C17orf58	chr17	177	8	152	0	9.24E-03	2.34E-02	
MXRA7	chr17	165	20	152	0	5.47E-06	2.60E-05	
HCN2	chr19	167	18	152	0	2.24E-05	9.09E-05	
EFNA2	chr19	163	22	152	0	1.35E-06	7.50E-06	
MEX3D	chr19	158	27	152	0	4.15E-08	2.61E-07	
ONECUT3	chr19	123	62	152	0	2.83E-19	7.88E-18	
KLF16	chr19	118	67	152	0	8.10E-21	2.63E-19	
GDF1	chr19	173	12	152	0	6.90E-04	2.07E-03	
CERS1	chr19	173	12	152	0	6.90E-04	2.07E-03	
GPR42	chr19	146	39	151	1	2.16E-10	1.92E-09	40.34
FGF21	chr19	64	121	132	20	2.63E-23	1.71E-21	12.48
SIGLEC14	chr19	109	76	145	7	2.39E-16	4.66E-15	14.44
ZNF83	chr19	174	11	131	21	1.57E-02	3.88E-02	0.39
LILRA6	chr19	181	4	137	15	3.36E-03	9.09E-03	0.20
GALP	chr19	173	12	151	1	7.83E-03	2.01E-02	10.47
TCF15	chr20	154	31	152	0	2.58E-09	2.10E-08	
AL049634.2	chr20	153	32	98	54	1.58E-04	5.81E-04	0.38
TAF4	chr20	93	92	152	0	9.81E-31	9.57E-29	
TCFL5	chr20	175	10	152	0	2.52E-03	6.95E-03	
AC023490.4	chr22	101	84	147	5	6.08E-21	2.37E-19	24.45
GSC2	chr22	165	20	152	0	5.47E-06	2.60E-05	
NCF4	chr22	172	13	152	0	3.61E-04	1.19E-03	
FAM19A5	chr22	152	33	144	8	3.77E-04	1.23E-03	3.91
PIM3	chr22	119	66	145	7	5.72E-13	6.98E-12	11.49
GAGE12E	chrX	172	13	152	0	3.61E-04	1.19E-03	
AC008162.2	chrX	108	77	131	21	1.83E-08	1.28E-07	4.45
CT45A2	chrX	97	88	142	10	5.82E-18	1.26E-16	12.88
CT45A8	chrX	105	80	134	18	1.62E-10	1.58E-09	5.67
CT45A9	chrX	103	82	142	10	4.81E-16	8.53E-15	11.30
SPANXC	chrX	138	47	147	5	4.87E-09	3.74E-08	10.01

GC2973	26	159	82	70	5.05E-15	7.03E-14	7.16
GC0575	167	18	151	1	1.81E-04	6.43E-04	16.28
GC0643	167	18	150	2	8.64E-04	2.55E-03	8.08

*Note: The comparison of gene absence frequencies for each gene was performed by Fisher's exact test, and the p-values were further corrected by multiple testing corrections (false discovery rate, FDR).

Supplementary Table 15 The 71 significantly different genes between gastric cancer and Asian population of SGDP groups*

Gene	Chr.	Tumor Presence	Tumor Absence	SGDP Presence	SGDP Absence	P value	FDR	OR
B3GALT6	chr1	162	23	111	0	1.71E-05	1.19E-04	
FNDC10	chr1	166	19	111	0	1.14E-04	5.87E-04	
GSTM1	chr1	78	107	63	48	1.65E-02	4.73E-02	1.80
POU3F3	chr2	113	72	111	0	3.15E-18	2.05E-16	
C2orf72	chr2	134	51	111	0	2.48E-12	3.46E-11	
CDV3	chr3	176	9	111	0	1.55E-02	4.52E-02	
ZNF718	chr4	70	115	63	48	1.73E-03	6.49E-03	2.16
NAT8L	chr4	168	17	111	0	3.88E-04	1.72E-03	
MAP9	chr4	171	14	111	0	1.36E-03	5.32E-03	
FOXD1	chr5	166	19	111	0	1.14E-04	5.87E-04	
TCF7	chr5	174	11	111	0	8.09E-03	2.55E-02	
FOXQ1	chr6	173	12	111	0	4.38E-03	1.42E-02	
HIST1H4B	chr6	130	55	110	1	7.81E-12	1.01E-10	46.54
HIST1H2AB	chr6	120	65	110	1	1.65E-14	4.59E-13	59.58
HIST1H3C	chr6	158	27	108	3	6.15E-04	2.67E-03	6.15
HLA-DRB1	chr6	55	130	47	64	3.19E-02	8.75E-02	1.74
HLA-DQA1	chr6	168	17	89	22	1.24E-02	3.83E-02	0.41
HLA-DPB1	chr6	130	55	94	17	5.16E-03	1.65E-02	2.34
AC134684.8	chr8	42	143	55	56	3.29E-06	2.79E-05	3.34
AC084121.11	chr8	126	59	101	10	3.86E-06	3.01E-05	4.73
AC084121.6	chr8	155	30	110	1	5.96E-06	4.47E-05	21.29
AC084121.12	chr8	157	28	109	2	9.79E-05	5.30E-04	9.72
AC084121.8	chr8	158	27	110	1	2.13E-05	1.39E-04	18.80
AC084121.7	chr8	125	60	105	6	8.95E-09	1.09E-07	8.40
AC084121.13	chr8	110	75	86	25	1.52E-03	5.81E-03	2.35
ZNF705B	chr8	129	56	103	8	1.08E-06	1.05E-05	5.59
ZNF705D	chr8	166	19	110	1	1.28E-03	5.08E-03	12.59
SCX	chr8	173	12	111	0	4.38E-03	1.42E-02	
AL391987.2	chr9	161	24	111	0	9.12E-06	6.59E-05	
C10orf143	chr10	166	19	110	1	1.28E-03	5.08E-03	12.59
OR8U1	chr11	162	23	110	1	2.35E-04	1.09E-03	15.62
TRIM64	chr11	66	119	111	0	8.22E-35	1.60E-32	
UBTFL1	chr11	169	16	111	0	7.39E-04	3.13E-03	
POU4F1	chr13	159	26	111	0	2.76E-06	2.45E-05	
SOX1	chr13	134	51	111	0	2.48E-12	3.46E-11	
ACOT1	chr14	52	133	59	52	2.27E-05	1.42E-04	2.90
ANKRD9	chr14	176	9	111	0	1.55E-02	4.52E-02	
ZFPM1	chr16	168	17	111	0	3.88E-04	1.72E-03	
NATD1	chr17	167	18	111	0	2.08E-04	9.91E-04	

KRTAP9-6	chr17	157	28	65	46	8.08E-07	8.29E-06	0.25
IFI35	chr17	172	13	111	0	2.42E-03	8.58E-03	
C17orf58	chr17	177	8	111	0	2.70E-02	7.53E-02	
MXRA7	chr17	165	20	111	0	6.40E-05	3.67E-04	
HCN2	chr19	167	18	111	0	2.08E-04	9.91E-04	
EFNA2	chr19	163	22	111	0	3.30E-05	2.01E-04	
MEX3D	chr19	158	27	111	0	2.69E-06	2.45E-05	
ONECUT3	chr19	123	62	111	0	2.43E-15	9.49E-14	
KLF16	chr19	118	67	111	0	9.26E-17	4.51E-15	
GDF1	chr19	173	12	111	0	4.38E-03	1.42E-02	
CERS1	chr19	173	12	111	0	4.38E-03	1.42E-02	
GPR42	chr19	146	39	108	3	3.57E-06	2.90E-05	9.62
FGF21	chr19	64	121	86	25	5.56E-13	1.08E-11	6.50
SIGLEC14	chr19	109	76	81	30	1.73E-02	4.89E-02	1.88
TCF15	chr20	154	31	111	0	1.97E-07	2.26E-06	
TAF4	chr20	93	92	111	0	1.97E-24	1.93E-22	
TCFL5	chr20	175	10	111	0	1.52E-02	4.52E-02	
AC023490.4	chr22	101	84	105	6	1.15E-14	3.72E-13	14.55
GSC2	chr22	165	20	111	0	6.40E-05	3.67E-04	
NCF4	chr22	172	13	111	0	2.42E-03	8.58E-03	
FAM19A5	chr22	152	33	108	3	6.85E-05	3.82E-04	7.82
PIM3	chr22	119	66	108	3	1.14E-12	1.85E-11	19.97
GAGE12E	chrX	172	13	111	0	2.42E-03	8.58E-03	
AC008162.2	chrX	108	77	96	15	2.87E-07	3.11E-06	4.56
CT45A2	chrX	97	88	101	10	7.64E-13	1.35E-11	9.16
CT45A8	chrX	105	80	105	6	1.35E-13	2.93E-12	13.33
CT45A9	chrX	103	82	105	6	3.00E-14	7.31E-13	13.93
SPANXC	chrX	138	47	104	7	1.99E-05	1.34E-04	5.06
GC2964		74	111	65	46	2.58E-03	8.97E-03	2.12
GC2877		175	10	111	0	1.52E-02	4.52E-02	
GC2973		26	159	34	77	9.21E-04	3.82E-03	2.70
GC0575		167	18	111	0	2.08E-04	9.91E-04	

*Note: The comparison of gene absence frequencies for each gene was performed by Fisher's exact test, and the p-values were further corrected by multiple testing corrections (false discovery rate, FDR).

Supplementary Table 16 The result of functional enrichment analysis of 186 distributed genes on GRCh38 using Metascape

Database	Description	LogP	Genes
GO BP	detection of stimulus	-9.06	*OPN1MW, HLA-DRB1, OPN1LW, OR52N1, OR4P4, OR5P2, OR2T10, OR2T11, OR8U1, OR4C11, OR4S2, TAS2R43, OR4N4, OR52N5, OR4M2, OR51A2, OR2T27, OR4F21, OPN1MW2, OPN1MW3*
GO BP	MHC class II protein complex assembly	-7.71	*HLA-DPB1, HLA-DQA1, HLA-DQB1, HLA-DRB1, HLA-DRB5, KIR2DL3, FCGR3B, NCF4, KLRC2, GBP3, TRIM48, H2AC4, H4C2, IFI35, AZU1, PTK2B, ZFPM1, PSG9, BTNL3, LILRB3, LILRA6, TCF7, TJP1, ZNF83, ZNF718, ZNF705D*
GO BP	protein-chromophore linkage	-5.94	*OPN1MW, OPN1LW, OPN1MW2, OPN1MW3, CERS1, MAP9, SCX*
GO BP	protein deubiquitination	-4.71	*TAF4, USP17L2, USP17L8, USP17L7, USP17L1, USP17L3, USP17L10, H2AC4*
GO BP	snRNA 3'-end processing	-4.61	*CT45A2, CT45A10, CT45A9, CT45A8*
GO BP	regulation of myeloid cell differentiation	-2.95	*PTK2B, HLA-DRB1, POU4F1, H4C2, LILRB3, ZFPM1, PSG9, TCF7, TCF15*
GO BP	defense response to Gram-negative bacterium	-2.90	*AZU1, GALP, LCE3B, LCE3C*
Reactome	Regulation of beta-cell development	-2.83	*INSM1, MAFA, ONECUT3*
GO BP	cellular response to toxic substance	-2.45	*GSTM1, HBG1, HP, CERS1*
KEGG	Neutrophil extracellular trap formation	-2.39	*AZU1, FCGR3B, NCF4, H2AC4, H4C2, COPRS*
GO BP	negative regulation of protein transport	-2.30	*PFKL, PKDCC, USP17L2, PIM3*
KEGG	Osteoclast differentiation	-2.26	*FCGR3B, NCF4, LILRB3, LILRA6*
KEGG	Natural killer cell mediated cytotoxicity	-2.22	*PTK2B, FCGR3B, KIR2DL3, KLRC2*
KEGG	Chemical carcinogenesis & Retinol metabolism	-2.20	*UGT2B17, UGT2B28, DHRS4L2, GSTM1*
GO BP	acute inflammatory response	-2.08	*HP, HPR, MRGPRX1*
GO BP	response to glucose	-2.07	*PTK2B, PFKL, FGF21, MAFA*

Supplementary Table 17 The 203 genes identified as absence by PAV method but as presence by SV methods in at least one tumor sample

Gene	Chromosome	Begin	End	(-/-)*	(+/+)*	(-/+)*	(+/-)*
B3GALT6	chr1	1232226	1235041	0	162	0	23
VWA1	chr1	1434861	1442882	0	182	0	3
FNDC10	chr1	1598012	1600135	0	166	0	19
PRAMEF1	chr1	12791397	12796628	0	184	0	1
PRAMEF11	chr1	12824605	12831410	0	184	0	1
PRAMEF4	chr1	12879224	12886201	0	184	0	1
PRAMEF13	chr1	13196330	13201409	0	178	0	7
PRAMEF14	chr1	13342034	13347134	0	169	0	16
RHD	chr1	25272393	25330445	0	184	0	1
POU3F1	chr1	38044611	38046794	0	183	0	2
GBP3	chr1	89006679	89022894	0	175	1	9
DNTTIP2	chr1	93866284	93879918	0	181	1	3
GSTM1	chr1	109687814	109709039	0	78	0	107
AC253572.1	chr1	120723946	120793874	0	175	0	10
HIST2H3PS2	chr1	143905556	143905966	0	183	0	2
PPIAL4H	chr1	146344131	146344790	0	179	0	6
LCE3C	chr1	152600662	152601086	0	116	0	69
LCE3B	chr1	152613811	152614098	0	116	0	69
FCGR3B	chr1	161623196	161631963	0	184	0	1
OR2T10	chr1	248590487	248597700	0	172	0	13
OR2T11	chr1	248623557	248635091	0	172	0	13
OR2T27	chr1	248649838	248655528	0	173	0	12
CDV3	chr3	133573730	133590261	0	176	0	9
TMEM271	chr4	573880	576300	0	181	0	4
NAT8L	chr4	2059512	2069089	0	168	0	17
FAM90A26	chr4	9170409	9176730	0	39	0	146
USP17L10	chr4	9210657	9212608	0	25	0	160
USP17L13	chr4	9224896	9226847	0	184	0	1
USP17L23	chr4	9272364	9272914	0	184	0	1
UGT2B17	chr4	68537184	68568527	0	79	0	106
UGT2B28	chr4	69280499	69295050	0	183	0	2
MAP9	chr4	155342658	155376970	0	171	0	14
TAF11L2	chr5	17498231	17498827	0	184	0	1
FOXD1	chr5	73444827	73448777	0	166	0	19
TCF7	chr5	134114681	134151865	0	174	0	11
PCDHA8	chr5	140841187	141012347	0	184	0	1
NEURL1B	chr5	172641266	172691540	0	184	0	1
BTNL3	chr5	180988845	181006727	0	167	0	18
FOXQ1	chr6	1312098	1314758	0	173	0	12
HIST1H4B	chr6	26026815	26027252	0	130	0	55
HIST1H2AB	chr6	26033176	26033568	0	120	0	65

HIST1H3C	chr6	26045411	26045821	0	158	0	27
MUC22	chr6	31010474	31035402	0	182	1	2
HLA-DRB5	chr6	32517353	32530287	1	50	0	134
HLA-DRB1	chr6	32578769	32589848	1	55	0	129
HLA-DQA1	chr6	32628179	32647062	0	168	0	17
HLA-DQB1	chr6	32659467	32668383	1	72	0	112
HLA-DPB1	chr6	33075990	33089696	0	129	1	55
MARCKS	chr6	113857345	113863475	0	183	1	1
UNCX	chr7	1232872	1237326	0	179	0	6
HOXA13	chr7	27193503	27200106	0	184	0	1
ZNRF2	chr7	30284597	30367689	0	182	0	3
PPP1R17	chr7	31687215	31708455	0	180	0	5
ZNF138	chr7	64794388	64833681	0	179	0	6
OR4F21	chr8	166049	167043	0	184	0	1
USP17L1	chr8	7332387	7333979	0	183	0	2
AC134684.11	chr8	7556700	7559712	0	101	0	84
AC134684.8	chr8	7571995	7575006	0	42	0	143
AC134684.9	chr8	7579644	7582653	0	184	0	1
AC084121.11	chr8	7723091	7726101	0	126	0	59
AC084121.6	chr8	7730739	7733749	0	155	0	30
AC084121.12	chr8	7738387	7741396	0	157	0	28
AC084121.8	chr8	7746034	7749044	0	158	0	27
AC084121.7	chr8	7753682	7756692	0	125	0	60
AC084121.5	chr8	7761330	7764340	0	176	0	9
AC084121.13	chr8	7768977	7771988	0	110	0	75
ZNF705B	chr8	7926337	7952413	0	129	0	56
USP17L8	chr8	7971661	7973253	0	78	0	107
USP17L3	chr8	7976393	7977985	0	180	0	5
ZNF705D	chr8	12104389	12115516	0	166	0	19
USP17L7	chr8	12132417	12134438	0	162	0	23
USP17L2	chr8	12137168	12139077	0	168	0	17
PTK2B	chr8	27311482	27459391	0	182	0	3
MAFA	chr8	143419182	143430406	0	182	0	3
SCX	chr8	144266453	144268481	0	173	0	12
WASHC1	chr9	14475	73865	0	183	0	2
RLN1	chr9	5334930	5339876	0	184	0	1
SPATA31A6	chr9	42183659	42189882	0	180	0	5
AL391987.2	chr9	61861625	61863200	0	161	0	24
PRRX2	chr9	129665647	129722674	0	180	0	5
ABO	chr9	133250401	133276024	0	179	0	6
C10orf143	chr10	130020025	130110830	0	166	0	19
SPRN	chr10	133420666	133424572	0	184	0	1
BET1L	chr11	167784	207428	0	184	0	1
SSU72P5	chr11	4233288	4233872	0	184	0	1

SSU72P2	chr11	4242056	4242640	0	184	0	1
OR51A2	chr11	4954772	4955713	0	167	0	18
HBG1	chr11	5248079	5249859	0	181	0	4
OR52N5	chr11	5776165	5783355	0	176	0	9
OR52N1	chr11	5786471	5791265	0	182	0	3
OR5P2	chr11	7795905	7796973	0	182	0	3
MRGPRX1	chr11	18933813	18939507	0	184	0	1
OR4C11	chr11	55602360	55607645	0	158	0	27
OR4P4	chr11	55635113	55640309	0	158	0	27
OR4S2	chr11	55648327	55652854	0	158	0	27
OR8U1	chr11	56375624	56376553	0	162	0	23
TRIM64	chr11	89968502	89974072	0	66	0	119
UBTFL1	chr11	90085950	90087131	0	169	0	16
KLRC2	chr12	10426854	10442300	0	172	0	13
TAS2R43	chr12	11091287	11092313	0	177	0	8
BRI3BP	chr12	124993645	125031231	0	184	0	1
POU4F1	chr13	78598362	78603552	0	159	0	26
SOX1	chr13	112067647	112070488	0	134	0	51
DHRS4L2	chr14	23969874	24006408	0	184	0	1
FOXG1	chr14	28760330	28770277	0	184	0	1
ACOT1	chr14	73537143	73543796	0	52	0	133
ANKRD9	chr14	102501767	102509799	0	176	0	9
AC134980.3	chr15	22015233	22095857	0	181	0	4
OR4M2	chr15	22070241	22083221	0	178	0	7
OR4N4	chr15	22094522	22095472	0	181	0	4
TJP1	chr15	29699367	29968865	0	184	0	1
HS3ST4	chr16	25691959	26137685	0	184	0	1
TP53TG3	chr16	32673528	32676732	0	184	0	1
TP53TG3F	chr16	33459045	33462249	0	184	0	1
FOXC2	chr16	86566829	86569728	0	184	0	1
ZFPM1	chr16	88453280	88537031	0	168	0	17
BHLHA9	chr17	1270559	1271460	0	183	0	2
NATD1	chr17	21238870	21253410	0	167	0	18
COPRS	chr17	31851871	31859291	0	181	0	4
TBC1D3F	chr17	36428618	36439566	0	184	0	1
KRTAP9-6	chr17	41265339	41266641	0	157	0	28
IFI35	chr17	43006725	43014456	0	172	0	13
FAM171A2	chr17	44353215	44363875	0	184	0	1
LRRC37A	chr17	46292733	46337794	0	181	0	4
ARL17A	chr17	46516702	46579682	0	183	0	2
C17orf58	chr17	67991101	67996431	0	177	0	8
MXRA7	chr17	76672551	76711016	0	165	0	20
RAB12	chr18	8609437	8639382	0	184	0	1
KCNG2	chr18	79863668	79900184	0	180	0	5

HCN2	chr19	589881	617159	0	167	0	18
PLPPR3	chr19	812488	821977	0	182	0	3
AZU1	chr19	825097	832018	0	179	0	6
EFNA2	chr19	1285873	1301431	0	163	0	22
MEX3D	chr19	1554669	1568058	0	158	0	27
ONECUT3	chr19	1752373	1780988	0	123	0	62
KLF16	chr19	1852399	1863579	0	118	0	67
SAMD1	chr19	14087840	14091036	0	184	0	1
CERS1	chr19	18868545	18896727	0	173	0	12
GDF1	chr19	18868545	18896158	0	173	0	12
AC008554.1	chr19	20535825	20565809	0	184	0	1
WTIP	chr19	34481638	34512304	0	184	0	1
GPR42	chr19	35370929	35372962	0	146	0	39
PSG4	chr19	43192702	43207299	0	170	0	15
PSG9	chr19	43211791	43269530	0	168	0	17
CD177	chr19	43353686	43363172	0	184	0	1
DACT3	chr19	46647551	46661182	0	180	0	5
GRIN2D	chr19	48394875	48444931	0	183	0	2
FGF21	chr19	48755559	48758333	0	64	0	121
SIGLEC14	chr19	51642553	51646801	0	109	0	76
ZNF83	chr19	52594060	52690496	0	174	0	11
LILRB3	chr19	54216278	54223506	0	181	0	4
LILRA6	chr19	54236592	54242791	0	181	0	4
KIR2DL3	chr19	54738515	54753052	0	184	0	1
KIR3DL1	chr19	54816468	54830778	0	184	0	1
TMEM238	chr19	55379245	55384598	0	180	0	5
GALP	chr19	56176020	56185775	0	173	0	12
CYS1	chr2	10056780	10080944	0	181	0	4
PKDCC	chr2	42048021	42058517	0	184	0	1
FOXI3	chr2	88446787	88452656	0	184	0	1
POU3F3	chr2	104855511	104858574	0	113	0	72
SMIM39	chr2	131035092	131035262	0	184	0	1
HOXD11	chr2	176104216	176109754	0	184	0	1
C2orf72	chr2	231037523	231049719	0	134	0	51
AQP12A	chr2	240691845	240698483	0	182	0	3
TCF15	chr20	603797	610398	0	154	0	31
AL049634.2	chr20	1540144	1620009	0	153	0	32
INSM1	chr20	20368104	20370949	0	184	0	1
NKX2-4	chr20	21395365	21397526	0	184	0	1
TAF4	chr20	61953469	62065810	0	93	0	92
TCFL5	chr20	62841115	62861763	0	175	0	10
U2AF1L5	chr21	6484623	6499261	0	183	0	2
KCNE1B	chr21	7816675	7829926	0	177	0	8
PWP2	chr21	44107373	44131181	0	184	0	1

GATD3A	chr21	44133610	44210114	0	181	0	4
PFKL	chr21	44300051	44327376	0	180	0	5
POTEH	chr22	15690026	15721631	0	183	0	2
AC023490.4	chr22	18633984	18634682	0	101	0	84
GSC2	chr22	19148576	19150283	0	165	0	20
TBX1	chr22	19756703	19783593	0	184	0	1
AP000552.3	chr22	21360601	21361299	0	184	0	1
NCF4	chr22	36860988	36878017	0	172	0	13
GALR3	chr22	37823382	37825495	0	184	0	1
APOBEC3B	chr22	38982347	38992804	0	162	0	23
APOBEC3F	chr22	39040604	39055972	0	184	0	1
FAM19A5	chr22	48489460	48850912	0	152	0	33
PIM3	chr22	49960768	49964072	0	119	0	66
GAGE12E	chrX	49551333	49558649	0	172	0	13
GAGE12H	chrX	49579983	49587301	0	184	0	1
DGAT2L6	chrX	70177483	70205545	0	184	0	1
RHOXF2B	chrX	120070672	120077705	0	183	0	2
AC008162.2	chrX	120877496	120878924	0	108	0	77
CT47A6	chrX	120958165	120961487	0	182	0	3
ACTRT1	chrX	128050962	128052398	0	184	0	1
CT45A2	chrX	135811668	135820012	0	97	0	88
CT45A8	chrX	135846499	135854538	0	105	0	80
CT45A9	chrX	135863776	135871812	0	103	0	82
CT45A10	chrX	135881063	135889086	0	183	0	2
SPANXC	chrX	141241463	141242496	0	138	0	47
MAGEA3	chrX	152698767	152702347	0	184	0	1
OPN1LW	chrX	154144224	154159032	0	184	0	1
OPN1MW	chrX	154182596	154196135	0	183	0	2
OPN1MW2	chrX	154219756	154233286	0	184	0	1
OPN1MW3	chrX	154257538	154271805	0	184	0	1

(*) gene PAV classification patterns

(-/-) for genes identified as absence by both SV method and PAV method.

(+/+) for genes identified as presence by both SV method and PAV method.

(-/+) for genes identified as absence by SV method but as presence by PAV method.

(+/-) for genes identified as absence by PAV method but as presence by SV method.

Supplementary Table 18 Differentially expressed genes between *GC0643* absence and presence groups*

Gene Name	FC	Log2FC	P value	FDR	change
ALB	90.3510	6.50	3.47E-06	5.02E-04	Up
RNU1-27P	55.1211	5.78	6.50E-07	1.17E-04	Up
RNVU1-7	48.5095	5.60	1.80E-05	2.09E-03	Up
FGG	33.2111	5.05	1.45E-05	1.76E-03	Up
AL137798.2	33.0100	5.04	1.77E-05	2.07E-03	Up
FGB	32.4794	5.02	9.61E-06	1.21E-03	Up
VIP	23.0683	4.53	1.36E-07	2.87E-05	Up
AFP	21.9579	4.46	1.94E-05	2.23E-03	Up
CARTPT	19.6961	4.30	1.71E-05	2.01E-03	Up
VTN	9.3189	3.22	3.94E-05	4.05E-03	Up
CAPNS2	0.1249	-3.00	2.18E-11	9.94E-09	Down
PRR4	0.1245	-3.01	4.05E-13	2.67E-10	Down
SERPINB10	0.1233	-3.02	7.93E-07	1.39E-04	Down
TMEM40	0.1231	-3.02	8.26E-09	2.20E-06	Down
ATP4B	0.1225	-3.03	9.27E-07	1.59E-04	Down
KLK13	0.1219	-3.04	2.19E-08	5.26E-06	Down
CYP3A4	0.1218	-3.04	8.11E-08	1.75E-05	Down
SERPINB2	0.1217	-3.04	1.02E-06	1.71E-04	Down
TRARG1	0.1210	-3.05	1.66E-10	6.24E-08	Down
CIDEA	0.1204	-3.05	1.97E-08	4.87E-06	Down
CES1	0.1202	-3.06	1.88E-09	5.61E-07	Down
CIDEC	0.1180	-3.08	1.67E-09	5.04E-07	Down
LY6G6D	0.1177	-3.09	9.51E-09	2.49E-06	Down
C10orf99	0.1134	-3.14	2.52E-06	3.81E-04	Down
CALM2P1	0.1112	-3.17	3.15E-06	4.66E-04	Down
ATP4A	0.1106	-3.18	1.23E-06	2.00E-04	Down
C3orf85	0.1087	-3.20	1.33E-08	3.38E-06	Down
FTLP10	0.1081	-3.21	1.30E-10	4.92E-08	Down
MS4A10	0.1079	-3.21	2.08E-11	9.69E-09	Down
GIP	0.1065	-3.23	1.57E-09	4.76E-07	Down
FLG	0.1059	-3.24	1.02E-14	8.00E-12	Down
RN7SKP193	0.1037	-3.27	4.38E-06	6.16E-04	Down
AC083902.1	0.1037	-3.27	2.12E-08	5.13E-06	Down
AC107302.1	0.1022	-3.29	4.82E-08	1.10E-05	Down
ZG16	0.1021	-3.29	9.51E-12	4.72E-09	Down
AC006262.3	0.1020	-3.29	8.03E-08	1.75E-05	Down
TMPRSS11D	0.1010	-3.31	4.01E-11	1.70E-08	Down
KRT126P	0.0995	-3.33	8.65E-10	2.78E-07	Down
ENDOU	0.0993	-3.33	5.93E-16	6.48E-13	Down
ARSFP1	0.0990	-3.34	1.62E-07	3.32E-05	Down
CALML3-AS1	0.0975	-3.36	4.52E-13	2.88E-10	Down

CWH43	0.0969	-3.37	5.70E-08	1.29E-05	Down
AC084357.3	0.0968	-3.37	6.47E-11	2.61E-08	Down
PGA4	0.0956	-3.39	4.82E-05	4.80E-03	Down
MUC22	0.0954	-3.39	7.15E-11	2.85E-08	Down
DSC3	0.0936	-3.42	1.16E-12	6.72E-10	Down
PRSS56	0.0934	-3.42	8.43E-12	4.24E-09	Down
RNVU1-18	0.0932	-3.42	4.45E-09	1.23E-06	Down
IGFL1	0.0914	-3.45	5.49E-09	1.50E-06	Down
CBLIF	0.0910	-3.46	2.01E-10	7.39E-08	Down
MTTP	0.0886	-3.50	7.37E-10	2.39E-07	Down
TMPRSS11F	0.0884	-3.50	6.97E-10	2.28E-07	Down
MME	0.0882	-3.50	1.07E-14	8.17E-12	Down
PKP1	0.0878	-3.51	2.14E-12	1.17E-09	Down
DSG1-AS1	0.0859	-3.54	1.68E-11	7.93E-09	Down
GJB6	0.0858	-3.54	9.13E-09	2.41E-06	Down
CLEC2A	0.0850	-3.56	4.18E-10	1.44E-07	Down
PADI1	0.0850	-3.56	4.51E-11	1.87E-08	Down
DAPL1	0.0845	-3.56	5.54E-15	4.71E-12	Down
CYSRT1	0.0842	-3.57	6.72E-13	4.08E-10	Down
AL356387.1	0.0830	-3.59	5.80E-07	1.06E-04	Down
RNA5-8SN3	0.0829	-3.59	3.72E-11	1.62E-08	Down
MME-AS1	0.0828	-3.59	2.21E-15	2.01E-12	Down
KRT6C	0.0827	-3.60	1.26E-06	2.04E-04	Down
CLCA4-AS1	0.0819	-3.61	2.27E-15	2.02E-12	Down
STATH	0.0778	-3.68	5.81E-10	1.92E-07	Down
NCCRP1	0.0765	-3.71	5.10E-11	2.10E-08	Down
CAPN14	0.0759	-3.72	5.46E-13	3.42E-10	Down
AL136982.6	0.0744	-3.75	1.89E-15	1.76E-12	Down
CLCA2	0.0717	-3.80	2.06E-07	4.14E-05	Down
TMPRSS11BNL	0.0716	-3.80	5.22E-11	2.12E-08	Down
IVL	0.0706	-3.82	1.08E-09	3.43E-07	Down
AC093524.1	0.0706	-3.82	1.28E-10	4.91E-08	Down
CYP4F29P	0.0697	-3.84	1.15E-16	1.38E-13	Down
UGT2B4	0.0697	-3.84	3.82E-12	2.00E-09	Down
DEFB4A	0.0694	-3.85	6.79E-09	1.84E-06	Down
KRT79	0.0687	-3.86	6.68E-16	7.10E-13	Down
DSG3	0.0681	-3.88	1.07E-08	2.78E-06	Down
HCG22	0.0677	-3.88	1.26E-14	9.30E-12	Down
PAGE1	0.0673	-3.89	3.93E-11	1.69E-08	Down
TGM1	0.0663	-3.92	1.31E-19	2.18E-16	Down
UCP1	0.0642	-3.96	1.52E-15	1.53E-12	Down
GBA3	0.0623	-4.00	7.45E-14	5.28E-11	Down
KRT15	0.0611	-4.03	9.31E-15	7.42E-12	Down
FABP2	0.0591	-4.08	7.26E-11	2.86E-08	Down

ALDOB	0.0565	-4.15	3.72E-13	2.50E-10	Down
FABP4	0.0547	-4.19	1.20E-10	4.62E-08	Down
PGA5	0.0544	-4.20	2.37E-07	4.64E-05	Down
EPGN	0.0535	-4.22	2.00E-17	2.63E-14	Down
PLIN1	0.0507	-4.30	3.63E-17	4.62E-14	Down
SPRR1A	0.0507	-4.30	4.07E-09	1.17E-06	Down
TMPRSS15	0.0504	-4.31	4.65E-10	1.55E-07	Down
RBP2	0.0481	-4.38	1.78E-15	1.75E-12	Down
LGALS7	0.0475	-4.40	1.14E-09	3.54E-07	Down
SBSN	0.0451	-4.47	8.58E-16	8.87E-13	Down
MIR6510	0.0450	-4.47	1.36E-13	9.45E-11	Down
TMPRSS11A	0.0444	-4.49	3.73E-18	5.10E-15	Down
SPRR2E	0.0443	-4.50	3.13E-12	1.66E-09	Down
S100A9	0.0435	-4.52	1.46E-11	7.00E-09	Down
BPIFB2	0.0415	-4.59	4.34E-13	2.82E-10	Down
SPRR2B	0.0399	-4.65	1.25E-14	9.30E-12	Down
SCGB3A1	0.0387	-4.69	1.86E-15	1.76E-12	Down
MEP1B	0.0384	-4.70	1.05E-21	2.50E-18	Down
SPINK5	0.0368	-4.76	2.10E-12	1.16E-09	Down
SPRR1B	0.0367	-4.77	4.34E-10	1.47E-07	Down
SCEL	0.0345	-4.86	2.93E-11	1.30E-08	Down
ADH7	0.0343	-4.87	9.89E-10	3.15E-07	Down
KRT3	0.0337	-4.89	1.98E-21	3.79E-18	Down
THRSP	0.0333	-4.91	7.90E-23	2.52E-19	Down
RNA5-8SN1	0.0327	-4.94	1.49E-14	1.08E-11	Down
SERPINB3	0.0312	-5.00	2.29E-08	5.47E-06	Down
RNASE7	0.0300	-5.06	3.32E-19	4.88E-16	Down
DSG1	0.0300	-5.06	1.54E-19	2.45E-16	Down
CRISP3	0.0295	-5.08	4.37E-09	1.23E-06	Down
ADIPOQ-AS1	0.0267	-5.22	4.25E-15	3.69E-12	Down
SERPINB11	0.0265	-5.24	7.68E-12	3.92E-09	Down
CLCA1	0.0262	-5.25	1.84E-10	6.84E-08	Down
ADIPOQ	0.0249	-5.33	8.18E-15	6.66E-12	Down
SERPINB13	0.0247	-5.34	6.67E-15	5.55E-12	Down
SCEL-AS1	0.0234	-5.42	1.16E-12	6.72E-10	Down
CNFN	0.0230	-5.44	5.70E-23	1.98E-19	Down
SPRR2F	0.0212	-5.56	2.36E-22	6.57E-19	Down
KRT78	0.0203	-5.62	6.72E-22	1.71E-18	Down
CLCA4	0.0167	-5.90	4.49E-16	5.05E-13	Down
SPINK7	0.0162	-5.95	4.60E-26	3.52E-22	Down
CALML3	0.0156	-6.00	1.64E-21	3.31E-18	Down
S100A8	0.0155	-6.01	6.74E-17	8.31E-14	Down
MAL	0.0149	-6.07	1.44E-21	3.05E-18	Down
SLURP1	0.0134	-6.22	1.51E-23	7.23E-20	Down

SPRR2A	0.0132	-6.25	2.70E-16	3.13E-13	Down
GBP6	0.0121	-6.37	2.25E-24	1.23E-20	Down
SPRR2D	0.0116	-6.42	2.40E-22	6.57E-19	Down
CSTA	0.0115	-6.44	3.88E-21	6.74E-18	Down
FAM25A	0.0113	-6.47	2.09E-19	3.19E-16	Down
A2ML1	0.0107	-6.55	1.84E-24	1.17E-20	Down
KRT13	0.0079	-6.99	3.39E-13	2.32E-10	Down
MUC21	0.0076	-7.04	1.27E-21	2.85E-18	Down
TMPRSS11E	0.0075	-7.06	4.01E-23	1.70E-19	Down
AC019349.1	0.0068	-7.20	1.39E-11	6.72E-09	Down
IL36A	0.0065	-7.27	2.67E-21	4.86E-18	Down
CRCT1	0.0065	-7.27	5.37E-19	7.60E-16	Down
KRT4	0.0061	-7.36	4.04E-12	2.09E-09	Down
SPRR3	0.0060	-7.38	2.05E-12	1.15E-09	Down
AL121899.2	0.0052	-7.59	1.17E-30	2.88E-26	Down
TGM3	0.0047	-7.72	2.26E-30	2.88E-26	Down
RHCG	0.0034	-8.20	1.52E-30	2.88E-26	Down
TMPRSS11B	0.0024	-8.71	5.13E-23	1.96E-19	Down
CRNN	0.0013	-9.58	3.85E-29	3.68E-25	Down

* The significance of difference was tested by likelihood ratio test using limma (version 3.46.0) and edgeR (version 3.32.1), and the p-values were adjusted by multiple testing corrections (false discovery rate, FDR).

Supplementary Table 19 The upstream regulators of differential genes based on *GC0643* absence variation

**Regulator**	**Molecular type**	**Activation statue**	**Activation z-score**	**P-value**	**Target genes in datasets**
FOXC1	Transcription factor	Activated	3.45	1.02E-12	CSTA, DSG1, DSG3, IVL, KRT6C, KRT79, SPINK5, SPRR1B, SPRR2A, SPRR2D
KMT2D	Transcription factor	Activated	2.99	9.62E-12	DSC3, DSG3, IVL, KRT6C, S100A8, SPRR1A, SPRR1B, TGM1,VTN
IgG	Complex	Activated	2.449	4.81E-04	DSG3, IVL, KRT13, KRT15, LGALS7/LGALS7B, SERPINB2
EHF	Transcription factor	Inhibited	-3.162	3.43E-11	CNFN, RHCG, S100A8, S100A9, SCEL, SPRR1B, SPRR2A, SPRR2D, SPRR2E, SPRR3
ROCK2	Kinase	Inhibited	-2.63	1.03E-09	DSG1, DSG3, IVL, S100A8, SCEL, SPINK5, SPRR3
EFNA4	Kinase	Inhibited	-2.63	3.24E-09	DSG1, IVL, KRT13, KRT4, PKP1, SPRR3, TGM1
GLIS1	Transcription factor	Inhibited	-2	4.60E-09	IVL, S100A9, SPRR1B, TGM1
Ca2+	Chemical	Inhibited	-2.608	3.37E-08	FLG, GIP, IVL, PADI1, S100A9, SBSN, SERPINB2, SPRR2A, TGM1, VIP
EFNA3	Kinase	Inhibited	-2.449	1.37E-07	DSG1, KRT13, KRT4, PKP1, SPRR3, TGM1
EFNA1	Other	Inhibited	-2.433	1.84E-06	DSG1, IVL, KRT13, KRT4, PKP1,TGM1
EFNA5	Kinase	Inhibited	-2.236	4.05E-06	DSG1, KRT13, KRT4, PKP1,TGM1
EFNA2	Kinase	Inhibited	-2.236	1.39E-05	DSG1, KRT13, KRT4, PKP1, TGM1
Nicotine	Chemical drug	Inhibited	-2.164	2.06E-05	CARTPT, CYP3A4, DEFB4A/DEFB4B, FLG, IVL, TGM1, UCP1
Tretinoin	Chemical -endogenous mammalian	Inhibited	-2.049	5.94E-04	AFP, CALML3, CES1, DSG1, FGG, IVL, KRT15, KRT4, PGA5 (includes others), RBP2
Methylpred-nisolone	Chemical drug	Inhibited	-2.433	6.79E-04	DSG1, DSG3, IVL, KRT15, PKP1, SCEL
Rosiglitazone	Chemical drug	Inhibited	-2.573	7.11E-04	ADIPOQ, CIDEA, CYP3A4, FABP4, S100A8, S100A9, UCP1
FOXO1	Transcription factor	Inhibited	-2.406	8.21E-04	ADIPOQ, CIDEA, DEFB4A/DEFB4B, FABP4, MTTP, SERPINB2

Supplementary Table 20 The differential genes set based on *GC0643* overexpression in HGC27 cells*

Gene Name	Fold Change	*P* Value	Change
EIF3CL	4.16	1.51E-03	Up
BOLA2B	2.53	6.83E-03	Up
ALOXE3	2.29	1.55E-02	Up
TMEM191C	2.19	4.55E-03	Up
TRIM72	2.11	3.57E-03	Up
TINAGL1	2.08	3.32E-03	Up
RAPGEF3	2.02	1.65E-04	Up
CGB1	2.00	2.00E-02	Up
DLGAP3	1.98	7.69E-03	Up
NCBP2L	1.98	3.23E-02	Up
PIP5KL1	1.92	2.49E-04	Up
PLPP2	1.92	2.26E-02	Up
MAPK8IP2	1.91	3.41E-04	Up
MGAT5B	1.91	2.06E-02	Up
LDLRAD4	1.90	3.18E-03	Up
XAGE1B	1.86	4.29E-02	Up
IL23A	1.86	2.38E-02	Up
SH2D6	1.84	2.56E-02	Up
AKR7A3	1.83	3.17E-02	Up
FBXO44	1.79	2.96E-02	Up
VEGFC	1.79	1.68E-02	Up
HAPLN2	1.73	3.61E-02	Up
TMEM217	1.72	1.68E-02	Up
PANX2	1.72	4.63E-03	Up
SLC25A48	1.71	2.72E-02	Up
PCK2	1.70	3.84E-02	Up
C6orf226	1.69	1.69E-02	Up
PAOX	1.68	1.47E-02	Up
COX7A1	1.68	3.75E-02	Up
TAS1R1	1.68	3.43E-02	Up
CYP26A1	1.67	3.24E-02	Up
PPCDC	1.67	1.16E-03	Up
C16orf71	1.67	1.42E-02	Up
ESR2	1.67	3.24E-02	Up
RADIL	1.67	2.99E-02	Up
TSGA10	1.66	2.98E-02	Up
C8G	1.65	4.68E-02	Up
UPP1	1.62	1.88E-02	Up
EVX1	1.61	2.77E-02	Up
PLEKHD1	1.60	4.09E-02	Up
CAMK2N2	1.60	1.25E-02	Up

NUPR1	1.59	9.07E-03	Up
ID3	1.58	2.84E-03	Up
WNT3A	1.58	2.91E-02	Up
ICOSLG	1.57	4.20E-02	Up
HEXIM2	1.57	1.75E-03	Up
HIST1H2BJ	1.56	1.05E-02	Up
NEURL2	1.56	2.33E-02	Up
TTC25	1.56	4.96E-02	Up
DHRS3	1.56	5.49E-03	Up
GGT1	1.55	2.22E-03	Up
HBA2	1.55	7.83E-04	Up
HIST1H2AM	1.55	4.78E-02	Up
C22orf15	1.55	3.66E-02	Up
AC068234.1	1.55	2.34E-02	Up
LRRC56	1.55	4.75E-02	Up
HIST1H1C	1.53	8.23E-04	Up
LIPE	1.53	3.48E-02	Up
SLC6A9	1.51	2.34E-03	Up
HBA1	1.50	2.24E-03	Up
AC099850.2	-1.50	1.81E-03	Down
BNIP3L	-1.50	2.00E-03	Down
METTL15	-1.50	4.02E-03	Down
ATF2	-1.50	1.20E-03	Down
SPTY2D1	-1.50	4.14E-03	Down
CXorf38	-1.50	5.15E-03	Down
FSD2	-1.50	3.77E-02	Down
SCP2	-1.50	2.40E-03	Down
STRN3	-1.50	6.64E-03	Down
CCNJ	-1.50	6.43E-03	Down
DICER1	-1.50	8.67E-03	Down
SLC1A1	-1.51	1.47E-02	Down
NUP35	-1.51	1.93E-03	Down
TAB3	-1.51	1.25E-02	Down
RAB8B	-1.51	2.27E-03	Down
MBIP	-1.51	3.07E-03	Down
LIMS1	-1.51	2.15E-03	Down
GPAM	-1.51	5.39E-03	Down
ZNF10	-1.51	3.04E-02	Down
CARNMT1	-1.51	9.31E-03	Down
DAAM1	-1.51	4.94E-03	Down
IL20RB	-1.51	2.57E-02	Down
TRIM59	-1.51	1.05E-03	Down
CHML	-1.51	3.34E-04	Down
LIN52	-1.51	5.00E-03	Down

TRMT13	-1.52	1.63E-03	Down
OPN3	-1.52	1.03E-03	Down
NSUN6	-1.52	2.81E-03	Down
MNS1	-1.52	1.51E-02	Down
TMEFF1	-1.52	2.19E-03	Down
CASP4	-1.52	2.14E-02	Down
CHMP5	-1.52	1.42E-03	Down
HAT1	-1.52	2.38E-03	Down
F2R	-1.52	7.45E-04	Down
SNRNP48	-1.52	7.88E-04	Down
CEP76	-1.52	5.58E-03	Down
TMF1	-1.52	5.10E-03	Down
CENPC	-1.52	3.55E-03	Down
PXYLP1	-1.53	8.20E-03	Down
PUS7	-1.53	3.22E-04	Down
TXNDC9	-1.53	7.31E-04	Down
FAM162A	-1.53	2.37E-03	Down
TNFRSF19	-1.53	5.02E-03	Down
DCUN1D1	-1.53	7.28E-04	Down
ERLIN2	-1.53	1.36E-03	Down
VRK1	-1.53	7.54E-04	Down
FKBP7	-1.53	2.36E-03	Down
PROS1	-1.53	3.08E-03	Down
PM20D2	-1.53	2.28E-03	Down
ZNF432	-1.53	1.51E-02	Down
HMGN5	-1.53	3.10E-03	Down
SLC19A2	-1.53	9.41E-04	Down
FBXO33	-1.54	5.44E-03	Down
FRRS1	-1.54	4.84E-03	Down
HSPA13	-1.54	1.05E-03	Down
FOXN2	-1.54	2.02E-04	Down
VSNL1	-1.54	3.65E-03	Down
CALB2	-1.54	1.68E-02	Down
IRAK1BP1	-1.54	1.65E-03	Down
HMGCS1	-1.54	4.51E-04	Down
SLC26A2	-1.54	7.18E-04	Down
CLK4	-1.54	3.52E-03	Down
DNAJB9	-1.54	4.79E-03	Down
CETN3	-1.54	4.48E-03	Down
TEX9	-1.54	5.89E-03	Down
COL4A3BP	-1.54	9.65E-04	Down
MIS18BP1	-1.55	1.46E-03	Down
ZNF492	-1.55	3.33E-02	Down
ADGRF3	-1.55	3.19E-02	Down

PRRG1	-1.55	9.25E-03	Down
GPR85	-1.55	2.33E-03	Down
SGO2	-1.55	1.02E-03	Down
SAMD5	-1.55	1.94E-02	Down
THAP9	-1.55	1.74E-02	Down
RASA2	-1.55	2.71E-02	Down
C8orf48	-1.55	3.07E-02	Down
HNF4G	-1.55	8.03E-03	Down
GTF2H3	-1.56	1.68E-03	Down
KBTBD3	-1.56	4.94E-02	Down
ANP32E	-1.56	5.05E-04	Down
C11orf65	-1.56	4.17E-02	Down
LCORL	-1.56	4.06E-04	Down
TMEM64	-1.56	4.33E-04	Down
CAPZA2	-1.56	1.75E-04	Down
ANXA1	-1.56	1.82E-04	Down
AMD1	-1.57	6.60E-03	Down
MMD	-1.57	1.82E-03	Down
XRCC4	-1.57	7.63E-03	Down
GJA1	-1.58	8.87E-05	Down
ASPM	-1.58	2.85E-03	Down
OTUD6B	-1.58	1.18E-03	Down
KMO	-1.58	2.18E-03	Down
ZNF214	-1.58	4.07E-02	Down
VTA1	-1.58	1.26E-04	Down
FRK	-1.59	1.28E-02	Down
NDST3	-1.59	4.23E-02	Down
SLC9A2	-1.59	1.92E-02	Down
POU3F2	-1.59	7.43E-04	Down
MARCH1	-1.59	1.60E-02	Down
GLMN	-1.59	1.38E-03	Down
OGN	-1.60	8.99E-04	Down
RESF1	-1.60	8.71E-04	Down
TFPI	-1.60	3.03E-03	Down
KLHL32	-1.60	1.89E-02	Down
PLCB4	-1.60	4.83E-03	Down
KLHL15	-1.61	1.30E-02	Down
FHOD3	-1.61	6.31E-03	Down
IFIT1	-1.61	1.22E-02	Down
AVIL	-1.61	3.33E-02	Down
SLC38A4	-1.61	4.48E-02	Down
BORCS7	-1.61	1.47E-02	Down
CDK19	-1.61	5.47E-04	Down
FRMD6	-1.62	3.64E-05	Down

LRRC40	-1.62	4.44E-05	Down
PRG4	-1.62	2.18E-02	Down
TTK	-1.62	7.99E-05	Down
FAP	-1.62	1.13E-02	Down
CENPE	-1.62	1.59E-03	Down
RP2	-1.62	2.79E-03	Down
TMEM150C	-1.62	2.75E-02	Down
KLHL41	-1.62	2.04E-03	Down
NUF2	-1.62	1.75E-04	Down
PAPSS2	-1.63	1.55E-03	Down
LRRN3	-1.63	4.55E-05	Down
MRO	-1.63	1.52E-02	Down
CNTN1	-1.63	1.90E-05	Down
ARL17B	-1.64	2.92E-03	Down
TICAM2	-1.64	8.03E-03	Down
LIN28B	-1.64	2.03E-02	Down
LRRCC1	-1.64	6.96E-04	Down
CASP3	-1.64	2.27E-04	Down
CYB5R4	-1.64	1.30E-04	Down
ZNF92	-1.65	5.75E-03	Down
G2E3	-1.66	1.39E-04	Down
ZNF22	-1.67	3.45E-03	Down
CLK1	-1.67	2.16E-04	Down
LRRC17	-1.67	4.71E-02	Down
SLC13A4	-1.67	2.01E-02	Down
USP27X	-1.68	1.24E-02	Down
GABRB2	-1.69	1.54E-04	Down
IKZF5	-1.69	1.37E-03	Down
LRRC39	-1.72	4.03E-03	Down
PPP1R3B	-1.72	8.80E-03	Down
LUM	-1.72	4.39E-05	Down
RPS6KA6	-1.72	7.80E-03	Down
ZNF75D	-1.74	8.97E-03	Down
GIN1	-1.74	2.11E-03	Down
CTSK	-1.75	4.95E-03	Down
RBFOX1	-1.75	1.09E-02	Down
IL7	-1.78	4.97E-03	Down
NAALADL2	-1.78	3.49E-02	Down
PI15	-1.79	8.76E-06	Down
TMCC3	-1.79	2.07E-02	Down
AC098582.1	-1.80	6.02E-03	Down
AC093668.3	-1.80	4.48E-03	Down
LSMEM1	-1.80	3.18E-02	Down
VAV3	-1.82	1.03E-06	Down

SELENOP	-1.82	1.79E-02	Down
CCDC126	-1.82	1.27E-03	Down
OPN1SW	-1.83	2.49E-03	Down
SYCP2	-1.85	2.60E-02	Down
ZNF93	-1.85	1.38E-02	Down
GPR89B	-1.86	9.37E-04	Down
TMSB15A	-1.86	7.28E-03	Down
AC093668.1	-1.86	3.06E-04	Down
ERICH2	-1.86	1.12E-02	Down
DNAJC27	-1.87	4.92E-04	Down
ANKLE1	-1.87	1.14E-02	Down
PHLDB2	-1.88	2.96E-03	Down
ANGPTL1	-1.88	5.44E-03	Down
AC008758.5	-1.89	3.63E-02	Down
IFI44	-1.90	3.43E-03	Down
RERG	-1.91	1.03E-02	Down
TP53INP1	-1.95	2.67E-02	Down
MANEA	-1.95	2.10E-05	Down
EIF3C	-2.01	3.35E-02	Down
CCDC152	-2.03	1.55E-02	Down
CXCR4	-2.08	3.88E-03	Down
LEAP2	-2.36	2.39E-04	Down
CCDC175	-2.50	4.87E-05	Down
GREB1	-2.52	4.56E-03	Down
PHB2	-2.66	1.30E-02	Down
CSAG2	-2.87	2.97E-03	Down
FP565260.6	-3.77	4.91E-05	Down
U2AF1	-4.12	1.65E-02	Down
XAGE1A	-12.96	1.79E-02	Down

* The significance of difference was tested by likelihood ratio test using limma (version 3.46.0) and edgeR (version 3.32.1)

Supplementary Note 1

Pangenomic analysis of Chinese gastric cancer

Yingyan Yu[1#*], Zhen Zhang[2#], Xiaorui Dong[3,#], Ruixin Yang[1#], Zhongqu Duan[3,4, #], Zhen Xiang[1], Jun Li[1], Guichao Li[2], Fazhe Yan[3], Hongzhang Xue[3], Du Jiao[3], Jinyuan Lu[3], Huimin Lu[3], Wenmin Zhang[3], Yangzhen Wei[3], Shiyu Fan[3], Jing Li[3], Jingya Jia[3], Jun Zhang[5], Jun Ji[1], Pixu Liu[6], Hui Lu[3,4], Hongyu Zhao[4], Hai Fang[7], Saijuan Chen[7], Chaochun Wei[3, 4,*], Hongzhuan Chen[8, 9*], Zhenggang Zhu[1*]

Quality control and processing of WGS dataset

Raw reads have been quality controlled (Supplementary **Fig. 1**). According to the HUPAN pipeline[1], SGA[2] was used to assemble reads into contigs due to high assembled quality and low memory consumption, which enable to assemble multiple individuals in parallel. Because the different sequencing depth between tumor tissue and matched normal mucosa, we further optimized the parameters of SGA for tumor tissue by simulated sequence data. In brief, we firstly simulated the pseudo reads with 60-fold sequencing depth from GRCh38 primary assembly sequences (including 22 autosomes and two sex chromosomes) by the NeSSM[3] according to the Illumina sequencing data of 150-bp paired-end with 400-bp insert size.

De novo assembly of genome sequences

The pseudo reads were used to perform *de novo* assembly by SGA using difference parameters (Supplementary Table 1). All the assembled results were accessed by QUAST[4] based on the GRCh38 primary assembly sequences to measure the total length of unaligned contigs and misassembled contigs. We assembled all 185 genomes of tumor tissue, resulting in final assemblies with genome sizes that ranged

from 2.69 Gb to 2.76Gb and contigs N50 sizes that range from 4.85kb to 9.96kb. Comparing the assembled results of matched normal mucosa, the assembled genome sizes of tumor tissue were significantly larger (Supplementary **Fig. 2a**), and the N50 sizes of tumor tissue were significantly larger (Supplementary **Fig.2b**). Because all female individuals are lack of Y chromosome, the assembled genome sizes of male individuals were larger than that of female individuals (Supplementary **Fig.2c**). There is no significant difference of N50 sizes between male and female individuals (Supplementary **Fig.2d**).

We randomly selected sequencing datasets of ten tumor tissues and extracted the subset of reads, which the number of reads equals to that of their matched normal mucosae. The subset reads were conducted *de novo* assembly by SGA with the optimal parameters used in matched normal mucosa[5]. Then the assembled genome sizes from different data sets were compared. After eliminating the difference of sequencing depth, the assembled genome sizes were consistent with that of matched normal mucosa, suggested that the discrepancy of assembled genome size between tumor tissue and matched normal mucosa is due to the different sequencing depth and assembled parameter of SGA (Supplementary **Fig.3**).

Identification of non-reference sequences

In current study, fully unaligned sequences are defined as contigs with no alignment to the reference human genome using MUMMER with default parameters (-c 65 -l 20) while partially unaligned sequences are defined as contigs with at least one alignment and one unaligned fragment longer than 500 bps. All assembled contigs were compared with the human reference genome, resulted in a total of 949.27 Mbp fully unaligned contigs and 1,044.04 Mbp partially unaligned contigs with identity <90% to the primary assembly sequences (where primary assembly sequences refer to sequences of 22 autosomes, two sex chromosomes and mitochondria) of GRCh38. After removing redundancies and potential contaminations, 69.16 Mbp non-reference sequences were left. By combining the non-reference genome sequences of 66.04

Mbp derived from the matched normal mucosa[1], 35,488 sequences with a total length of 80.88 Mbp representing the non-reference genomic sequences were obtained (Supplementary **Fig. 4**). The total length of fully unaligned (Supplementary **Fig.5a**) and partially unaligned (Supplementary **Fig.5b**) contigs in few genomes was obviously large due to potential contaminations. After removing the contaminated sequences from non-human species, there were ~4.80 Mbp fully unaligned contigs and ~5.67 Mbp partially unaligned contigs for each sample. There was no significant difference between females and males for both fully unaligned contigs (Supplementary **Fig.5c**) and partially unaligned contigs (Supplementary **Fig.5d**).

Helicobacter pylori (HP) and Epstein–Barr virus (EBV) are two well-known microorganisms related with gastric carcinogenesis[6]. To examine whether the non-human sequences were from HP and EBV, we aligned the fully unaligned contigs from each sample to the reference genome sequences of HP (GenBank accession: GCF_000008525.1) and EBV (GenBank accession: GCA_900474115.1) by nucmer implemented in MUMmer program[7] with the parameters "--maxmatch -l 65", respectively. Only those contigs that have any alignment with at least 95% sequence identity and 95% sequence coverage were considered as the true sequences from HP or EBV. In total, we discovered the sequences hitting of HP genome in 52 individuals (including 52 matched normal mucosae and 12 tumor tissues) (Supplementary Table 2) and EBV in 8 individuals (including 1 matched normal mucosa and 8 tumor tissues) (Supplementary Table 3). In addition, we could not identify any sequences in partially unaligned contigs aligned to the sequences of HP and EBV, suggested that the HP and EBV sequences did not integrate into human genome. For validation, we extracted the reads could not be mapped to the constructed pan-genome sequences for each sample and aligned them to reference genome sequences of two microorganisms by BWA MEM[8] with default parameters. Only these reads with CIGAR flag as "150M" in the alignment result files were counted. And the results were accordance with that of contig alignments (Supplementary **Fig.6**).

Annotation of the non-reference sequences

For non-reference sequences annotation, low-complexity repeats on non-reference sequences were firstly masked by RepeatMasker (http://www.repeatmasker.org). The masked sequences were used to conducted ab initio gene prediction by SNAP (version 2006-07-28)[9] and Augustus (version 2.5.5)[10]. The “human” and “mammal” models were selected for Augustus and SNAP prediction, respectively. RNA-seq data of gastric tissues and public human ESTs (human expressed sequences tags) were used as transcript evidence. The RNA-seq reads were assembled into contigs and aligned to the reference genome. The non-reference sequences were extracted and further removed the redundant sequences using CD-HIT. The human ESTs sequences were downloaded from GenBank (12 August 2018) and the redundant sequences were removed using CD-HIT. The resulting non-redundant transcripts sequences of RNA-seq and EST were aligned to the non-reference sequences by BLASTN. In addition, all public human protein sequences, which downloaded from GenBank (12 August 2018), were used as protein evidence. The human protein sequences were removed redundancy by CD-HIT and aligned to the non-reference sequences by BLASTX. The sequences identified around split sites were realigned by Exonerate (version 2.2.0)[11]. All results of the ab initio perditions were combined and refined with RNA and protein evidence by EVidenceModeller[12]. Predicted genes with length shorter than 100 bp were firstly removed. All the remaining genes were clustered at a global identity of 80% using CDH-HIT and further remove potential redundancy at a global identity of 50% by aligned to reference genome and transcripts. Genes with incomplete models or overlapping with repeat sequences more than 50% were further removed. Finally, 14 full-length genes were predicted from the non-reference genome sequences (Supplementary Table 4). All predicted genes were functionally annotated by searching their protein sequences using the software InterProScan[13] (version 5.39-77.0).

Construction and annotation of GCPAN

All contigs longer than 500 bps were aligned to the GRCh38 reference genome by MUMmer package (v3.23) with default parameter. Contigs with 95% or more identity as well as covering 95% sequences' lengths were considered as the reference genome sequences. The remaining contigs were assessed by QUAST (version: 4.5) [4] to extract non-reference sequences. All non-reference sequences were merged and redundant sequences were removed with a cutoff of sequence identity 90% using CD-HIT (version v4.6.7)[14] with parameters "-c 0.9 -T 16". This step was performed for the fully unaligned sequences and partially unaligned sequences separately. Then the non-redundant sequences were aligned to NT database (downloaded from NCBI, November 1st, 2019) by BLASTN with parameters "-evalue 1e-05 -outfmt 7 -max_target_seqs 1 -num_threads 16". Contigs, whose best alignments (alignment length ≥100 bp and sequence identity ≥ 60%) were not from primates, were considered as potential contaminations and were discarded for further analysis. We added the non-redundant non-reference sequences into GRCh38 to construct the sequences of GCPAN. The annotation of the human reference genome part was from GENCODE (version 30)[15].

Closing breakpoints by partially unaligned contigs

We collected all the partially unaligned contigs from the genomes of tumor tissue and matched normal mucosa, respectively, and extracted coordinates of their aligned regions on the human reference genome according to the QUAST result. The unaligned regions (≥ 500bp) of the partially unaligned contigs were extracted. To ensure sequences reliability, we only extracted sequences that attached by ≥ 500 bases alignment length with ≥ 95% identity or ≥ 100 bases alignment length with ≥ 99% identity. If both ends of a sequence could be aligned to the same chromosome region (≤ 100 kb) with the same orientation, we called the sequence as a two-end placed sequence. And if only one end of a sequence could be aligned to the human reference

genome, we called the sequence as a one-end placed sequence.

We clustered two-end-placed sequences from all samples based on their positions on the human reference genome using “BEDtools merge” [16] with the parameter “-d 10”. This procedure was conducted on tumor tissue and matched normal mucosa, separately. For the sequences in each cluster, we selected the longest sequence as represent sequence of this cluster and aligned the remaining sequences to this represent sequence by nucmer with the parameter “--maxmatch -l 65 -c 65”. All sequences that have no any alignment records were removed from this cluster. Each cluster and the cluster region on reference genome could be considered as a unique two-end placed sequence. Then we combined the unique two-end placed sequences from both primary tumor tissues and matched normal mucosae, to obtain the final dataset of two-end placed sequences. For the one-end placed sequences, we firstly removed those sequences that their coordinates on reference genome are located in the inserted positions of any two-end placed sequence. Then the remaining one-end placed sequences were clustered, merged and evaluated using the same procedures described above. The presence and absence of placed sequences in each sample were determined by aligning the raw assembled contigs to the sequences in each cluster.

By examining the coordinates of partially unaligned contigs on the human reference genome, we identified 827 two-end placed sequences with 1.41 Mbp and 1,778 one-end placed sequences with a total of 4.92 Mbp distributed across the whole genome. These sequences intersected with 690 protein-coding genes, of which 18 genes were in transcript regions (6 genes were in CDS regions and 15 genes were in UTR regions, Supplementary Table 5). The rest of 672 genes were overlapped in intron regions.

Despite the high base-pair accuracy and comprehensive representation of human reference genome, there are still hundreds of N-gaps regions accumulating more than 150 Mbp sequences in the last version GRCh38 [17]. In order to examine that whether the gaps were closed by our assembled results, we used “bedtools intersect” [16] to

determine whether the alignment coordinates of two-end-placed sequences could span the reference gaps. In total, 25 gaps of human reference genomes could be completely closed by the two-end-placed novel sequences, resulting in an increasing of 258.60 kb sequences to the reference genome. And majority of the closed gaps were detected in multiple individuals (Supplementary Table 6). In addition, by checking whether the positions of all one-placed sequences were located within 100 bp upstream or downstream of reference gaps, we found that 16 gaps could be extended in both sides, and 53 gaps could be extended in one side, resulting in an increasing of 328.97 kb sequences to non-reference genome region (Supplementary Table 7)

Comparing read mapping ratios using different reference genomes

We randomly selected 1,000 (10^3) to one million sequencing reads (10^6) from the sequencing data of an individual NA12878 from public database and mapped them by Bowtie2 (version 2.3.3.1)[18] with default parameter to GRCh38 primary assembly sequences. We found that the mapping ratio of one million reads was close enough to the overall mapping ratio using all reads (Supplementary **Fig.7a**). We then randomly sampled one million reads from each sample and mapped them to three reference sequences: GRCh38 primary assembly sequences, GRCh38 primary assembly sequences plus alternative loci and patch sequences (GRCh38_alt), and currently constructed GCPAN. The open access dataset from the **S**imons **G**enome **D**iversity **P**roject (SGDP) covered high quality genomes from 300 individuals across 142 diversity populations[19]. Of the 300 individual genomes, 263 were prepared using PCR-free library preparation and were fully available to public. We downloaded the raw reads of those 263 individuals from EBI website with the project accession number PRJEB9586. In order to evaluate the sequence mapping ratio of GCPAN as external validation, the mapping ratio of SGDP based on GCPAN was presented (Supplementary **Fig.7b**).

Positioning predicted genes on chromosomes by the third generation sequencing data

We aligned sequences of 14 predicted genes to the third generation sequencing contigs. If the global identity of a predicted gene is greater than 80% of its protein coding regions, this gene is considered as a gene supported by the third generation sequencing data. As results, 13 of the 14 genes are confirmed (Supplementary **Fig.8**).

Validation of predicted genes with proteomics data

To validate expression of predicted genes at proteome level, the MS/MS dataset of 80 diffuse gastric cancer samples was obtained from CPTAC[20], and searched with X!Tandem (version 2017.2.14)[21] against the human protein sequence database (GENECODE v30). We found that 10 of the 14 predicted genes (71.43%) were supported with at least two samples of 80 gastric cancer proteomic samples obtained from CPTAC (Supplementary Table 8). By potential functional domains prediction of 14 predicted genes, 2 out of 14 (14.29%) genes could hit at least one functional domain in Pfam database using InterProScan (Version 5.39-77) (Supplementary **Fig.9**).

Comparison of PAVs with SGDP and 90 Han datasets

Of the distributed genes, 195 genes (186 from GRCh38 and 9 predicted genes) were considered as distributed genes in both primary tumor tissues and matched gastric mucosae (Supplementary Table 9 and 10). Other 36 and 30 distributed genes were detected in matched gastric mucosa and primary tumor tissues, respectively (Supplementary Table 11 and 12). The percentage of distributed genes in predicted genes was more than 64%, while the percentage of distributed genes in GRCh38 was approximately 1% (Supplementary **Fig.10**). In addition, the majority of distributed

genes on GRCh38 were sporadically missed in a small number of samples, whereas the distributed genes of non-reference sequences evenly spread across all samples (Supplementary **Fig.11**). A total of 210 protein coding genes, including 199 GRCh38 genes and 11 predicted genes were outlined as distributed genes in SGDP dataset based on currently constructed GCPAN (Supplementary **Fig.12**). Of the 210 distributed genes, 108 genes (51.42%) were overlapped with our dataset, including 99 GRCh38 genes and 9 predicted genes (Supplementary **Fig.13, 14**).

We compared PAVs of healthy individuals of SGDP with PAVs of gastric cancer, and identified 78 distributed genes on GRCh38 with significant difference in two populations. The absent variation of *ZNF718* is omitted in the consequent analysis due to the extremely short coding region of the representative transcript we selected. The comparison of gene absence frequencies for each gene between that in the 185 gastric cancer samples and that in 263 individual genomes from SGDP was performed by Fisher's exact test, and the p-values were adjusted by multiple testing corrections (FDR ≤ 0.05, Supplementary Table 13). To validate the reliability of the gene PAVs found in gastric cancer population, we checked the PAVs in an independent dataset of 90 Han Chinese genomes (http://gigadb.org/dataset/100302). In the total of 195 distributed genes shared by the tumor and normal mucosa samples in our gastric cancer population, 146 (146/195, 74.9%) of them are absent in at least 5% of the 90 Han Chinese genomes (Supplementary **Fig.15**). In addition, 19 of them are core genes in the 90 Han Chinese genomes and the remaining 167 genes have absence in at least one individual. It meant that majority of gene PAVs found in gastric cancer population are supported by the 90 Han Chinese dataset.

For the distributed genes with significant difference in gastric cancer population and SGDP non-Asian population, we further compared absence frequencies between gastric cancer population and 90 Han Chinese dataset, and found two genes with higher absence frequency in gastric cancer population than that in 90 Han Chinese One was *ACOT1*, and another was *PRAMEF14 (*Supplementary **Fig.16***)*. In all populations, the gene absence frequencies of *PRAMEF14* were lower than those for

ACOT1. In addition, the predicted gene *GC0643* also showed higher gene abasence frequencies in Asian populations than that in SGDP non-Asian population (Supplementary **Fig.17**).

We also compared the PAVs of our dataset with dataset of non-Asian population of SGDP, and found that gene absence frequencies were significantly different in 85 distributed genes (Fisher's exact test, $P < 0.05$). Among the 85 differential genes, 82 genes were located on GRCh38 and 3 genes were predicted genes (Supplementary **Fig.18a**). We calculated the odds ratio (OR) of these 85 distributed genes and found that 75 genes (72 on GRCh38) showed OR > 1.5 (represented the absent frequencies are high in our group), while 10 genes (all are genes of GRCh38) with OR < 0.5 (represented the absent frequencies are low in our group) (Supplementary Table 14). It suggested that cancer individuals carried more absent variations of susceptible genes than that in non-Asian individuals from SGDP dataset (Supplementary **Fig.18b**).

We further compared the PAVs of Asian population of SGDP with our dataset and found that gene absence frequencies were significantly different in 71 distributed genes (Fisher's exact test, $P < 0.05$). Among the 71 differential genes, 67 genes were located on GRCh38 and 4 genes were predicted genes (Supplementary **Fig.19**). We calculated the odds ratio (OR) of those 71 distributed genes and found that 69 genes (65 on GRCh38) showed OR > 1.5 (represented the absent frequencies are high in our group), while two genes (all are genes of GRCh38) with OR < 0.5 (represented the absent frequencies are low in our group) (Supplementary Table 15).

We further compared the PAVs of East Asian population of SGDP with our dataset and found that gene absence frequencies were significantly different in 49 distributed genes (Fisher's exact test, $P < 0.05$). Among the 49 differential genes, 47 genes were located on GRCh38 and two genes were predicted genes (Supplementary **Fig.20**). We calculated the odds ratio (OR) of those 49 distributed genes and found that 48 genes (46 on GRCh38) showed OR > 1.5 (represented the absent frequencies are high in our group), while one gene (all are genes of GRCh38) with OR < 0.5

(represented the absent frequencies are low in our group).

Correlation analysis of distributed genes with clinicopathological phenotypes

For distributed genes on GRCh38, we conducted functional analysis by Metascape[22]. The nine clinical phenotypes covered gender, age, Borrmann classification, Lauren classification, tumor location, histological grade, tumor diameter, HP infection, and EBV infection. Fisher exact test was used to calculate the significance between phenotypes and gene PAVs (R 4.0.2). The 186 distributed genes on GRCh38 were enriched in 16 pathways (Supplementary **Fig.21,** Supplementary Table 16). Four genes of *ACOT1*, *GSTM1*, *UGT2B17*, and *SIGLEC14* were highly absent genes (Supplementary **Fig.22**). We further analyzed the association of clinical phenotypes of gastric cancer with all distributed genes (Supplementary **Fig.23**) and found that absence of *HLA-DRB1* and *HLA-DRB5* were mainly identified in ulcerative or infiltrative ulcerative types of Borrmann classification (Supplementary **Fig.24**). Histologically, most of cancers revealed moderate- to poor-differentiated adenocarcinoma. The absence of *PSG9* and *PSG4* was closely related to increased incidence of gastric cancer in corpus and cardia of stomach (Supplementary **Fig.23** and **25**). The larger tumor diameters, the higher frequencies of *PIM3* absence (Supplementary **Fig.23** and **26**). Gene structure analysis showed that the absence of *PIM3* occurred in exon 5 and exon 6 region. In addition, the expression level of *PIM3* was significantly lower for *PIM3* absence ($P < 0.001$, Supplementary **Fig.27**).

Furthermore, we divided absence variation of distributed genes into absence and presence groups (80% CDS coverage as cut-off) and evaluated the influence on patients' outcome. Several gene absences disclosed significant prognostic correlation, including *AZU1* (Supplementary **Fig.28**), *PRRX2* (Supplementary **Fig.29**), and *SIGLEC14* (Supplementary **Fig.30**). We noticed that the absence of *SIGLEC14* significantly influenced the prognosis of female patients, but not male patients. Most

of these cancers occurred in the cardia and corpus of the stomach with ulcerative or infiltrative ulcerative classification. Histologically, cancers with *SIGLEC14* absence showed overwhelming poorly-differentiated adenocarcinoma (73.91%) and compatible with intestinal-type cancer in Lauren classification, suggesting that the absence of *SIGLEC14* in women may play important role on carcinogenesis of non-signet-ring cell carcinoma.

Comparison of the relationship of PAVs and SVs on GRCh38

In SV analysis, over 2700 genes were identified as absence (Supplementary **Fig.31a**), and only 9 genes were homozygous DEL-SVs (Supplementary **Fig.31b**). If only homozygous gene PAVs and DEL-SVs were compared, most genes were identified as presence congruously by SV methods and PAV method. There are 203 genes identified as absence by PAV method in at least one tumor samples (Supplementary Table 17). Of these 203 genes, 33 genes were absent in at least 54 (30%) samples (Supplementary **Fig. 31c** and **31d**). Among these 33 genes, twelve genes were found significantly associated with clinical phenotypes. We extracted insertion SVs (INS-SVs) to compare with unaligned sequences in GCPAN analysis. The insertion sequences were firstly masked by TRF (version 4.09.1)[23] with the command: “trf 2 7 7 80 10 50 500 -f -h -m”. The INS-SVs with more than half repeat sequences of the SV length were filtered out. The INS-SVs sequences were mapped to GCPAN by BLASTN. The best hit of each sequence was determined according to the length and identity percentage of the aligned region. If INS-SVs sequences were mapped to unaligned sequences, each sample contained an average of 213 INS-SVs (23.22%) with an average length of 143 bps were mapped, and 925 INSs (76.78%) with an average length of 76 bps were mapped to GRCh38 (Supplementary **Fig. 31e** and **31f)**.

Upstream regulation analysis of *GC0643*-PAV-associated genes

In RNA-Seq validation study (n = 65), we divided the data into gene absence group (n = 18) and gene presence group (n = 47) based the 80% coverage of CDS 5. At gene

absence group, up-regulated expression was found in 10 genes (3 fold-change, $P <$ 0.001), while down-regulated expression was detected in 138 genes (Supplementary Table 18). We further studied the function of *GC0643* by analyzing the upstream regulators of these 10 up-regulated and 138 down-regulated genes. IPA (version 70750971, QIAGEN, 2021) upstream regulation analysis was applied (Supplementary **Fig.32,** Supplementary Table 19).

RNAScope examination

To ensure interpretable results, we used *PPIB* (Hs-PPIB-3ZZ, #701030, NM_000942.4) an endogenous housekeeping gene as positive control (Supplementary **Fig. 33**).

GC0643* examination and biological functions *in vitro

Human gastric cancer cells HGC27 and NCI-N87 were used. For knockdown of *GC0643*, shRNAs were used (Supplementary **Fig.34**). Non-targeting control shRNA was used as negative control. Stably transfected cells were then validated by mRNA (Supplementary **Fig.35**) and protein expression analysis. We further verified the functions of *GC0643* in NCI-N87 cell line（this cell line was from Western patient）. Enforced *GC0643* expression on NCI-N87 significantly increased the mRNA and protein expression levels by RT-PCR and Western blot (Supplementary **Fig. 36a, b**). Compared to control, overexpression of *GC0643* inhibited cell growth at 48 h (1.02 ± 0.06 vs 0.9 2 ± 0.05, P = 0.008) and 72 h (1.68 ± 0.07 vs 1.30 ± 0.11, P = 4.61E-05) incubation, respectively by CCK8 assay (Supplementary **Fig. 36c**). In EdU incorporation assay, the cell proliferating rate was significantly suppressed, compared to control (11.98 ± 4.27% vs 20.26 ± 5.27%, P = 0.007) (Supplementary **Fig. 36d**). It means that *GC0643* gene played a role on inhibiting cell proliferation in NCI-N87 cell line too. Regarding to apoptosis, overexpression of *GC0643* resulted in increased apoptosis in NCI-N87, compared to control (8.82 ± 1.91% vs 5.05 ± 0.29, P = 0.0001) (Supplementary **Fig. 36e**). As to cell cycle assay, compared to control, overexpression

of *GC0643* down-regulated the proportion of G1 phase (48.26 ± 0.69% vs 44.59 ± 0.82%, P = 0.001) and up-regulated the proportion of S and G2 phase (51.75 ± 0.69% vs 55.41 ± 0.82%, P = 0.001). It means that *GC0643* overexpression led to G2/M arrest in NCI-N87 cell line (Supplementary **Fig. 36f**).

The downstream pathways regulated by *GC0643* overexpression

The total RNA was extracted from $HGC27^{GC0643}$ and $HGC27^{Vector}$ using Total RNA Extraction Reagent (Vazyme, China) for transcriptome sequencing. Three replicates were set for each group. Qubit RNA Assay Kit in Qubit 2.0 Flurometer (Agilent Technologies, USA) was used for preliminary RNA quantification. The RNA Nano 6000 Assay Kit of the Bioanalyzer 2100 system (NEB, USA) was used for measuring RIN value. NEBNext® Ultra RNA Library Prep Kit for Illumina (NEB, USA) was used for library construction. Illumina Novaseq 6000 (Illumina, USA) was used for transcriptome sequencing. The differential expression analysis was performed using limma (version 3.46.0) and edgeR (version 3.32.1). The threshold was set as fold change >1.5 or < -1.5 and P < 0.05 (Supplementary Table 20).

References

1. Duan Z*, et al.* HUPAN: a pan-genome analysis pipeline for human genomes. *Genome Biol* 20, 149 (2019).

2. Simpson JT, Durbin R. Efficient de novo assembly of large genomes using compressed data structures. *Genome Research* 22, 549-556 (2012).

3. Jia B, Xuan L, Cai K, Hu Z, Ma L, Wei C. NeSSM: a Next-generation Sequencing Simulator for Metagenomics. *PLoS One* 8, e75448 (2013).

4. Gurevich A, Saveliev V, Vyahhi N, Tesler G. QUAST: quality assessment tool for genome assemblies. *Bioinformatics* 29, 1072-1075 (2013).

5. Gao L*, et al.* The tomato pan-genome uncovers new genes and a rare allele regulating fruit flavor. *Nat Genet* 51, 1044-1051 (2019).

6. Uemura N*, et al.* Helicobacter pylori infection and the development of gastric cancer. *N Engl J Med* 345, 784-789 (2001).

7. Kurtz S*, et al.* Versatile and open software for comparing large genomes. *Genome Biol* 5, R12 (2004).

8. Li H, Durbin R. Fast and accurate short read alignment with Burrows-Wheeler transform. *Bioinformatics* 25, 1754-1760 (2009).

9. Korf I. Gene finding in novel genomes. *BMC Bioinformatics* 5, 59 (2004).

10. Stanke M, Steinkamp R, Waack S, Morgenstern B. AUGUSTUS: a web server for gene finding in eukaryotes. *Nucleic Acids Res* 32, W309-312 (2004).

11. Slater GS, Birney E. Automated generation of heuristics for biological sequence comparison. *BMC Bioinformatics* 6, 31 (2005).

12. Haas BJ*, et al.* Automated eukaryotic gene structure annotation using EVidenceModeler and the Program to Assemble Spliced Alignments. *Genome Biol* 9, R7 (2008).

13. Jones P*, et al.* InterProScan 5: genome-scale protein function classification. *Bioinformatics* 30, 1236-1240 (2014).

14. Fu LM, Niu BF, Zhu ZW, Wu ST, Li WZ. CD-HIT: accelerated for clustering the next-generation sequencing data. *Bioinformatics* **28**, 3150-3152 (2012).

15. Harrow J, *et al.* GENCODE: the reference human genome annotation for The ENCODE Project. *Genome Res* **22**, 1760-1774 (2012).

16. Quinlan AR, Hall IM. BEDTools: a flexible suite of utilities for comparing genomic features. *Bioinformatics* **26**, 841-842 (2010).

17. Schneider VA, *et al.* Evaluation of GRCh38 and de novo haploid genome assemblies demonstrates the enduring quality of the reference assembly. *Genome Res* **27**, 849-864 (2017).

18. Langmead B, Salzberg SL. Fast gapped-read alignment with Bowtie 2. *Nature Methods* **9**, 357-U354 (2012).

19. Mallick S, *et al.* The Simons Genome Diversity Project: 300 genomes from 142 diverse populations. *Nature* **538**, 201-206 (2016).

20. Mun DG, *et al.* Proteogenomic Characterization of Human Early-Onset Gastric Cancer. *Cancer Cell* **35**, 111-124 e110 (2019).

21. Craig R, Beavis RC. TANDEM: matching proteins with tandem mass spectra. *Bioinformatics* **20**, 1466-1467 (2004).

22. Zhou Y, *et al.* Metascape provides a biologist-oriented resource for the analysis of systems-level datasets. *Nat Commun* **10**, 1523 (2019).

23. Benson G. Tandem repeats finder: a program to analyze DNA sequences. *Nucleic Acids Res* **27**, 573-580 (1999).
