# Supplementary material for: Pangenomic analysis of Chinese gastric cancer
Source: Nat Commun. 2022 Sep 15;13:5412. doi: 10.1038/s41467-022-33073-7 (PMC9477819; doi:10.1038/s41467-022-33073-7)
Supplement: Supplementary file 2 — Reporting Summary [file 41467_2022_33073_MOESM2_ESM.pdf]

nature portfolio

Corresponding author(s): Yingyan Yu

Last updated by author(s): Aug 23, 2022

# Reporting Summary

Nature Portfolio wishes to improve the reproducibility of the work that we publish. This form provides structure for consistency and transparency in reporting. For further information on Nature Portfolio policies, see our Editorial Policies and the Editorial Policy Checklist.

## Statistics

For all statistical analyses, confirm that the following items are present in the figure legend, table legend, main text, or Methods section.

| n/a | Confirmed | |
|---|---|---|
| ☐ | ☒ | The exact sample size (*n*) for each experimental group/condition, given as a discrete number and unit of measurement |
| ☐ | ☒ | A statement on whether measurements were taken from distinct samples or whether the same sample was measured repeatedly |
| ☐ | ☒ | The statistical test(s) used AND whether they are one- or two-sided<br>*Only common tests should be described solely by name; describe more complex techniques in the Methods section.* |
| ☐ | ☒ | A description of all covariates tested |
| ☐ | ☒ | A description of any assumptions or corrections, such as tests of normality and adjustment for multiple comparisons |
| ☐ | ☒ | A full description of the statistical parameters including central tendency (e.g. means) or other basic estimates (e.g. regression coefficient) AND variation (e.g. standard deviation) or associated estimates of uncertainty (e.g. confidence intervals) |
| ☐ | ☒ | For null hypothesis testing, the test statistic (e.g. $F$, $t$, $r$) with confidence intervals, effect sizes, degrees of freedom and $P$ value noted<br>*Give P values as exact values whenever suitable.* |
| ☒ | ☐ | For Bayesian analysis, information on the choice of priors and Markov chain Monte Carlo settings |
| ☒ | ☐ | For hierarchical and complex designs, identification of the appropriate level for tests and full reporting of outcomes |
| ☒ | ☐ | Estimates of effect sizes (e.g. Cohen's *d*, Pearson's *r*), indicating how they were calculated |

*Our web collection on statistics for biologists contains articles on many of the points above.*

## Software and code

Policy information about availability of computer code

| | |
|---|---|
| Data collection | No software was used for the collection of the data in the paper. |
| Data analysis | We used HUPAN (version 1.02) for pan-genome analysis which was published in Genome Biology (2019) and is freely available at http://cgm.sjtu.edu.cn/hupan/ and https://github.com/SJTU-CGM/HUPAN. Other custom code for this study are available at https://github.com/SJTU-CGM/CPAN.<br>SGA (Version 0.10.15); MAKER (version 2.31.9); GENCODE (version 30); trimmomatic (version 0.32); HISAT2 (version 2.1.0); featureCounts(version 2.0.0); Bowtie2(version 2.4.3); GATK(version 4); MUMmer(version4.0); BWA MEM(version 0.7.17-r1188); Metascape(version 3.5). |

For manuscripts utilizing custom algorithms or software that are central to the research but not yet described in published literature, software must be made available to editors and reviewers. We strongly encourage code deposition in a community repository (e.g. GitHub). See the Nature Portfolio guidelines for submitting code & software for further information.

## Data

Policy information about availability of data

All manuscripts must include a data availability statement. This statement should provide the following information, where applicable:

- Accession codes, unique identifiers, or web links for publicly available datasets
- A description of any restrictions on data availability
- For clinical datasets or third party data, please ensure that the statement adheres to our policy

The raw sequencing data of genomic and transcriptomic sequencing reported in this paper have been deposited in the Genome Sequence Archive in National Genomics Data Center, China National Center for Bioinformation (GSA-Human)[https://ngdc.cncb.ac.cn/gsa-human]. HRA002344 [https://ngdc.cncb.ac.cn/gsa-

human/browse/HRA002344] for normal gastric mucosa, and HRA002333 [https://ngdc.cncb.ac.cn/gsa-human/browse/HRA002333] for gastric cancer. The raw sequencing data are available under restricted access due to data privacy laws. Readers can get access to data by sending request to corresponding authors. Data will be available within a week once the access has been granted. The processed data and result files are available on the website http://cgm.sjtu.edu.cn/cpan/GCPAN.html. The 90 Han Chinese data [http://gigadb.org/dataset/100302] and SGDP data [https://www.ebi.ac.uk/ena/browser/view/PRJEB9586] for supporting the findings of this study are open accessible. The proteomics data for gastric cancer (PDC000214) [https://pdc.cancer.gov/pdc/browse/filters/primary_site:Stomach from the CPTAC project, and the long-read sequencing data of humans (PRJNA301527, PRJA339722, PRJNA530217, and PRJNA551670) [https://www.ncbi.nlm.nih.gov/sra] for positioning the non-reference genes to corresponding chromosomes are open accessible.

# Field-specific reporting

Please select the one below that is the best fit for your research. If you are not sure, read the appropriate sections before making your selection.

☒ Life sciences ☐ Behavioural & social sciences ☐ Ecological, evolutionary & environmental sciences

For a reference copy of the document with all sections, see nature.com/documents/nr-reporting-summary-flat.pdf

# Life sciences study design

All studies must disclose on these points even when the disclosure is negative.

| | |
|---|---|
| Sample size | A total of 185 paired gastric cancer and paired normal mucosae are included in the study. No sample-size was calculated |
| Data exclusions | No data were excluded from the analysis. |
| Replication | To confirm our conclusion, at least 3 biological replicates were performed for each experiment in Figure 6 and Supplementary Fig.36. Each experiment was repeated to ensure that the experimental conditions, experimenter and statistical methods were consistent to get a credible conclusion. |
| Randomization | In this study, the samples used for omics study were randomly enrolled in. |
| Blinding | Our study is focused on genetic features of gastric cancer. Investigators were blinded to group allocation during data collection and analysis for experiments other than those involving patients. |

# Reporting for specific materials, systems and methods

We require information from authors about some types of materials, experimental systems and methods used in many studies. Here, indicate whether each material, system or method listed is relevant to your study. If you are not sure if a list item applies to your research, read the appropriate section before selecting a response.

## Materials & experimental systems

| n/a | Involved in the study | |
|---|---|---|
| ☐ | ☒ | Antibodies |
| ☐ | ☒ | Eukaryotic cell lines |
| ☒ | ☐ | Palaeontology and archaeology |
| ☒ | ☐ | Animals and other organisms |
| ☐ | ☒ | Human research participants |
| ☒ | ☐ | Clinical data |
| ☒ | ☐ | Dual use research of concern |

## Methods

| n/a | Involved in the study | |
|---|---|---|
| ☒ | ☐ | ChIP-seq |
| ☒ | ☐ | Flow cytometry |
| ☒ | ☐ | MRI-based neuroimaging |

## Antibodies

| | |
|---|---|
| Antibodies used | 1. HRP-conjugated mouse anti DDDDK-Tag (1:5000, ABClone, AE024, China); 2. HRP-conjugated GAPDH monoclonal antibody (1:5000, PROTEINTECH, HRP-60004, USA) |
| Validation | 1. validation: https://abclonal.com.cn/catalog/AE024; 2. validation: https://www.ptgcn.com/products/GAPDH-Antibody-HRP-60004.htm |

## Eukaryotic cell lines

Policy information about cell lines

| | |
|---|---|
| Cell line source(s) | 1. HGC-27 GASTRIC CANCER CELL LINE from National Collection of Authenticated Cell Cultures (TCHu 22)<br>2. NCI-N87 GASTRIC CANCER CELL LINE from National Collection of Authenticated Cell Cultures (TCHu 130) |

| | |
|---|---|
| Authentication | All cell lines were authenticated by short tandem repeat (STR) analysis at National Collection of Authenticated Cell Cultures. |
| Mycoplasma contamination | The authors confirm that the cell line is negative for mycoplasma. |
| Commonly misidentified lines (See ICLAC register) | No commonly misidentified cell lines were used in this study. |

## Human research participants

Policy information about studies involving human research participants

| | |
|---|---|
| Population characteristics | A total of 185 gastric cancer samples were collected for gastractomy specimen, which were pathologically confirmed, and proved not to have other types of cancer. |
| Recruitment | The samples were collected randomly after operation. |
| Ethics oversight | The study was approved by the institutional review board of Ruijin Hospital, Shanghai Jiao Tong University School of Medicine. |

Note that full information on the approval of the study protocol must also be provided in the manuscript.
